# The mannose receptor on sinusoidal lining cells mediates two-step bacterial clearance in the human spleen

**DOI:** 10.1038/s41467-026-72430-8

**Published:** 2026-04-29

**Authors:** Neama Alnabati, Francesco Flandi, Tareq Al Saoudi, Giulia Cattabriga, Talia Richardson, Alessia Gennai, Daniele Ghezzi, Ryan G. Hames, John Isherwood, Trisha Kanani, Zydrune Jasiunaite, Shiying Tang, Giuliana Germinario, Giorgia Radi, Francesca Rizzo, Katrin Schilcher, Christopher D. Bayliss, Romina Camilli, Marco Caprini, Carola Parolin, Stefano Fedi, Wen Y. Chung, Giuseppe Garcea, Enrico Giampieri, Gastone Castellani, Kornelis Straatman, Stefano Bruno, Claudia Trappetti, Matteo Ravaioli, Ashley R. Dennison, Luisa Martinez-Pomares, Marco R. Oggioni

**Affiliations:** 1 College of Life Sciences, School of Biological and Biomedical Sciences, University of Leicester, Leicester, UK; 2 Department of Biology, Faculty of Science, Umm Al-Qura University, Makkah, Saudi Arabia; 3 Department of Pharmacy and Biotechnology, University of Bologna, Bologna, Italy; 4 Department of Hepato-Pancreato-Biliary Surgery, University Hospitals of Leicester NHS Trust, Leicester, UK; 5 Department of Medical Biotechnology, University of Siena, Siena, Italy; 6 IRCCS Azienda Ospedaliero-Universitaria di Bologna, Bologna, Italy; 7 Department of Medical and Surgical Sciences, University of Bologna, Bologna, Italy; 8 Istituto Superiore di Sanità, Roma, Italy; 9 IRCCS Istituto delle Scienze Neurologiche di Bologna, Bologna, Italy; 10 Advanced Imaging Facility, University of Leicester, Leicester, UK; 11 Department of Food and Drug, University of Parma, Parma, Italy; 12 School of Life Sciences, University of Nottingham, Nottingham, UK

**Keywords:** Infection, Microbiology, Adaptive immunity, Immunohistochemistry

## Abstract

The human spleen plays a critical role in clearing bacteria from the bloodstream, particularly during septicaemia; yet the cellular mechanisms underlying this function remain poorly defined. Using a dual translational approach including ex vivo perfusion of human spleens and splenic primary cell cultures, we identify a previously unrecognised division of tasks in splenic antibacterial defence. In the human splenic red pulp, sinusoidal lining cells capture and retain bacteria via the CD206 mannose receptor. Subsequently, the bactericidal activity by red pulp macrophages involves the scavenger receptor MARCO. This two-step process ensures efficient removal of encapsulated pathogens as inhibition of CD206 receptor impacted not only *Streptococcus pneumoniae* killing, independent of the capsular serotype, but also on *Klebsiella pneumoniae* and *Escherichia coli* revising the mechanism of pathogen clearance in the human spleen. Trial registration: REC 18/EM/0057 (NCT04620824) and CE: 668/2023/Sper/AOU.

## Abstract (web summary)

Serious life threatening infections occur when bacteria enter the bloodstream, yet how the spleen is able to clear these pathogens remains unclear. Alnabati et al., show that within the red pulp, sinusoidal lining cells capture and retain bacteria via the CD206 mannose receptor.

## Introduction

The human spleen is the main secondary lymphoid organ involved in the control of systemic infection^1^, particularly through its critical role in the removal of encapsulated bacteria from the circulation^2^. Loss of the spleen by splenectomy, and other forms of functional asplenia, expose patients to the risk of overwhelming sepsis, most commonly caused by capsulated bacteria such as *Streptococcus pneumoniae*^3–5^. This has led to the inclusion of asplenia as a key indication for vaccination in many countries^6^. Despite this, the pneumococcus remains the leading cause of post-splenectomy sepsis^7^. Although the central role of the spleen in antibacterial clearance is established, the underlying cellular mechanisms remain critically understudied. This situation contrasts with the well-documented pathways mediating the removal of *Plasmodium falciparum*-infected red blood cells (RBCs) unveiled by exploiting the translational ex vivo human spleen perfusion model^8^.

In most human tissues, tissue resident macrophages express the mannose receptor (MRC1/CD206)^9^. Importantly, this paradigm does not apply to the human spleen. Neither the CD68^+^CD163^+^ red pulp macrophages (RPMs) nor the CD68^+^CD169^+^ perifollicular capillary sheath macrophages (PCSAMs) express the mannose receptor^9^. Instead, the CD206 receptor is expressed by the sinusoidal lining cells, which make up most of the structure of the human splenic red pulp^9,10^. Splenic sinuses are the walled irregular structures draining the open circulation of the human splenic red pulp and are lined by sinus endothelia, alternatively named sinusoidal lining cells (CD206^+^, LYVE-1^+^, CD141^+^)^10–12^. The rationale for this unusual CD206 expression in the human spleen is still unknown. The CD206 mannose receptor is a key molecule involved in binding to bacteria, viruses and parasite-infected cells, playing a key role in the interface between the human host and microbes. Binding of the mannose receptor to bacterial surface sugars, including the capsular polysaccharides of *S. pneumoniae* and the lipopolysaccharide (LPS) of *Klebsiella pneumoniae*^13^, is one of the main mechanisms by which macrophages recognise these pathogens. Beyond bacteria, CD206 also mediates the uptake of dengue virus and hepatitis B virus into host cells^14,15^. Recent studies in sickle cell disease revealed that patient red blood cells (RBCs) display elevated surface high-mannose N-glycans that are recognised by CD206, and that increased mannose exposure correlates with anaemia, indicating enhanced in vivo clearance driven by mannose recognition. Furthermore, oxidative stress and infection with *P. falciparum* induce exposure of high-mannose ligands on RBCs, highlighting a key role for mannose-dependent clearance in maintaining RBCs homoeostasis and conferring protection against malaria^16^.

Investigation of host-pathogen interactions in the spleen, using murine infection models, non-human primates, and ex vivo porcine spleen perfusion, has revealed that systemic *S. pneumoniae* infections were predominantly cleared by splenic red pulp macrophages^17–20^. While the spleen was the main organ responsible for bacterial clearance, rare splenic replication in permissive macrophages facilitated re-seeding to the bloodstream and initiation of systemic infection^17,18^. The ex vivo human spleen perfusion model confirmed bacterial clusters in human splenic macrophages^20^. However, the restricted expression of the mannose to the sinusoidal lining cells raises questions about pathogen elimination and highlights a gap in understanding human-specific clearance. Here, using a dual translational platform consisting of human spleen ex vivo perfusion alongside co-cultures of primary splenic macrophages and sinusoids, we show that CD206-mediated capture by sinusoidal lining cells, followed by macrophage clearance, underlies the human spleen’s distinctive ability to filter, retain, and clear encapsulated bacteria. This human-specific adaptation sheds new light on splenic innate immune surveillance and has implications for vaccine efficacy and host-targeted therapies.

## Results and discussion

### Cellular marker distribution in the human spleen

**Fig. 1:**
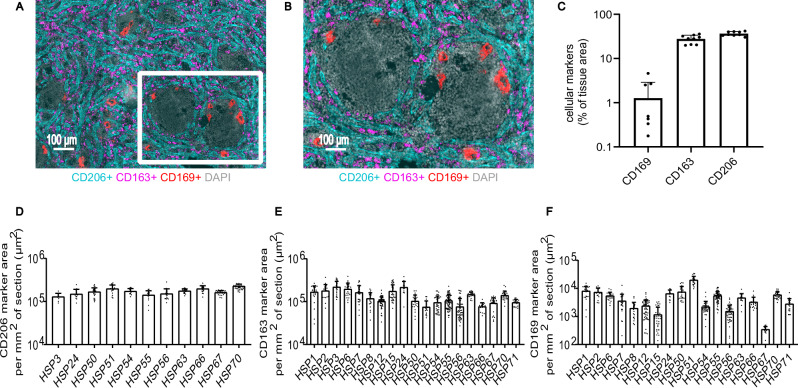
Quantification of the major cell populations in the red pulp of the human spleen. Graphs report mean and standard deviation (SD). **A** Representative high-content fluorescent microscopy image of a spleen section stained for CD206^+^ sinusoidal lining cells (cyan), CD163^+^ RPMs (magenta), and CD169^+^ PCSAMs (red), with nuclear counterstaining using DAPI (grey). **B** Zoomed-in region of (**A**) showing higher magnification of the area analysed. **C** Quantification of marker-positive areas relative to the total DAPI area (μm²). Each dot represents one analysed image region (“stamp”). Three stamps were analysed per spleen from three independent human spleens (biological replicates). **D** Inter-individual variability in CD206^+^ sinusoidal lining cells. Each bar represents one independent human spleen (biological replicate; *n* = 11 spleens). For each spleen, 9–28 image regions (“stamps”) were analysed at different perfusion timepoints (technical replicates). Dots represent individual stamps. No statistical comparison was performed; data are shown descriptively. **E** Inter-individual variability in CD163^+^ RPMs. Each bar represents one independent human spleen (biological replicate; *n* = 19 spleens). For each spleen, 11–73 image regions (“stamps”) were analysed (technical replicates). Dots represent individual stamps. No statistical comparison was performed. **F** Inter-individual variability in CD169^+^ PCSAMs. Each bar represents one independent human spleen (biological replicate; *n* = 18 spleens). For each spleen, 12–73 image regions (“stamps”) were analysed (technical replicates). Dots represent individual stamps. No statistical comparison was performed. Source data are provided as a Source Data file. Spleens in figures are listed in Supplementary Table 5.

To investigate how CD206 expression on sinusoidal lining cells contributes to the clearance of encapsulated bacteria in the human spleen, we used a translational ex vivo human spleen perfusion model^20^ using organs sourced through the TIMID trial (REC 18/EM/0057; ClinicalTrials.gov NCT04620824) and primary cells through the MOSIE trial (CE: 668/2023/Sper/AOUBo). This approach enabled direct analysis of cellular localisation and function in the human spleen. Microscopy mapped the three red pulp markers, CD163, CD169, and CD206, to three distinct cell types: CD68^+^CD163^+^ red pulp macrophages (RPMs), CD68^+^CD169^+^ perifollicular capillary sheath–associated macrophages (PCSAMs) (Supplementary Figs. 1A, B), and sinusoidal lining CD206^+^LYVE-1^+^CD31^-^ cells^9,21,22^ (Figs. 1A, B; and Supplementary Fig. 1C, D). High-content scanning fluorescent microscopy revealed that RPMs and CD206^+^ sinusoidal lining cells^10^ dominate the red pulp (Fig. 1A, B), while CD169^+^ macrophages clustered in sheaths around perifollicular capillaries, often forming ring-like structures adjacent to white pulp follicles^21^ (Fig. 1A, B). CD206^+^ sinusoidal lining cells, CD163^+^ RPMs, and CD169^+^ macrophages occupied on average 36.5% (SD 4.13), 27.7% (SD 6.1), and 1.3% (SD 1.6) of the total splenic section area, with ranges of 30.3–42.0%, 19.9–35.8%, and 0.07–4.6% respectively (Fig. 1C). Thus, sinusoidal lining cells are the most abundant CD206-expressing population in the human spleen. Marker abundance varied between spleens, up to 1.2-fold for CD206^+^, 2.7-fold for CD163^+^, and intriguingly 12.1-fold for CD169^+^ macrophages (Fig. 1D–F), the latter reflecting differences in follicle density and microanatomy between organs (Supplementary Fig. 1E). Importantly, serial biopsies from the same spleen at different infection stages showed no substantial variation in CD163 or CD206 levels (Supplementary Fig. 1F, G), confirming the stability of these populations during the perfusion and bacterial challenge.

Taken together, these observations reveal a compartmentalised immune architecture in the human spleen, in which CD206⁺ sinusoidal lining cells form an extensive blood-facing capture network, CD163⁺ macrophages populate the red pulp to remove pathogens, senescent cells, and debris, and rare CD169⁺ macrophages localise to perifollicular regions, where they may regulate antigen entry into the white pulp^23^. This organisation contrasts with the murine spleen, where red pulp macrophages themselves express CD206^24,25^ and directly participate in pathogen recognition and uptake. In mice, CD206 is also expressed by splenic endothelial cells^25^, indicating that, while both species share CD206 expression in non-vascular endothelial compartments, the cellular distribution of this receptor differs markedly. Together, these findings underscore a species-specific anatomical and functional reassignment of CD206 in splenic immunity.

### Ex vivo organ perfusion

**Fig. 2:**
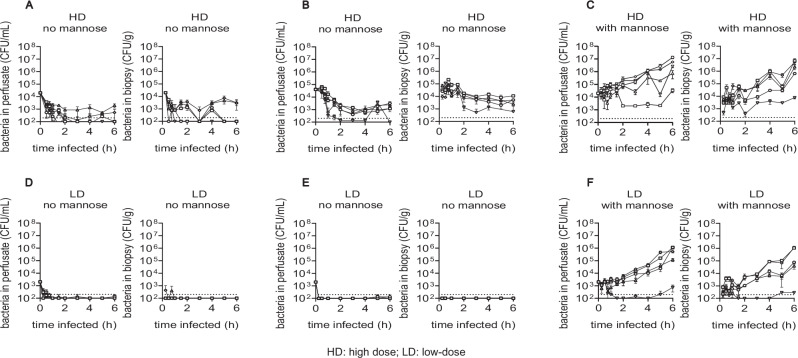
Bacterial counts during ex vivo spleen perfusion. Graphs show mean +/- SD. Bacterial counts for six human spleens perfused with a mixture of pneumococcal isolates (Mix 1). All organs were challenged with an equal mix of five *S. pneumoniae* isolates (type 2: upward triangle; type 4: downward triangle; type 5: circle; type 6 A: diamond; type 19 F: square) at a cumulative dose of 1 × 10^8^ CFU (high-dose; HD) or 1 × 10^7^ CFU (low-dose; LD). Bacterial counts are reported for millilitres (mL) of perfusion liquid and gram (g) of tissue biopsy within each panel. Panels **A**–**F** correspond to the six individual spleens. Panel’s report both perfusate (left) and biopsy (right) bacterial counts: **A** high-dose perfusion (no mannose treatment); **B** high-dose perfusion (no mannose treatment); **C** high-dose perfusion with 5 mM mannose added to the perfusion liquid 30 minutes before bacterial challenge; **D** low-dose perfusion (no mannose treatment); **E** low-dose perfusion (no mannose treatment); **F** low-dose perfusion with 5 mM mannose added to the perfusion liquid 30 minutes before bacterial challenge. Bacterial counts are reported over six hours of infection. Each dot represents the mean CFU value for one sample at a given time point, calculated from three independent plating technical replicates of that biopsy or perfusate sample. Dotted lines represent the limit of detection (2×10^2^ CFU). Source data are provided as a Source Data file. Spleens perfused in these panels are listed in Supplementary Table 5.

Using the TIMID trial, human spleens were sourced for ex vivo normothermic organ perfusion to assess the splenic antibacterial clearance capacity. After cannulation and anticoagulant perfusion, spleens were transported on ice, connected to a normothermic circuit, and perfused with polymerised haemoglobin as an oxygen carrier. Importantly, this system preserves splenic architecture and blood flow, allowing physiological assessment of bacterial infection. Systemic infection was simulated by introducing *S. pneumoniae* directly into the perfusion liquid, mimicking hematogenous invasive human infections (Supplementary Table 1), and serial biopsies and perfusate samples were taken over time to monitor the infection dynamics^20^. Across experiments, the spleen demonstrated a robust filtration capacity, removing over 90% of the inoculum within 60 minutes (Fig. 2A, B, D, E). Clearance kinetics were comparable when spleens were challenged with individual strains or with mixed serotype inocula (Supplementary Table 1, Supplementary Table 2). Given, the limited availability of human organ, mixed infections guaranteed a more controlled experimental set-up allowing for both simultaneous testing of virulent and avirulent serotypes while minimising any potential impact of type-specific immunity of organ donors (Supplementary Table 3). At a high-dose (1 × 10^8^ cumulative CFU) of five equally dosed serotypes (2, 4, 5, 6 A, 19 F; Mix 1), clearance was equally efficient across strains, regardless of invasive disease potential (Figs. 2A, B)^26^. Consistently, the association of the bacteria with CD163^+^ RPMs and the less abundant CD169^+^ PCSAMs, based on image analysis, did not vary among serotypes (Supplementary Fig. 2A). Moreover, neither the rapidity nor the extent of bacterial clearance in the human spleen was affected by the donor-derived antibacterial antibody levels in the perfusion fluid (Supplementary Table 1). Instead, at a lower dose (1 × 10^7^ cumulative CFU), all serotypes were cleared from both perfusate and tissue within 30 min (Figs. 2D, E). These kinetics underscore the spleen’s extraordinary capacity for rapid, non-discriminatory removal of encapsulated bacteria even without high antibody titres, indicating that early splenic clearance is antibody-independent. This observation aligns with the clinical reality of overwhelming post-splenectomy infection (OPSI), where the absence of splenic tissue dramatically impairs early pathogen removal^27^. The uniformity of clearance across serotypes highlights a fundamental property of splenic immunity: its filtering capacity operates independently of serotype-specific immune history, a fact critical to understanding infection risk in asplenic patients^27^.

### Contribution of the mannose receptor on sinusoidal lining cells to bacterial clearance in the human spleen

In most tissues, CD206 is expressed by macrophages and dendritic cells and mediates bacterial binding and uptake^13,28^. In the human spleen, however, the expression of CD206 (Figs. 1A, B; and Supplementary Fig. 2B, C) is predominantly detected on sinusoidal lining cells, a distribution that raises an important mechanistic question: does splenic clearance rely on CD206-mediated capture by these sinusoidal lining cells rather than by macrophages?

To address this, we built on in vitro evidence that free mannose can block CD206–pathogen binding^13^, and perfused spleens with 5 mM mannose alongside the 10–20 mM glucose required for host and bacterial metabolism. When challenged with a high pneumococcal dose (1x10^8^ CFU), mannose-treated spleens failed to clear the bacteria, with progressive bacterial accumulation in both the perfusate and tissue (Fig. 2C). At a lower challenge dose (1x10^7^ CFU), which was usually cleared within minutes, killing was completely abolished; bacteria instead proliferated rapidly (Fig. 2F). These results identify the carbohydrate-binding activity of CD206 on sinusoidal lining cells as an essential initial step in pathogen recognition. Since the presence of glucose in the perfusion liquid and the cell culture medium blocks completely the pneumococcal mannose metabolism through carbon catabolite repression^29^ (Supplementary Fig. 3C), the observed escape from host-mediated clearance cannot be ascribed to an effect of mannose on bacteria. Rather, it reflects direct interference with the receptor-mediated capture.

**Fig. 3:**
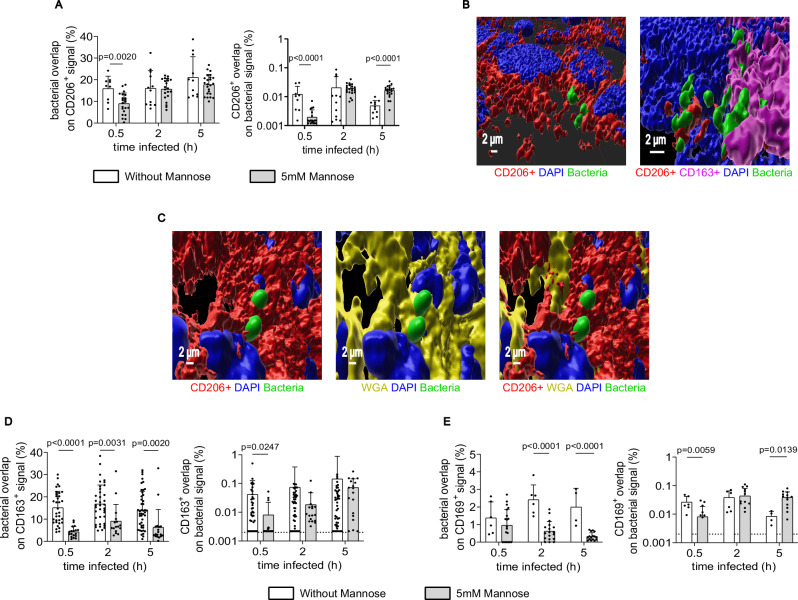
Association of pneumococci with splenic macrophages and sinusoidal lining cells during ex vivo perfusion, with and without mannose supplementation. Graphs report mean +/- SD. **A** Quantification of the percentage of tissue-associated pneumococci colocalizing with CD206^+^ sinusoidal lining cells at different time points post-infection (hour) (left), and analysis of the sinusoidal area occupied by bacteria (right) under control (white bars) and mannose-supplemented conditions (grey bars). Control data derive from *n* = 2 pooled human spleens (9–12 stamps per time point, technical replicates); mannose data from *n* = 2 pooled spleens (21–24 stamps). **B** 3D confocal reconstruction showing CD206 (red) expression in relation to pneumococci (green) (left) and their interaction with CD163^+^ macrophages (magenta) (right). Nuclei are stained with DAPI (blue). Images represent ≥9 fields of view from *n* = 2 independent spleens infected with serotypes 4 and 19 F, with similar results in all cases. **C** WGA (yellow) staining of spleen section showing localisation of CD206 (red) and bacteria (green) on sinusoidal lining cells. Nuclei are labelled using DAPI staining (blue). Images represent 2 fields of view from *n* = 2 infection time points (0 and 6 h), with similar results in all cases. **D** Quantification of pneumococcal association with CD163⁺ RPMs at 30 minutes, 2 h, and 5 h post-infection (left), and analysis of the macrophage area occupied by bacteria (right) under control conditions (white bars) and mannose supplementation (grey bars). Control data from *n* = 3 spleens (34–45 stamps); mannose from *n* = 2 spleens (16–18 stamps) per time point. **E** Colocalization of pneumococci with CD169^+^ perifollicular macrophages (left), and analysis of the CD169^+^ area occupied by bacteria (right) during normal perfusion and mannose supplementation. Control data from *n* = 3 spleens (4–6 stamps); mannose from *n* = 2 spleens (16–20 stamps) per time point. Time is expressed in hour (h). Dotted lines indicate detection limits (E: 0.002%). Marker associations were calculated in Fiji (v1.53) using threshold-defined regions of interest and particle analysis. Statistical significance was determined for each time point by two-sided unpaired parametric t-test (*p* values for all comparisons are shown directly in the figure). Source data are provided as a Source Data file. Spleens analysed are listed in Supplementary Table 5.

To complement the analysis of viable bacteria, we used high-content scanning fluorescence microscopy to examine the association of pneumococci with splenic macrophages and sinusoidal lining cells in tissue sections obtained during ex vivo perfusion. This analysis reinforced the clearance findings: across all time points, 16–21% of tissue-associated bacteria colocalized with CD206 (Fig. 3A), a proportion that remained stable despite a 90% reduction in total bacterial load during the first hour (Fig. 2). Correspondingly, the area of sinusoidal tissue occupied by bacteria showed no decline (Fig. 3A). Three-dimensional confocal reconstructions revealed discrete clusters of CD206 in direct contact with bacteria (Fig. 3B). In mannose-treated spleens, bacterial association with CD206 and CD206–bacteria colocalization fell sharply at early time points, coinciding with higher viable counts and broader sinusoidal lining cell distribution later (Fig. 3A).

To determine the fate of captured bacteria, we performed wheat germ agglutinin (WGA) staining, which confirmed that CD206^+^ sinusoidal lining cells do not internalise bacteria (Fig. 3C). Instead, captured bacteria remained extracellular, suggesting that these cells act as stationary traps. Additional quantification showed that 20% of pneumococci colocalized with CD163^+^ RPMs at 30 min, 2 h, and 5 h post-infection, and that mannose treatment significantly reduced this association (Fig. 3D). Although CD169^+^ macrophages were less abundant, their bacterial association was also diminished by mannose, especially at later stages of infection (Fig. 3E). At the later time points (2–5 h) (Fig. 3A), the apparent increase in CD206-colocalized bacteria in the mannose-treated spleens likely reflects higher bacterial loads in the tissue and perfusate due to impaired clearance, rather than true receptor-mediated binding. Consistently with the concurrent reduction in bacteria–macrophage colocalization (Figs. 3D, E), these data suggest that bacteria are less efficiently transferred to, or cleared by, macrophages under these conditions.

**Fig. 4:**
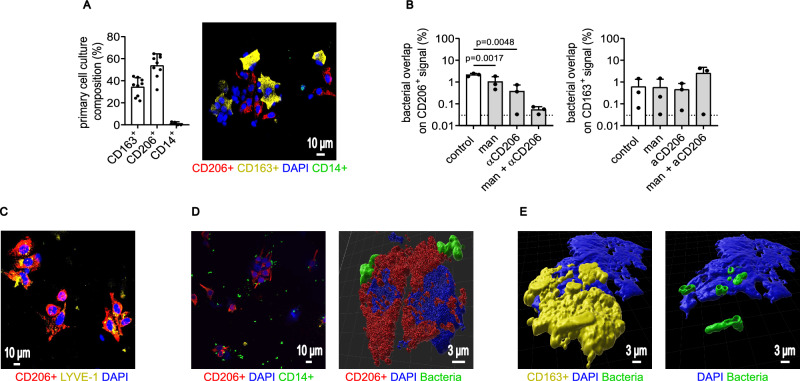
Characterisation of primary human splenic cell populations and CD206-dependent association of pneumococci with sinusoidal lining cells. Graphs show mean and standard deviation (SD). **A** Quantification of the primary splenic cell population after 24 h of culture on collagen-coated wells (left) and characterisation of the primary cell culture by immunofluorescence confocal microscopy (right). Data derive from primary cultures obtained from two independent human spleen biopsies (biological replicates). For each biopsy, 9 confocal microscopy fields were analysed for CD163^+^ RPMs and CD206^+^ sinusoidal lining cells, and 4 fields for CD14+ cells (technical replicates). These data report the percentage of CD163^+^ RPMs, CD206^+^ sinusoidal lining cells and only negligible numbers of potential monocyte-derived CD14^+^ cells per field. CD163^+^ macrophages are shown in yellow, CD206^+^ sinusoidal cells in red and CD163^-^CD206^-^CD14^+^ cells in green. **B** Analysis of pneumococcal association with CD206^+^ sinusoidal cells (left) and CD163^+^ macrophages (right) under control conditions (white bars) and inhibitory conditions (grey bars: 5 mM mannose, anti-CD206 alone and in combination). Each dot corresponds to one independently analysed confocal microscopy field (n = 3). Marker associations were calculated using Fiji (v1.53). Dotted lines indicate the limit of detection (0.03%). **C** Lyve-1 (yellow) expression by CD206^+^ cells (red). Cell nuclei are shown by DAPI staining (blue). **D** 3D confocal reconstruction of the interaction between bacteria (green) and splenic sinusoids (red). **E** 3D confocal reconstruction of the interaction between bacteria (green), splenic sinusoids (red) and CD163^+^ RPMs (yellow), showing intracellular bacteria only in macrophages. Statistical significance was determined by ordinary one-way ANOVA with Tukey’s two-sided post-hoc test (*p* values for all comparisons are shown directly in the figure). Source data are provided as a Source Data file.

**Fig. 5:**
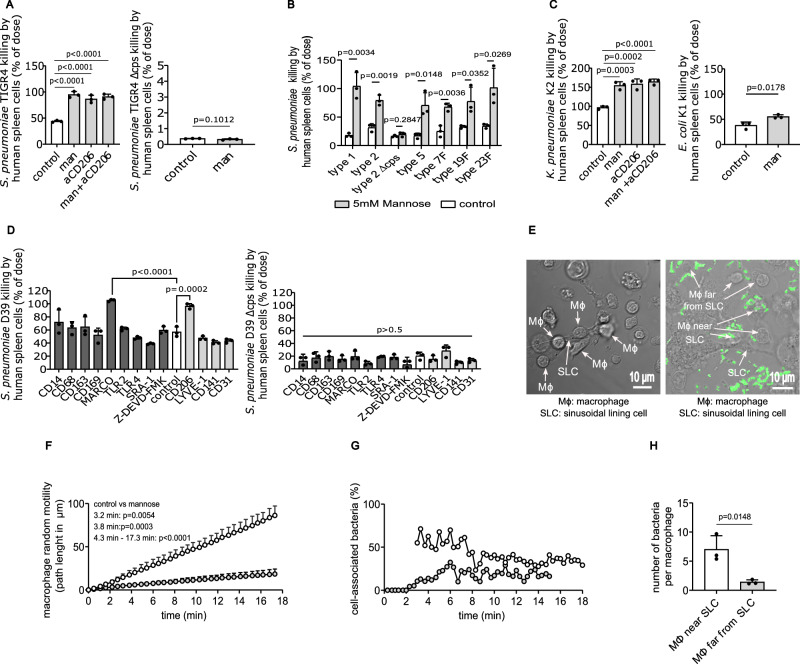
CD206-dependent capture and killing of encapsulated bacteria by human splenic cells revealed by receptor inhibition and time-lapse imaging. Graphs report mean +/- SD. **A** Bacterial survival 30 minutes post-infection for TIGR4 (left) and its non-encapsulated derivative (right), under control (white bars) or CD206 inhibition (5 mM mannose/anti-CD206 antibodies) (grey bars). **B** Bacterial survival of additional encapsulated pneumococcal serotypes linked to disease (1, 2, 5, 7) or carriage (19 F, 23 F), plus a rough derivative of serotype 2 (white bars: control; grey bars: 5 mM mannose). **C** CD206-dependent killing of the *K. pneumoniae* strain GMR15 (left) and the *E. coli* strain UTI-89 (right) (white bars: control; grey bars: inhibition). **D** Receptor blockade on macrophages and sinusoidal cells and its effect on bactericidal activity against the encapsulated pneumococcal strain D39 (left) and its non-capsulated mutant (right). Dark-grey bars: inhibition of macrophage receptors and Z-DEVD-FMK treatment, light-grey bars: inhibition of sinusoidal lining cells, white bars: non-inhibited control conditions. **A**–**D** Experiments were performed in triplicates. Each dot represents one independent experimental replicate of the assay. **E** Confocal time-lapse microscopy of primary splenic cell cultures in the absence (left) or presence (right) of GFP-expressing pneumococci. Time-lapse videos are available at (10.6092/unibo/amsacta/8725) (Supplementary data 2). **F** Analysis of time-lapse imaging showing macrophage random motility over the recording course (white symbols: control; grey symbols: 5 mM mannose). Data represent means of 4 macrophages (control) and 7 macrophages (mannose). Observations derive from primary cultures of the same organ. **G** Image analysis showing the percentage of cell-associated bacteria over the recording course (white symbols: control; grey symbols: 5 mM mannose). **H** Average number of bacteria per macrophage located proximal to sinusoidal cells (white bar) or distal from them (grey bar). Each bar represents the mean of three individual macrophages per group, with each dot corresponding to the average bacterial count for one macrophage across frames. Data derive from technical replicates within a single culture. SNL sinusoidal lining cell; Mɸ macrophages. Statistical significance was determined by ordinary one-way ANOVA with Tukey’s two-sided post-hoc test (**A**–**D**), two-way ANOVA with two-sided Šídák’s multiple comparisons test (**F**) and two-sided unpaired parametric t-test (**H**) (*p* values for all comparisons are shown in the figure). Source data are provided as a Source Data file.

To provide in vitro evidence for the phenotypes observed during ex vivo spleen perfusion, primary adherent cell cultures were established from human spleen homogenates. Primary spleen cultures contained 57% CD206⁺ sinusoidal lining cells and 35% CD163⁺ macrophages, often linked by extensions (Fig. 4A). CD14⁺ cells comprised just 1.4%, indicating rare monocytes. As CD14⁺ monocytes can also express CD206, this suggests that potential overlap with the CD206^+^ population is negligible^30^. Finally, very few CD169^+^ macrophages were also detected (Supplementary Fig. 5D). In MOI 10 infections, 2.1% of bacteria associated with CD206^+^ cells and 0.6% with CD163^+^ macrophages (Fig. 4B). Treatment with mannose or anti-CD206 antibodies reduced bacterial binding to CD206^+^ cells but had no effect on association with CD163^+^ macrophages (Fig. 4B). The expression of CD206 on primary sinusoidal lining cells, and not macrophages, was confirmed by CD206^+^ co-expression with the sinusoidal marker LYVE-1 (Fig. 4C). Three-dimensional reconstructions confirmed that pneumococci remained on the surface of CD206^+^ cells (Fig. 4D), while bacteria appeared intracellular only in macrophages (Fig. 4E). At MOI 10, bacterial counts dropped sharply within 30 min for both TIGR4 (type-4) and D39 (type-2) serotypes (Figs. 5A, B; white bars), but this bactericidal activity was lost when CD206 was blocked by mannose or antibodies (Figs. 5A, 5B; grey bars). The same results were obtained when the cultures were infected with other pneumococcal serotypes, including serotypes more frequently associated with invasive disease (1, 5 and 7) or carriage (19 F and 23 F)^26^ (Fig. 5B). In contrast, when cultures were challenged with a non-encapsulated derivative of the TIGR4 strain (type-4 Δcps), over 99% of bacteria were eliminated within 30 minutes, and this was unaffected by mannose supplementation (Fig. 5A). Comparable data were observed for a rough non-encapsulated D39 strain (type-2 Δcps) (Fig. 5B). Finally, the clearance of an encapsulated pneumococcal pneumolysin mutant in our primary cultures was affected by mannose supplementation and anti-CD206 antibodies, indicating that in this experimental setup, the CD206-mediated capture is dependent on the polysaccharide capsule, despite reports of carbohydrate-independent pneumolysin interaction with the mannose receptor^31,32^ (Supplementary Fig. 3A). These results strongly suggest that CD206-mediated bacterial attachment to sinusoidal lining cells is essential for enabling subsequent macrophage-mediated killing of encapsulated pathogens. The control experiments with the non-encapsulated strains reinforce the conclusion that the CD206-dependent interaction between sinusoidal lining cells and macrophages is not required for the clearance of non-encapsulated bacteria. In addition to pneumococcal capsules, CD206 had been shown to bind also *K. pneumoniae* LPS^13^. Performing the bactericidal assay with capsulated K2 capsule *K. pneumoniae* and K1 capsule *Escherichia coli*, confirmed CD206-dependence for any bactericidal activity of macrophages against these capsulated bacteria rapidly replicating during the bactericidal assay (Fig. 5C). These in vitro results fully confirm the observations during organ perfusion, indicating that CD206-mediated bacterial attachment to sinusoidal lining cells is essential for enabling subsequent macrophage-mediated killing of encapsulated pathogens.

To determine whether receptors other than CD206 contribute to the uptake of encapsulated pneumococci, we repeated the bactericidal assay on primary splenic cell cultures, in the presence of antibodies against a panel of macrophage receptors, as well as the sinusoidal lining cell markers CD31, CD141, and LYVE-1 (Fig. 5D). Phagocytosis of the pneumococcal D39 strain was affected only when CD206 on sinusoidal lining cells or the macrophage scavenger receptor MARCO on macrophages (Macrophage Receptor with Collagenous Structure) were targeted. None of the receptor-specific antibodies tested reduced the efficient killing of non-encapsulated pneumococci, suggesting involvement of other scavenging-receptors not tested in our assay or the involvement of multiple uptake pathways (Fig. 5D). This finding contrasts with murine CD206^+^ primary splenic macrophages, in which inhibition of the CD68 scavenger receptor blocked the killing of encapsulated pneumococci, probably reflecting the restriction of MARCO to a subset of marginal zone macrophages^33^ (Supplementary Fig. 3B).

To obtain insight into the events leading to bacterial clearance, we performed live confocal time-lapse imaging of primary splenic cultures infected with GFP-expressing pneumococci (Fig. 5E, Supplementary data 2, 10.6092/unibo/amsacta/8725), with or without mannose supplementation (Supplementary Fig. 4). Mannose markedly reduced bacteria–cell association to both macrophages and sinusoidal lining cells (Fig. 5G), with macrophages adjacent to sinusoidal lining cells harbouring roughly eight times more bacteria than those located farther away (Fig. 5H). Cell motility analysis over 20 minutes time-lapse showed that macrophage mobility was unaffected by the presence of bacteria (Supplementary Fig. 4A, B, C, 4). Mannose likewise did not alter motility of macrophages distant from sinusoidal lining cells (Supplementary Fig. 4B, D), but surprisingly, macrophages in contact with sinusoidal lining cells became immotile in presence of mannose (Fig. 5F; and Supplementary Fig. 4 A1, 4C). These data suggest for the first time that splenic macrophages activity critically depends on CD206 availability.

These observations support a sequential “handoff” model for encapsulated bacteria clearance in the human spleen: i) CD206^+^ sinusoidal lining cells capture encapsulated bacteria without internalisation and ii) subsequently present them to macrophages, likely for a MARCO-mediated uptake and phagocytosis.

Blocking CD206 prevents this initial capture and reduces downstream bacterial association with macrophages. The same dependency was observed across multiple pneumococcal serotypes (1, 5, 7, 19 F, 23 F) as well as capsulated hypervirulent *K. pneumoniae* and ExPEC *E. coli*, demonstrating that the CD206-mediated mechanism appears to be conserved across diverse encapsulated pathogens, in line with the broad pathogen binding activity of CD206^13,34,35^. Interestingly, non-encapsulated pneumococci were eliminated efficiently, independent of CD206, indicating that capsule-driven glycan recognition is the critical determinant for this pathway. Together, these findings reveal a conserved clearance module in which CD206^+^ sinusoidal lining cells capture capsulated pathogens, enabling subsequent uptake by macrophages. Time-lapse confocal microscopy provided dynamic support for this mechanism. Mannose supplementation sharply reduced the frequency of bacterial–cell contacts, confirming the central role of CD206 in the initial capture step. In addition, macrophages located near sinusoidal lining cells acquired markedly more bacteria than those at a distance, consistent with a directed transfer of bacteria from the CD206⁺ sinusoidal lining cells to macrophages. Surprisingly, mannose rendered macrophages immotile only when they were in direct contact with sinusoidal cells, whereas macrophages distant from sinusoids remained mobile. Because macrophage motility was otherwise unaffected by infection or sinusoidal lining cell contact, these findings indicate that CD206-dependent interactions between sinusoidal cells and macrophages are required for productive bacterial transfer and uptake, and this effect may be independent of the bacteria’s intrinsic ability to bind CD206.

Unlike the traditional view that attributes CD206-mediated clearance to macrophages^9,35^, our data show that in the human spleen, these cells are non-phagocytic stromal elements. Functionally, they behave like biological flypaper, retaining pathogens at the sinusoidal lining cell surface until macrophages, particularly CD163^+^ and CD169^+^ subsets, engage them (Supplementary Fig. 2B, C). The mannose-inhibitable spectrum of this capture extends beyond pneumococcus to multiple encapsulated species, echoing mechanical retention of poorly deformable cells in splenic sinuses^36,37^ but based on biochemical glycan recognition.

The anatomical differences in CD206 distribution between human and mouse spleen, mirror differences in the distribution of ligands for the cysteine-rich (CR) domain of CD206 that recognises sulphated glycans^38,39^. CR-Ligands co-localise with CD206^+^ sinusoidal cells in human spleen but locate to CD206^-^ metallophilic macrophages in mouse spleen^25,38,40,41^. Importantly, the presence CR-Ligands has been linked to receptor-mediated antigen transfer in lymphoid organs^42,43^, and it is plausible that CD206 + , CR-Ligands+ sinusoidal cells could specialise in bloodstream pathogen transfer to resident splenic macrophages. Little is known regarding the biology of CD206 in non-myeloid cells, but in lymphatic endothelial cells, CD206 has been implicated in leucocyte trafficking and tumour metastasis^44^. This aligns with a potential shift from CD206-mediated endocytosis in myeloid cells to CD206-mediated adhesion in endothelial cells and is consistent with the lack of bacterial internalisation by the CD206^+^ sinusoidal lining cells. (Fig. 3C1-C3). Moreover, murine spleens display robust CD206 expression directly on red pulp splenic macrophages^24,25^. In mice, the mannose receptor is predominantly expressed by macrophages and has been shown to facilitate recognition, internalisation and intracellular trafficking of mannose-rich microbial ligands^45–47^. Consistent with this, inhibition of CD206 on primary murine splenic macrophages significantly reduced uptake and impaired killing of encapsulated *S. pneumoniae* (Supplementary Fig. 3B), providing direct functional evidence that CD206 is required for macrophage-mediated elimination of encapsulated pneumococci.

Receptor specificity assays sharpen this model. Inhibition of the macrophages receptor MARCO selectively impaired macrophage clearance of encapsulated pneumococci. Together, these results establish that CD206-mediated capture by sinusoidal lining cells is the obligate entry point for encapsulated bacteria into the macrophage killing pathway, while MARCO is the dominant macrophage receptor acting downstream of CD206. This suggests a receptor-segregated handoff system in which sinusoidal lining cell CD206 acts as the catcher and macrophage MARCO functions as the receiver. Lastly, our findings across multiple pneumococcal serotypes (1, 2, 4, 5, 6 A, 7 F, 19 F, and 23 F) indicate that, in both organ perfusion and primary macrophage assays, differences in killing were minimal. While this does not imply that all serotypes are cleared equally in vivo, it suggests that within the resolution of our experimental systems, macrophage-mediated clearance operates similarly across these tested serotypes. Importantly, these results should not be extrapolated to predict invasive disease potential, as subtle differences may exist that are beyond the detection limits of our assays.

The therapeutic implications of these results are twofold: enhancing CD206-mediated capture could strengthen host defences, while systemic mannose administration, as in some urinary tract infection (UTIs) treatments^48^, could inadvertently impair splenic clearance. Our findings also clarify that in the spleen, CD206 is not a macrophage marker but a functional receptor on sinusoidal lining cells. This corrects earlier assumptions that CD206-mediated phagocytosis in spleen was macrophage-driven^9,49^ repositioning these cells as dedicated antigen-capturing and presenting cells. By capturing encapsulated bacteria via mannose recognition, these sinusoidal lining cells transform a microbial evasion strategy, the anti-phagocytic capsule, into a vulnerability. The concept parallels earlier work showing that erythrocytes with exposed mannoses, including *P. falciparum*–infected cells, are preferentially retained and cleared^16^. Our results extend this to bacteria, demonstrating that the spleen uses mannose recognition to detect and trap both infected cells and encapsulated microbes. In summary, the human spleen is not just a passive filter but an active immune organ combining physical, biochemical, and receptor-mediated mechanisms to eliminate bloodstream threats. Redefining CD206^+^ sinusoidal lining cells as non-phagocytic, but indispensable for microbial capture, offers a new perspective on splenic immunity and opens the door to targeted interventions that reinforce this early line of defence. These findings demonstrate that sinusoidal lining cells start splenic clearance by immobilising encapsulated bacteria, and allowing for their subsequent uptake and killing by macrophages.

### Mechanism of pathogen clearance by both CD163^+^ and of CD169^+^ macrophages

**Fig. 6:**
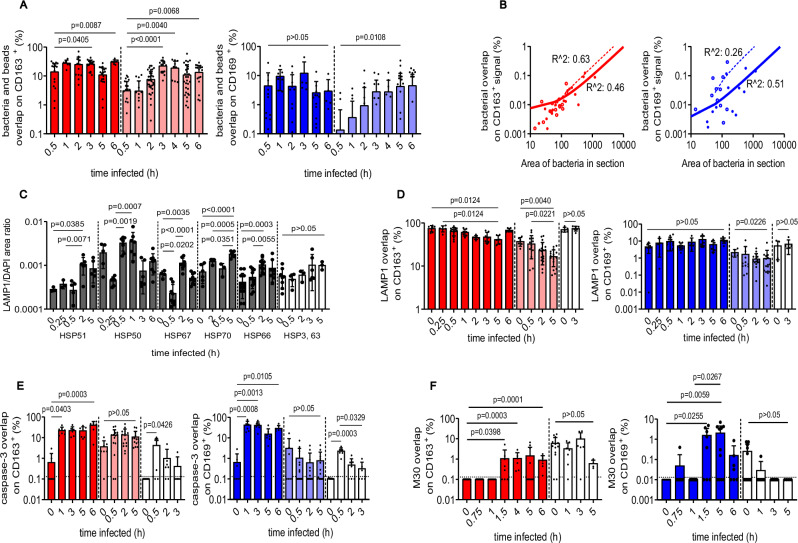
Bacterial uptake and apoptosis of human splenic macrophages during ex vivo perfusion. Graphs show mean +/- SD. **A** Colocalization of pneumococci with CD163⁺ RPMs (left) and CD169⁺ PCSAMs (right) during perfusion. Data derive from *n* = 2 infected and *n* = 3 bead-perfused spleens (biological replicates). Each dot represents one analysed image region (“stamp”). CD163⁺ analysis: 8–23 bacterial stamps and 10–36 bead stamps; CD169⁺ analysis: 5–12 bacterial stamps and 10–30 bead stamps (technical replicates). Association of fluorescent micro-beads with CD163⁺ (light-red) and CD169⁺ (light-blue) macrophages is also shown. **B** Simple linear regression of relationship between bacterial burden and macrophage area occupancy for CD163^+^ (red) and CD169^+^ (blue). Each point represents one analysed biopsy from *n* = 5 independent spleens. Solid-lines and filled-circles indicate D39; dotted-lines and open-circles indicate TIGR4. R² values are shown next to each line. **C** Quantification of LAMP1 signal over time in infected spleens (grey bars) (*n* = 5, 3–12 stamps per time-point) and pooled control spleens (white bars) (*n *= 2, 3–6 stamps). Each dot represents one stamp and bars represent perfusion time points; data are normalised to tissue area. **D** LAMP1 colocalization with CD163⁺ (red bars) and CD169⁺ (blue bars) macrophages in pooled spleens (*n* = 2 infected, *n* = 3 bead-perfused, *n* = 1 control). Each dot represents one analysed stamp. Technical replicates: infected 6–13 stamps, bead-perfused 6–19 stamps, control 5–6 stamps per group. **E** Quantification of cleaved caspase-3 in CD163⁺ (red bars) and CD169⁺ macrophages (blue bars) during perfusion. Data derive from *n* = 1 infected spleen, *n* = 3 pooled bead-perfused spleens, and *n* = 2 control spleens (biological groups). Each dot represents one analysed stamp. Technical replicates: infected 6–7 stamps, bead 7–15 stamps, control 6–8 stamps. **F** M30 levels in CD163⁺ (red bars) and CD169⁺ (blue bars) macrophages during infection. Data derive from *n* = 5 infected spleens and *n* = 2 control spleens (biological groups). Each dot represents one analysed stamp. Technical replicates: infected 6–18 stamps, control 4–13 stamps per group. **E**, **F** Dotted lines indicate detection limits (**E**, **F**: 0.1% and 0.01% as indicated). Time is expressed in hours (h). Significance was determined using non-parametric one-way ANOVA with Kruskal–Wallis post-hoc (Dunn’s multiple comparisons two-sided) for panels **A** and **D**–**F**, and ordinary one-way ANOVA with Tukey’s two-sided post-hoc test (**C**) (*p* values are shown in the figure). Source data are provided as a Source Data file. Spleens analysed are listed in Supplementary Table 5.

The splenic macrophage subsets CD163^+^ RPMs and the less abundant CD169^+^ PCSAMs, operate in distinct, but complementary niches^21^. Quantitative colocalization revealed that 15–30% of bacteria were associated with CD163^+^ macrophages, while 4–22% associated with CD169^+^ macrophages (Fig. 6A). Notably, these proportions were consistent between early (30 min) and late (5 h) time points, despite >90% clearance of bacteria from the perfusate within the first hour. In addition, microscopy analyses of tissue sections with high or low bacterial loads revealed a consistent ratio of bacteria-to-macrophage association across both CD163^+^ and CD169^+^ populations (Fig. 6B), suggesting that even at high challenge doses, macrophage capacity for bacterial clearance was not saturated. The ratios remained consistent across different bacterial loads and serotypes (2, 4, 19 F) (Supplementary Fig 2 A1–A2), indicating stable, load-independent pathogen engagement by these macrophage subsets. When fluorescent beads were perfused instead of live bacteria (Supplementary Fig. 5A–C), macrophage uptake was slower, with association rates increasing progressively over time (Fig. 6A). This slower uptake highlights the dynamic responsiveness of splenic macrophages to live microbes versus inert particles, indicating that bacteria are recognised more efficiently than beads. While these data do not allow quantification of the relative bactericidal contributions of each macrophage subset, they indicate that both CD163^+^ and CD169^+^ macrophages consistently retained similar bacterial loads over time, implying a comparable role in pneumococcal capture.

To investigate the mechanisms of bacterial killing by human spleen tissue-resident macrophages, spleen sections, at different time points of infection (from 0 to 6 h), were stained for the phagosome maturation marker LAMP1. LAMP1 staining, marking lysosomal compartments, revealed a progressive increase in infected spleens, but not in uninfected controls (Fig. 6C). This signal was localised mainly within macrophages (Fig. 6D), consistent with active lysosomal engagement. Overall, the time-dependent increase in LAMP1 levels supports the conclusion that human spleen macrophages remain engaged in active phagocytosis during our ex vivo perfusion model and exhibit dynamic features of the phagocytic process.

Apoptosis has long been recognised as a key mechanism by which human alveolar macrophages eliminate pneumococci^50^. To assess whether this mechanism is also active in human splenic macrophages, tissue sections were stained for cleaved caspase-3, the activated form of the caspase responsible for executing apoptotic proteolysis^51,52^. The infection triggered an early apoptotic response. Within 60 minutes, cleaved caspase-3 levels rose rapidly, over 10-fold in both CD163^+^ RPMs and in CD169^+^ macrophages (Fig. 6E). Initial quantification based on marker-area overlap suggested higher caspase-3 signal within CD169⁺ regions (Supplementary Fig. 3D); however, this approach is inherently influenced by differences in marker distribution and cell density. When the same data were re-analysed using QuPath-assisted whole-cell segmentation to approximate individual macrophages (Supplementary Fig. 3E), caspase-3 activation was equivalent on a per-cell basis in CD163⁺ and CD169⁺ populations, indicating comparable engagement of apoptotic pathways in both subsets. In bead-perfused control spleens, apoptotic markers increased more slowly and never exceeded baseline levels observed at the time-zero biopsy, indicating an absence of apoptosis induction during perfusion. In contrast, infected spleens showed a sustained accumulation of apoptotic markers even at later time points, consistent with infection-driven activation of macrophage cell-death programmes. M30 staining, indicative of caspase-3–cleaved cytokeratin-18^53,54^, increased steadily over 5–6 h in both subsets (Fig. 6F), with the M30-positive macrophage area expanding accordingly (Supplementary Fig. 3G). Finally, to determine whether apoptotic activation scales with bacterial load, we additionally analysed spleen infected with the lower dose of pneumococci (1 × 10^7^ CFU). In this tissue the total cleaved caspase-3 positive area was reduced by approximately an order of magnitude, nearing background signal levels (Supplementary Fig. 3F). This dose-dependent reduction suggests that macrophage apoptosis is coupled to the magnitude of bacterial engagement. Of notice, inhibition of caspase-3 activation, via Z-DEVD-FMK^55^, did not impact bactericidal activity of primary cultures (Fig 5D1, D2).

Finally, considering that a human spleen contains ~2 × 10^10^ macrophages^56^, we found that at the 1-hour mark, 0.065% of CD163^+^ macrophages were positive for cleaved caspase-3 (Supplementary Fig. 3D). Given that caspase-3⁺ areas were roughly ten times smaller than the total CD163⁺ regions, we estimate that ~0.65% of CD163⁺ macrophages were positive for this apoptosis marker. This corresponds to roughly 1 × 10^8^ apoptotic cells per spleen; numbers that closely match the bacterial challenge dose of 1 × 10^8^ CFU. This correspondence suggests a direct link between the quantity of phagocytosed bacteria and the induction of macrophage apoptosis.

The lysosomal activation observed after bacterial uptake, marked by increased LAMP1 expression, confirms that splenic macrophages are competent for canonical phagolysosomal killing^57^. However, the concurrent detection of early cleaved caspase-3 within 60 minutes, followed by M30 expression at 5–6 h, indicates that bacterial clearance in the spleen is closely coupled to programmed cell death. This differs from the delayed apoptosis reported in other macrophage populations, such alveolar macrophages, where cell death typically occurs in the resolution phase of infection^50,58^. Although we did not specifically investigate functional differences between CD163^+^ red pulp macrophages and CD169^+^ macrophages, we observed that the early apoptotic response was engaged to a comparable extent in both subsets, despite their distinct anatomical localisation. While CD169^+^ macrophages have been proposed to play specialised roles in the early control of blood-borne pathogens in murine spleen, including bacterial trapping^18,20^ and immune coordination^59^, our data indicate that, in the human spleen, both macrophage subsets similarly activate lysosomal and apoptotic programmes following bacterial uptake. The spleen’s context may explain this divergence from other tissues. As a blood-filtering organ, it is uniquely exposed to circulating pathogens. In such an environment, rapid apoptosis following phagocytosis could serve as a containment measure, ensuring that intracellular pathogens cannot persist within surviving macrophages. The timing of this response is likely critical for neutralising pathogens capable of manipulating macrophage survival. *Neisseria meningitidis*, for example, can delay apoptosis via nitric oxide detoxification^60^, prolonging its intracellular survival. The early initiation of apoptosis in splenic macrophages could therefore represent an evolved countermeasure against such strategies, an immunological “self-destruct” that denies bacteria a long-term niche.

Spleen ex vivo perfusion, used for malaria^8,16^ and proposed for bacterial studies^20^, has inherent limitations, some linked to reperfusion injury (e.g., high cytokines and soluble proteins) (Supplementary Fig. 6)^61^. However, no differences in cytokine production were observed between mannose-treated and untreated spleens (Supplementary Fig. 6A–D), nor was any difference in complement C3 content in the perfusion liquid between control and infected spleens (Supplementary Fig. 6G). The effects of ischaemia–reperfusion injury were not fully examined, although minimal tissue necrosis and stable lactate levels suggest limited functional compromise. The low number of neutrophils, diluted serum antibodies, and reduced complement levels may also affect bacterial clearance. Cytokine concentrations, while elevated, remained within ranges observed during normothermic liver perfusion^62^.

In conclusion, our findings support a revised model of splenic antibacterial defence in which CD206⁺ sinusoidal lining cells and macrophages act in coordinated sequence. CD206⁺ sinusoidal cells immobilise encapsulated bacteria via mannose-dependent recognition without internalisation, preventing their return to circulation and enabling targeted clearance. CD163⁺ and CD169⁺ macrophages then receive these retained bacteria, phagocytose them through MARCO-mediated recognition, initiate lysosomal degradation, and undergo rapid apoptosis, ensuring efficient destruction while preventing intracellular persistence and systemic re-seeding.

Live confocal time-lapse imaging reveals an additional, previously unappreciated layer of spatial coordination in human splenic clearance. Macrophages in direct contact with sinusoidal lining CD206⁺ cells displayed active behaviour under baseline conditions but became immotile when CD206 was blocked by mannose. In contrast, macrophages not in contact with sinusoidal lining cells maintained their motility regardless of mannose treatment. This context-dependent effect indicates that the immobilisation is not a global consequence of lectin–glycan blockade but requires physical engagement with the sinusoidal lining cell interface.

These observations suggest that CD206 on sinusoidal lining cells acts as a local interface for macrophages engagement, potentially stabilising their spatial orientation and facilitating efficient bacterial handoff. While CD206 is a C-type lectin that binds a large set of glycans on pathogens^13–16,63^ the motility effect in macrophages appears independent of pathogen binding per se and instead reflects the role of CD206 as part of the sinusoidal lining cell microenvironment that coordinates macrophage activity. This aligns with concepts from other lymphoid tissues, where chemokine and adhesion receptor networks dynamically regulate dendritic cell and macrophage behaviour at specialised niches^64,65^. Downstream the function of the mannose receptor, the macrophage’ MARCO scavenger receptor mediates subsequent uptake^66^.

This architecture bears resemblance to antigen-handling systems in lymph nodes, where lymphatic endothelial cells and subcapsular sinus macrophages coordinate pathogen retention and transfer^36^. However, the splenic system is tuned for speed and broad-spectrum efficiency, particularly against encapsulated bacteria that evade complement-mediated lysis and antibody opsonisation. In effect, the capsule, a key virulence factor, becomes a vulnerability when confronted with CD206-mediated capture. The efficiency of this system underscores why loss of splenic function, whether through splenectomy, infarction, or disease-related atrophy^3–5^, carries such a high risk of overwhelming infection (OPSI), particularly from encapsulated organisms^27^. These patients lack both the macrophage-rich red pulp and the specialised sinusoidal lining cells network, severely compromising their capacity to trap and neutralise bloodstream bacteria. Functional hyposplenism, as seen in sickle cell disease or coeliac disease, often involves reduced sinusoidal lining cell density, further increasing vulnerability to severe bacterial infections^67,68^.

Finally, recognising the central role of CD206-mediated capture suggests new interventions such as strategies to preserve/replace macrophages to boost killing capacity. Conversely, free mannose, used to prevent UPEC adhesion^48^, could transiently inhibit splenic CD206, impairing clearance of encapsulated pathogens. This risk may be important in patients with complicated UTI, concurrent bacteraemia, or reduced splenic reserve, warranting clinical caution.

## Methods

### Ethics

Ethical authorisation for a clinical trial of ex vivo normothermic human spleen perfusion (Tissue models of invasive disease TIMID) was obtained from the UK Health Research Authority (HRA REC 18/EM/0057)^20^. The trial was sponsored by the University of Leicester with chief investigator M.R. Oggioni and the study was conducted at the University Hospitals of Leicester NHS Trust (Clinical Trials gov Identifier NCT04620824) with principal investigator A. Dennison. Informed consent was obtained from each study participant, and each spleen was numbered (i.e., HSP1, HSP2, etc.). The organs were transferred to the research lab fully anonymised, and no patient related data are available for any analysis in this study. Two spleen samples for additional cell culture experiments were obtained from a similar clinical trial for ex vivo normothermic liver and spleen perfusion in Italy. The trial authorisation was obtained from the Comitato Etico Area Vasta Emilia Centro della Regione Emilia-Romagna (CE-AVEC) (CE 668/2023/Sper/AOUBo of 19/10/2023). Informed consent was obtained from both study participants, and each spleen was numbered (BHS1 and BHS2). The latter trial is sponsored by the University of Bologna with chief investigator M. Ravaioli and M.R. Oggioni as scientific responsible. Sex and/or gender of participants was not collected or analysed because it was not part of the clinical trial design; samples were fully anonymised and used exclusively for ex vivo tissue analysis and primary cells cultures where sex/gender and age variables were not available.

Murine spleen cells for primary cell cultures were obtained from CD1 mice (n = 3 female 6 weeks old) housed under the project licence 436/2023-PR (protocol 2DBFE.15) to M. Caprini in the animal facility of the Department of Pharmacology and Biotechnology at the University of Bologna.

### Ex vivo human spleen perfusion

Human spleens were obtained from consented patients undergoing splenectomy for left-sided pancreatic oncological conditions at the Hepato-Pancreato-Biliary (HPB) unit, University Hospitals of Leicester NHS Trust^20^. Immediately post-resection, the splenic artery was cannulated and flushed with 1 L of heparinized Soltran preservative solution (Baxter, IL, USA). Spleens were surrounded by Soltran and transported on ice to the research laboratory, with efforts to minimise cold ischaemic time. Upon arrival, spleens were flushed again with 500 mL saline supplemented with 25,000 IU heparin (Sigma, St. Louis, MO, USA) to remove clots, before connection to a custom-built perfusion circuit (Medtronic, Dublin, Ireland). Perfusion was initiated using 500 mL of a haemoglobin-based oxygen carrier, either Hemopure or Oxyglobin (HbO_2_ Therapeutics, USA), or a customised polymerised haemoglobin prepared at the University of Parma (itHBOC) (Supplementary Table 1)^20,69^. The perfusate was supplemented with either human serum or Dulbecco’s Modified Eagle Medium (DMEM, ThermoFisher), and 10 mg/L colistin sulphate salt (Sigma). The temperature was maintained at 37 °C using a water bath, and oxygen was continuously delivered at 1 L/min. Additional infusions included 5% glucose (w/v) at 30 mL/h, saline supplemented with 500 µg Flolan (vasodilator, epoprostenol sodium; GlaxoS- mithKline, Brentford, UK), and saline containing 5000–15,000 IU of heparin (Sigma, St. Louis, MO, USA). Perfusion pressure was maintained at 80 mmHg, and adjustments to glucose levels or oxygenation were made based on hourly monitoring of pH, pO₂, glucose, and lactate using the Epoc Blood Analysis System (Siemens Healthineers). Once the flow rate stabilised (10–15 min), spleens were challenged with *S. pneumoniae* (Supplementary Table 1) diluted in 20 mL of serum-rich liquid obtained from the first wash-through of the organ with anticoagulant. In particular, the six infected spleens analysed for the mannose work described in this paper (HSP45, HSP47, HSP50, HSP51, HSP67 and HSP70) were infected with a mix of five pneumococcal strains of different serotype (type-2 strain D39, type-4 TIGR4, type-5 PMEN19, type-6A SP6-BS73, type-19F G54) (Supplementary Table 1, Supplementary Table 2). The cumulative challenge doses for the five strains, 1 × 10^8^ CFU (high-dose perfusions) and 1 × 10^7^ CFU (low-dose perfusions), were introduced directly into the circuit or into the perfusate reservoir (Supplementary Table 2). Perfusate and tissue biopsies were collected hourly (and for the first hour every 15 minutes) for bacterial enumeration, microscopy, immunofluorescence and ELISAs. Biopsies were embedded in OCT (CellPath, UK), immediately frozen in dry-ice ethanol and stored at -80 °C. Control experiments used perfusate alone or included polystyrene microspheres (Fluoresbrite YG 0.5 µm and BB 1.0 µm). Warm ischemic time ranged from 4–20 minutes, cold ischemic time from 60–150 minutes, and stabilisation time prior to infection from 10–40 minutes. For mannose receptor inhibition studies, 5 mM mannose was added to the perfusate, and in selected cases, a monoclonal antibody against CD206 was included (5 μg/mL; 321102, BioLegend). In these experiments, bacterial challenge occurred 30 minutes after perfusion began with the mannose-supplemented solution.

### Bacterial strains and cultures

In the TIMID trial spleen perfusion experiments (Supplementary Table 1), infections were carried out using *S. pneumoniae*. Initial infections employed serotype 2 strain D39 and serotype 4 strain TIGR4, either individually or in combination^20^. For the detailed analyses presented in this study, spleens were infected with a panel of five *S. pneumoniae* strains representing different serotypes and antibiotic resistance profiles, enabling differentiation by selective plating (Supplementary Table 2). Strains were cultured on brain heart infusion (BHI) agar supplemented with 3% (v/v) defibrinated horse blood (ThermoScientific) or in BHI broth at 37 °C, 5% CO₂ until reaching an optical density of 0.3 at 600 nm (OD_600_). Bacterial mixtures were prepared in 10% glycerol stocks and stored at –80 °C. Colony-forming units (CFU) were verified before use, and separate aliquots were used for each infection experiment.

### Sample preparation and immunofluorescence (IF) staining

Frozen spleen samples embedded in OCT were sectioned at a thickness of 10 µm using a Leica cryostat (Leica Biosystems, Wetzlar, Germany) and mounted onto polylysine-coated microscopy slides (ThermoScientific). Sections were fixed in 4% (v/v) formaldehyde in PBS (Sigma) for 15 minutes at room temperature (RT), followed by washing with phosphate-buffered saline (PBS, Sigma). Permeabilization was performed using 0.1% (v/v) Triton X-100 (Sigma), after which tissues were incubated for 1 h at RT in blocking solution containing 3% (w/v) bovine serum albumin (BSA, Sigma) and 0.05% (v/v) Tween-20 (Sigma) in PBS. Slides were incubated overnight at 4 °C with primary antibodies diluted 1:100 in blocking solution. After two PBS washes, sections were incubated with secondary antibodies (1:500) for 1 h at RT (Supplementary Table 4), followed by two additional PBS washes. Nuclear staining was performed using DAPI (3 μg/mL; ThermoFisher) for 10 minutes, followed by a wash in PBS and a final rinse in deionized water. Slides were mounted using ProLong Gold Antifade Mountant (ThermoScientific, Waltham, MA, USA) and sealed with clear nail polish.

### Scanning and image analysis

Fluorescently labelled spleen tissue sections were imaged using the Vectra Polaris Automated Quantitative Imaging System (Akoya Biosciences, MA, USA). Imaging was performed at 40x magnification (NA = 0.75) in fluorescence mode. The Vectra Polaris, accessed via the Core Biotechnology Services Advanced Imaging Facility at the University of Leicester, was used for automated whole-slide scanning of our human spleen biopsy samples. Exposure times were optimised for each antibody channel. Fluorescence channels corresponded to Alexa Fluor filter equivalents: AF488 = Opal520 (green), AF568 = Opal620 (red), AF647 = Opal690 (magenta), and AF750 = Opal780 (cyan). Image processing began with spectral unmixing in Phenochart (v1.1), after which 6–8 representative image regions (“stamps”) were exported using InForm software (v2.5.1, Akoya Biosciences). All images were saved in.tiff format and analysed quantitatively in Fiji (v1.53)^70^. Regions of interest (ROIs) were defined using the Image > Adjust > Threshold function for each cell population. These ROIs were then applied to the bacterial fluorescence channels to assess bacterial association to macrophages and sinusoidal lining cells through particle analysis. For cell segmentation images were opened and annotated on Qupath (v0.6.0). The cell segmentation was performed on DAPI staining using the built-in algorithm Cell Detection of Qupath (v0.6.0) and considering a cell expansion of 12 µm.

### Human primary splenic cell isolation

Human spleen biopsies for the isolation of primary splenic macrophages and sinusoidal cells were obtained following informed consent, in compliance with ethical regulations. This study was conducted as part of a clinical trial (TIMID) at the University of Leicester (ClinicalTrials.gov Identifier: NCT04620824) and supplemented by a parallel trial (MOSIE) (ID: CE 668/2023/Sper/AOUBo) coordinated by the University of Bologna. Primary splenic cells were isolated using a modified version of a previously published protocol^71^. Upon collection, spleen biopsies were placed in cold high-glucose Dulbecco’s Modified Eagle Medium (DMEM; Merck). The tissue was roughly chopped and washed several times with cold PBS (Merck) to remove residual blood. Spleen fragments were then injected with 1 mL of an enzymatic digestion cocktail composed of 60% DMEM, 30% Collagenase D (10 mg/mL; Merck), and 10% DNase I (10 mg/mL; Merck), followed by incubation at 37 °C for 15 min. After this initial digestion, the tissue was minced with scissors and transferred to 10 mL of the same enzymatic solution for an additional 15 minute incubation. Following enzymatic digestion, the tissue suspension was passed through a 100 µm strainer (Merck) into a 50 mL tube containing a stop solution (80% DMEM and 20% foetal bovine serum, FBS; Euroclone). The cell suspension was centrifuged at 200 × *g* for 10 min at 4 °C, and the resulting pellet was resuspended in 1 mL of 1X Red Blood Cell (RBC) Lysis Buffer (ThermoFisher). After a 5 min incubation on ice, the solution was diluted with 9 mL of cold PBS and centrifuged again at 200 × *g* for 5 minutes at 4 °C. This RBC lysis step was repeated until no visible red blood cell pellet remained. The final cell pellet was resuspended in complete culture medium consisting of DMEM/F12 (Merck) supplemented with 10% (v/v) FBS, 2% (v/v) penicillin-streptomycin (1X; Fisher Scientific), and 1% (v/v) L-glutamine (200 mM; Fisher Scientific). Cells were then counted, seeded onto collagen-coated wells (PureCol Type I Bovine Collagen solution, Advanced BioMatrix) and maintained overnight for downstream infection assays and microscopy. The same protocol was applied for the isolation of murine primary splenic macrophages.

### In vitro phagocytosis assay

Freshly isolated primary human splenic cells were seeded immediately after isolation onto collagen-coated surfaces at densities of 2 × 10⁵ cells per well in 96-well plates and 1x10⁶ cells per well in 8-well chamber-slides (Ibidi), coated with PureCol Type I Bovine Collagen solution (Advanced BioMatrix). Cells were incubated overnight at 37 °C with 5% CO₂ to allow adherence. The following day, cells were washed with PBS and infected at a multiplicity of infection (MOI) of 10 with the following bacterial strains: *S. pneumoniae* D39 (serotype 2) and TIGR4 (serotype 4); *K. pneumoniae* GMR151 (ST25, K2); and *E. coli* UTI89 (K1) (Supplementary Table 2). Infections were performed at 37 °C with 5% CO₂ for 30 minutes, followed by a 5 min incubation on ice to halt further infection and phagocytosis. After infection, 10 μL of the supernatant was collected, serially diluted, and plated on BHI agar to quantify extracellular bacteria. Cells were then washed once with PBS and lysed using 0.1% (w/v) saponin (Merck) to release both adherent and intracellular bacteria. Lysates were serially diluted and plated on BHI agar for CFU enumeration. For *S. pneumoniae*, BHI agar plates were supplemented with 3% (v/v) defibrinated blood. For immunohistochemistry (IHC), infected chamber slides were fixed in 4% (v/v) formaldehyde (Fisher Scientific) for 15 minutes immediately post-infection and subsequently prepared for microscopic analysis. For CD206 inhibition, cells were pre-treated in DMEM supplemented with 5 mM mannose, 5 µg/mL of anti-CD206 primary antibody, or a combination of both. The inhibition was carried out for 30 minutes at 37 °C in 5% CO₂. Receptor blockade of human and murine primary splenic macrophages was performed as the CD206 inhibition. Following incubation, cells were washed with PBS to remove the inhibition medium and subsequently infected with bacteria as previously described.

### Primary cells immunohistochemistry

Primary human splenic cells seeded on collagen-coated chamber slides were fixed with 4% (v/v) formaldehyde (Fisher Scientific) in PBS (Sigma) for 15 minutes at room temperature (RT), either the day after isolation or immediately following bacterial challenge. Following fixation, cells were washed with phosphate-buffered saline (PBS; Sigma). Permeabilization was carried out using 0.1% (v/v) Triton X-100 (Sigma) in PBS for 10 minutes. Cells were then incubated for 1 h at RT in a blocking solution composed of 5% (w/v) bovine serum albumin (BSA; Sigma) in PBS. Subsequently, cells were incubated with primary antibodies (1:100 dilution in blocking solution) for 1 h at RT in the dark. After two washes with PBS, cells were incubated with fluorophore-conjugated secondary antibodies (1:500) for 1 h at RT in the dark (Supplementary Table 4). Nuclei were counterstained using DAPI (3 μg/mL; ThermoFisher) for 10 minutes. Finally, slides were washed once with PBS and rinsed in deionized water prior to imaging.

### Confocal microscopy imaging

Confocal images of spleen tissue and primary cells were acquired using an Olympus FV1000 confocal laser scanning microscope with 40x (UPlanFLN 40x/NA = 1.3) and 60x (UPlan-SAPO 60x/NA = 1.35) objectives. Image processing was performed using Fiji (v2.0.0-rc69/1.52p). For 3D visualisation, multi-plane Z-stack images were deconvolved using Huygens Essential deconvolution software (v18.04.1p0 64-bit; SVI, Hilversum, Netherlands) and reconstructed using Imaris 3D rendering software. Image and colocalization analysis between cellular markers and bacteria were performed using Fiji (ImageJ)^70^, following the same methodology previously described for tissue sections. Time-lapse confocal imaging of primary splenic cells was performed using a Nikon A1R+ confocal laser scanning microscope with resonant scanning and a 60× objective at the University of Bologna microscopy facility. Cells were seeded at 1 × 10^5^ per well on black, glass-bottom 96-well plates (Corning) and infected with a GFP-expressing *S. pneumoniae* strain at an MOI of 10. Imaging was conducted at 37 °C for ~20 min, acquiring frames every 20 seconds. Image processing and cell tracking were performed in Fiji^3^ using the MTrackJ plugin (Supplementary data 2, 10.6092/unibo/amsacta/8725).

### Human spleen perfusion liquid ELISAs

Human spleen perfusates were analysed for the presence of pro-inflammatory and anti-inflammatory cytokines using Human DuoSet ELISA kits (R&D Systems) following the manufacturer’s recommendations. Assay ranges are TNF-α: 15.6 – 1000 pg/mL, IL-1β: 3.9 – 250 pg/mL, IL-10: 31.2 – 2000 pg/mL, and IL-6: 9.4 – 600 pg/mL. Anti-pneumococcal antibodies in human spleen homogenates were tested against whole bacterial cells (type-2 D39, type 4-TIGR4) and, in perfusion liquid, against pneumococcal polysaccharides (types-2, -4, -19F; SSI Diagnostica). Wells were coated overnight at 4 °C with either 100 μL of 0.2 OD_600_ bacterial suspension in PBS or CPS (1 μg/mL in PBS/0.02% NaN₃). Plates were washed (PBS/0.1% Tween-20), blocked with 5% BSA/0.1% Tween-20 for 1 h, and samples (1:4 in blocking buffer, duplicates) were added, alongside positive control sera (Statens Serum Institute). After overnight incubation at 4 °C, plates were washed and incubated 1 h at RT with anti-human HRP-IgG (1:1000) for samples/negatives or anti-rabbit HRP-IgG (1:5000) for positives. Detection used TMB (Sigma-Aldrich) substrate for 30 min in the dark at RT, stopped with 2 N H₂SO₄, and read at 450/630 nm (FLUOstar Omega). Cut-off values were the mean OD_450_ of negative controls per dilution; samples above the cut-off were considered positive, titres (Supplementary Table 3) were the reciprocal of the last positive dilution.

### Statistical analysis

Statistical analysis was made by non-parametric one-way ANOVA using Kruskal-Wallis test with two-sided Dunn’s multiple comparisons and ordinary one-way ANOVA with Tukey’s two-sided post-hoc test. In addition, two-sided unpaired parametric t-test was used to compare two sets of values. Linear association dynamics between bacterial burden and macrophage area occupation (Fig 6B1-6B2) where determined by simple linear regression lines of best fit for RPMs (B1, red) and PCSAMs (B2, blue). The R^2^ value for each line is written adjacent to the appropriate line on each graph. XY graphs were instead analysed using two-way ANOVA with two-sided Šídák’s multiple comparisons test. The means and standard deviation were calculated by GraphPad, and the significant *p* values (ns *p* > 0.05, * *p* ≤ 0.05, ** *p* ≤ 0.01, *** *p* ≤ 0.001, and **** *p* ≤ 0.0001) were determined.

### Reporting summary

Further information on research design is available in the Nature Portfolio Reporting Summary linked to this article.

## Supplementary information

Supplementary Information

Description of Additional Supplementary Files

Supplementary Data 1

Supplementary Data 2

Reporting Summary

Transparent Peer Review file

## Source data

Source Data

## Data Availability

All original data and the original image files detailed in Supplementary Table 5 (List of spleen perfusion samples used to generate figures) are available through the institutional permanent open access repository AMS Acta of the University of Bologna (https://amsacta.unibo.it/). The time-lapse files are available at 10.6092/unibo/amsacta/8725 (Supplementary data 2) and the raw image and numerical source data are available at 10.6092/unibo/amsacta/8855 (Supplementary data 1). Source data are provided with this paper.
