# Supplementary material for: The mannose receptor on sinusoidal lining cells mediates two-step bacterial clearance in the human spleen
Source: Nat Commun. 2026 Apr 29;17:7595. doi: 10.1038/s41467-026-72430-8 (PMC13421466; doi:10.1038/s41467-026-72430-8)
Supplement: Supplementary file 1 — Supplementary Information [file 41467_2026_72430_MOESM1_ESM.pdf]

Alnabati, Flandi et al.,

The Mannose Receptor on Sinusoidal Lining Cells Mediates Two-Step Bacterial Clearance in the Human Spleen.

SUPPLEMENTARY MATERIALS

**Supplementary Table 1**	**2**
**Supplementary Table 2**	**2**
**Supplementary Table 3**	**3**
**Supplementary Table 4**	**4**
**Supplementary Table 5**	**5**
**Supplementary Figure 1**	**6**
**Supplementary Figure 2**	**7**
**Supplementary Figure 3**	**8**
**Supplementary Figure 4**	**9**
**Supplementary Figure 5**	**10**
**Supplementary Figure 6**	**11**
**References**	**12**

Supplementary tables

Supplementary Table 1: **All *ex vivo* spleen perfusions performed under the TIMID trial**

Number	Date	Weight (g)	CI time (min)	WI time (min)	Perfusate	Challenge	Dose	Comment	Reference
HSP1	08/09/2018				Hemopure	D39	10^8 CFU		1
HSP2	28/08/2018	254			Hemopure	D39	10^7 CFU		1
HSP3	16/10/2018				Hemopure	Control			1
HSP4	12/04/2018				Hemopure	Control			1
HSP5	26/02/2019				Hemopure	TIGR4	10^7 CFU		1
HSP6	04/02/2019				Hemopure	D39-TIGR4	10^7 CFU each		1
HSP7	16/04/2019	312			Hemopure	D39-TIGR4	10^7 CFU each		1
HSP8	13/08/2019				Hemopure	TIGR4	10^7 CFU		1
HSP12	08/07/2020	150			Hemopure	D39-TIGR4	10^7 CFU each		1
HSP24	12/02/2022				Hemopure	D39-TIGR4	10^7 CFU each	α-type 4	1
HSP45	17/08/2022	214			Oxyglobin	MIX1*	10^7 CFU		This work
HSP46	25/08/2022	151			Oxyglobin	D39-TIGR4	10^7 CFU each	α-type 2	
HSP47/9	28/10/2022	115			Oxyglobin	MIX1	10^7 CFU		This work
HSP50	11/11/2022	218			Oxyglobin	MIX1	10^8 CFU		This work
HSP51	02/02/2023	143			Oxyglobin	MIX1	10^8 CFU		This work
HSP55	01/04/2023	130	120	10	ITHBOC	microbeads			This work
HSP56	12/04/2023	211	120	15	Oxyglobin	microbeads			This work
HSP61	23/08/2023	311	110	20	Oxyglobin	microbeads			This work
HSP62	08/09/2023	201	150	20	ITHBOC	*S. aureus, K. pneumoniae*	10^7 CFU each		
HSP63	21/09/2023	152	155	20	ITHBOC	control			This work
HSP64	14/11/2023	189	85	2	ITHBOC	*S. aureus, K. pneumoniae*	10^7 CFU each	20mg/L Gentamicin	
HSP65	13/10/2023	119	106	8	ITHBOC	Poly A tail D39	10^7 CFU	9h total	
HSP66	31/10/2023	92	85	10	Oxyglobin	MIX1 + mAb + Mannose	10^8 CFU	Mannose mAb, 9h total	
HSP67	22/11/2023	121	113	15	ITHBOC	MIX1 + Mannose	10^8 CFU	Mannose	This work
HSP70	03/04/2024	130	133	15	ITHBOC	MIX1 + Mannose	10^7 CFU	Mannose	This work
HSP71	05/06/2024	131	166	20	ITHBOC	Mix1	10^8 CFU	Infect at 6h, 12h total	This work
HSP72	13/06/2024	90	121	30	ITHBOC	Mix1	10^8 CFU	Infect at 6h, 12h total	This work
HSP74	07/08/2024	134	115	15	ITHBOC	Mix1	10^8 CFU	Infect at 6h, 12h total	This work

* MIX1 type-2 D39, type-4 TIGR4, type-5 PMEN19, type-6A SP6-BS73, type-19F G54. CI cold ischemic time, Wi warm ischemic time

Supplementary Table 2: **Strains**

Species	Strain	Serotype	Sequence type (ST)	Antibiotic resistance gene(s)	Reference
S. pneumoniae	PN2189	1	nd*	*tetM*	
S. pneumoniae	D39	2	595	*rpsL**	3
S. pneumoniae	TIGR4	4	205	*zmpC::aad9*	2
S. pneumoniae	PMEN19	5	289	*cat, tetM*	4
S. pneumoniae	PN946	5	nd*		
S. pneumoniae	SP6-BS73	6A	460	*mefA, dfr, pbp2x**	6
S. pneumoniae	PN1456	7F	nd*	*cat*	
S. pneumoniae	G54	19F	63	*tetM, ermB*	5
S. pneumoniae	PN1528	19F	nd*	*ermB*	
S. pneumoniae	PN996	23F	nd*	*pbp2x**	
K. pneumoniae	GMR151	K2	25	SHV-11	7
E. coli	UTI89	K1	95	-	8

*nd not determined

Supplementary Table 3: **Antibody titres**

Perfusion	Antibody titres to whole bacteria in tissue homogenates		Capsule specific antibody titres in the perfusion liquid		
	type-2	type-4	type-2 CPS*	type-4 CPS	type-19F CPS
HSP1**	64	64	32	16	256
HSP2	128	64	neg	16	neg
HSP3	512	256	32	64	256
HSP4	512	512	64	16	256
HSP5	512	128	128	256	neg
HSP6	256	256	neg	neg	neg
HSP7	4096	8192	128	256	neg
HSP15	256	128	64	-	-
HSP45	256	128	64	-	-
HSP46	-	-	8	256	4
HSP49	-	-	neg	neg	neg
HSP50	-	-	8	64	neg
HSP51	-	-	neg	neg	neg
HSP55-74	-	-	-	-	-

* CPS capsular polysaccharide, ** HSP human spleen perfusion

Supplementary Table 4: **primary and secondary antibodies**

**Antibody**	**Specificity**	**Target**	**Host**	**Conjugated**	**Clone**	**Working conc.**	**Catalogue**	**Supplier**
**Primary antibodies**								
type serum 2	*Streptococcus pneumoniae*	Type 2 capsule	Rabbit	-	-	1:500	16745	Statents Serum Institut
type serum 4	*S. pneumoniae*	Type 4 capsule	Rabbit	-	-	1:500	16747	Statents Serum Institut
type serum 5	*S. pneumoniae*	Type 5 capsule	Rabbit	-	-	1:500	16748	Statents Serum Institut
group serum 6	*S. pneumoniae*	Type 6A, 6B, 6C capsules	Rabbit	-	-	1:500	16900	Statents Serum Institut
group serum 19	*S. pneumoniae*	Type 19F, 19A, 19B, 19C capsules	Rabbit	-	-	1:500	16911	Statents Serum Institut
Omni Serum	*S. pneumoniae*	All 91 serotype capsules	Rabbit	-	-	1:500	2438	Statents Serum Institut
Anti-K2 serum	*Klebsiella pneumoniae*	K2 capsule	Rabbit	-	-	1:500	-	Statents Serum Institut
Anti-CD163	Human	CD163	Mouse	-	EDHu-1	1:100	NB110-40686	Novus Biologicals
CD163 Antibody, anti-human, REAfinity	Human, Monkey	CD163	Human Recombinat	Alkaline phosphatase	REA812	1:100	130-112-129	Miltenyi Biotec
Human Siglec-1/CD169 Antibody	Human	CD169	Sheep	-	-	1:100	AF5197	R&D Systems
anti-human CD68	Human	CD68	Mouse	-	PG-M1	1:100	M087601-2	Dako
CD14 antibody	Human	CD14	Goat	-	-	1:100	AHP1059	Bio-Rad
MARCO Monoclonal Antibody (PLK1)	Human	MARCO	Mouse	-	PLK1	1:100	MA5-51863	ThermoFisher
TLR2 Polyclonal Antibody	Human, Mouse	TLR2	Goat	-	-	1:100	PA1-21611	ThermoFisher
TLR4 Polyclonal Antibody	Human, Mouse	TLR4	Goat	-	-	1:100	PA5-142481	ThermoFisher
Human SR-AI/MSR1 Antibody	Human	SR-A1	Mouse	-	-	1:100	MAB27081	Biotechne
anti-human CD206 (MMR) Antibody	Human	CD206	Mouse	-	15-2	1:100	321102	BioLegend
Human MMR/CD206 Antibody	Human	CD206	Mouse	AlexaFluor 750	685641	1:100	FAB25342S-100UG	R&D Systems
Human CD31/PECAM-1 Antibody	Human	CD31	Sheep	-	-	1:100	AF806	R&D Systems
Human LYVE-1 Antibody, Novus Biologicals	Human	LYVE-1	Mouse	AlexaFluor 488	1072614	1:100	30130429	FisherScientific
THBD Mouse anti-Human, Clone: THBD/1782, Abnova™	Human	CD141/THBD	Mouse	-	THBD/1782	1:100	16063256	FisherScientific
Cleaved Caspase 3 p17 Monoclonal Antibody	Human, mouse, rat	Cleaved caspase-3	Mouse	-	2F7B8	1:100	68773-1-IG	ThermoFisher
Caspase-3 Antibody	Human, mouse, rat, pig, chicken, hamster	Pro and active caspase-3	Mouse	AlexaFluor 488	31A1067	1:100	NB100-56708AF488	Novus Biologicals
Anti-LAMP1 antibody	Human	LAMP-1	Rat	-	1D4B	1:100	ab25245	Abcam
**Secondary antibodies**								
Donkey anti-rabbit IgG (H+L), Alexa Fluor 488	Rabbit	Rabbit IgG	Donkey	AlexaFluor 488	-	-	A-21206	Invitrogen
Donkey anti-Mouse IgG (H+L), Secondary Antibody, Alexa Fluor 488	Mouse	Mouse IgG	Donkey	AlexaFluor 466	-	-	A-21202	Invitrogen
Donkey anti-sheep IgG (H+L), Alexa Fluor 568	Sheep	Sheep IgG	Donkey	AlexaFluor 568	-	-	A-21099	Invitrogen
Goat anti-Mouse IgG (H+L), Alexa Fluor 568	Mouse	Mouse IgG	Goat	AlexaFluor 568	-	-	A-11004	Invitrogen
Donkey anti-Rat IgG (H+L), Alexa Fluor 568	Rat	Rat IgG	Donkey	AlexaFluor 568	-	-	A-78946	Invitrogen
Donkey anti-sheep IgG (H+L), Alexa Fluor 647	Sheep	Sheep IgG	Donkey	AlexaFluor 647	-	-	A-21448	Invitrogen
Donkey anti-Mouse IgG (H+L), Alexa Fluor 647	Mouse	Mouse IgG	Donkey	AlexaFluor 647	-	-	A-31571	Invitrogen
Goat anti-Rat IgG (H+L), Alexa Fluor 647	Rat	Rat IgG	Goat	AlexaFluor 647	-	-	A-21247	Invitrogen
Goat anti-Rabbit IgG (H+L), Secondary Antibody, Alexa Fluor 750	Rabbit	Rabbit IgG	Goat	AlexaFluor 750	-	-	A-21039	Invitrogen
Goat anti-Mouse IgG (H+L), Antibody, Alexa Fluor 750	Mouse	Mouse IgG	Goat	AlexaFluor 750	-	-	A-21037	Invitrogen

Supplementary Table 5: **List of spleen perfusion samples used to generate figures**

Figure	Human spleen (HSP)
Figure 1 A	HSP63
Figure 1 B	HSP63
Figure 1 C	HSP51, 50, 45, 62
Figure 1 D-F	HSP1, 2, 3, 6, 7, 8, 12, 15, 24, 50, 51, 54, 55, 56, 63, 66, 67, 70, 71
Figure 2 A	HSP50
Figure 2 B	HSP51
Figure 2 C	HSP67
Figure 2 D	HSP45
Figure 2 E	HSP49
Figure 2 F	HSP70
Figure 3 A	HSP50, 51 (no mannose); HSP66, 67 (mannose)
Figure 3 B	HSP66 (left); HSP51 (right)
Figure 3 C	HSP66
Figure 3 D	HSP50, 51, 24 (no mannose); HSP66, 67 (mannose)
Figure 3 E	HSP50, 51, 24 (no mannose); HSP66, 70 (mannose)
Figure 6 A	HSP54, 55, 56 (light-red and light-blue bars); HSP50, 51 (red and blue bars)
Figure 6 B	HSP1, 2, 6, 7, 8
Figure 6 C	HSP 50, 51, 66, 67, 70 (black bars); HSP3, HSP63 (white bars)
Figure 6 D	HSP3 (white bars); 50, 51 (red and blue bars); 54, 55, 56 (light-red and light-blue bars)
Figure 6 E	HSP3, 63 (white bars); 50 (red and blue bars); 54, 55, 56 (light-red and light-blue bars).
Figure 6 F	HSP3, 71 (white bars); 51, 66, 67, 70, 71 (red and blue bars)
Supplementary Figure 1 A-B	HSP66, HSP55
Supplementary Figure 1 C-D	HSP70
Supplementary Figure 1 E	HSP50, 51, 67
Supplementary Figure 1 F-G	HSP3, 50, 51, 63, 66, 67
Supplementary Figure 2 A	HSP50, 51, 66, 67
Supplementary Figure 2 B-C	HSP67
Supplementary Figure 3 D	HSP3, 63 (white bars); 50 (red and blue bars); 54, 55, 56 (light-red and light-blue bars).
Supplementary Figure 3 E	HSP50
Supplementary Figure 3 F	HSP50 (white bars); HSP45 (grey bars)
Supplementary Figure 3 G	HSP3, 71 (white bars); 51, 66, 67, 70, 71 (red and blue bars)
Supplementary Figure 5	HSP54
Supplementary Figure 6 A-D	HSP2, 3, 4, 5, 6, 7, 12, 15, 24, 45, 46, 49, 50, 51, 57, 62, 63, 64, 65, 66, 67, 70, 71, 72, 74
Supplementary Figure 6 E-F	HSP3, 4, 50, 51, 66, 67, 70, 71, 72, 74
Supplementary Figure 6 G	HSP3, 4, 67, 70

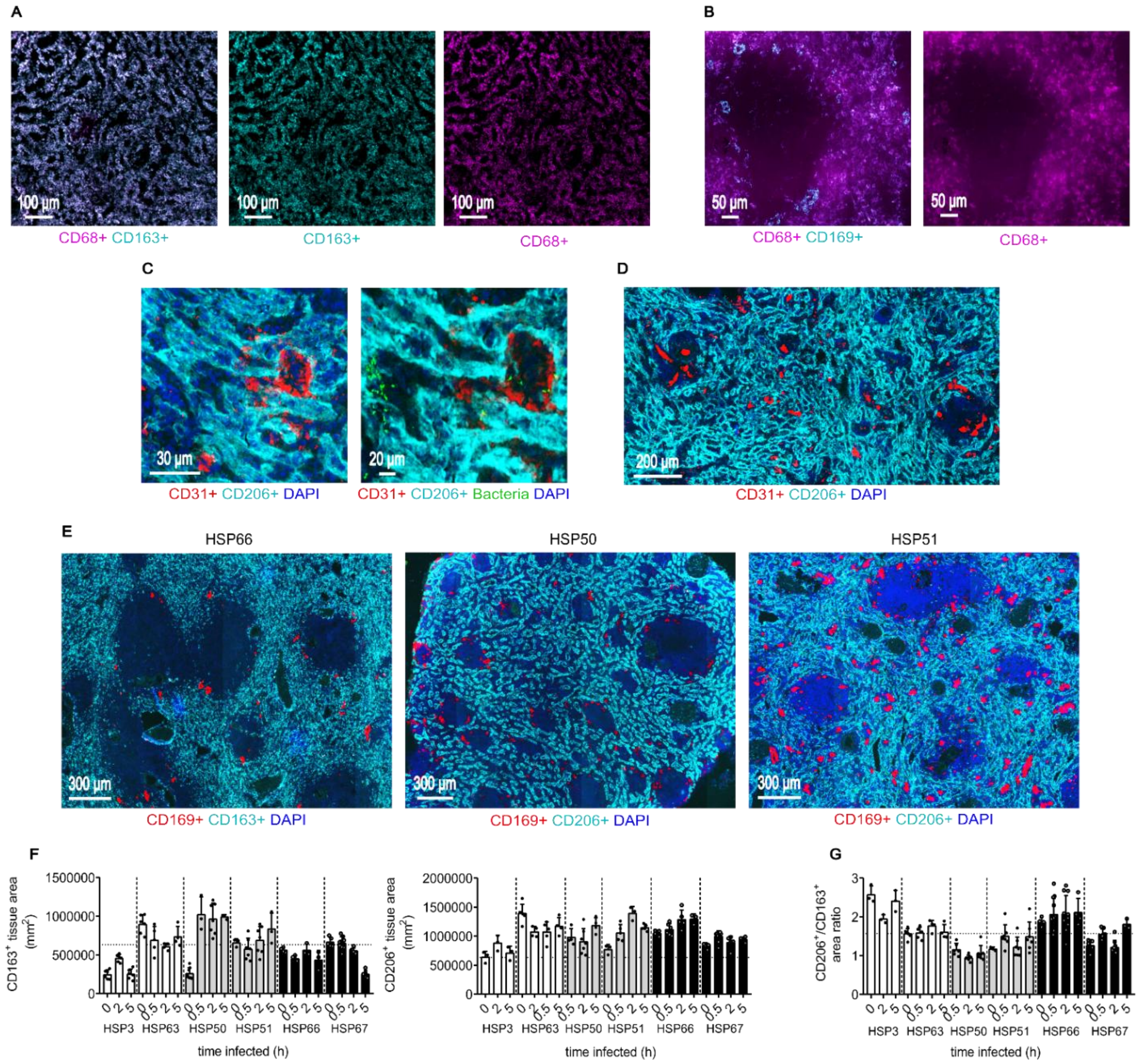

Supplementary Figure 1: Cellular marker expression in the human spleen. Graphs report mean +/- SD. **A** High-content scanning microscopy of a human spleen section showing the colocalization between the CD68 (magenta) and CD163 (cyan) macrophage markers. **B** Scanning microscopy images showing the overlap between the CD68 (magenta) and CD169 (cyan) signals. **C** Whole-section scanning microscopy of human spleen tissue showing $CD31^+$ endothelial cells in red and $CD206^+$ sinusoidal lining cells in cyan and preferential localization of bacteria (green) on the sinusoidal cells rather than on $CD31^+$ cells. Nuclei are shown in DAPI staining (blue). **D** Low magnification images showing distinct distribution of CD206 (cyan) and CD31 (red) markers in the human spleen. Images (panels C–D) are representative of two independent staining experiments and two independent biopsies of the same human spleen (HSP70), with similar results in all cases. **E** Inter-individual variability distribution of the CD169 marker between spleens (HSP66; HSP50; HSP51). Cyan: $CD163^+$ RPMs (HSP66) or sinusoidal lining cells $CD206^+$ (HSP50–HSP51); Red: $CD169^+$ PCSAMs. Cell nuclei are shown in DAPI staining (blue). **F** Quantification of marker-positive area in µm² occupied by the $CD163^+$ RPMs (right) and $CD206^+$ sinusoidal lining cells (left) during the entire duration of the perfusion. **G** Relative ratio in tissue area (µm²) between the $CD206^+$ sinusoidal cells and the $CD163^+$ macrophages. (F–G) White bars indicate control human spleens (HSP3, HSP63), grey bars represent infected spleen (HSP50, HSP51) and black bars represent spleen with mannose supplementation before infection (HSP66, HSP67). Each dot represents an individual analysed image region ("stamp"). The number of stamps analysed per bar ranged from n=3–9 depending on biopsies size. Time is expressed in hour (h). Dotted lines indicate the overall average area, calculated between the entire dataset of human spleens, covered by each marker in tissues. Source data are provided as a Source Data file. Spleens analysed in these panels are listed in Supplementary Table 5.

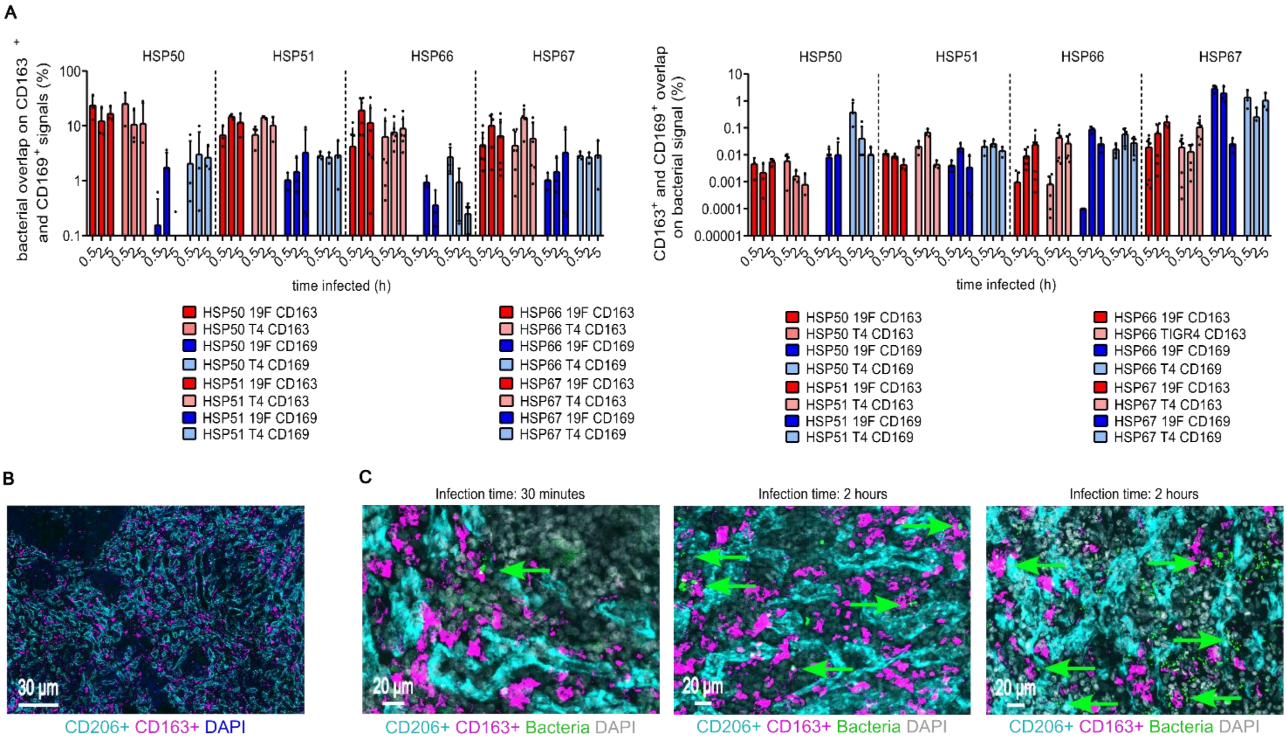

Supplementary Figure 2: **Bacterial distribution in the human spleen.** Graphs report mean +/- SD. **A** Association of 2 different pneumococcal serotypes (right) (TIGR4 (T4): type-4, and 19F: type-19) with $CD163^+$ RPMs (red bars) and $CD169^+$ PCSAMs (blue bars) in four different human spleen (HSP50, HSP51, HSP66, HSP67) throughout infection (hour). Quantification of bacteria-positive macrophage area in $CD163^+$ (red bars) and $CD169^+$ (blue bars) populations (left). Each dot represents an individual analysed image region ("stamp") (technical replicates). The number of stamps analysed per bar ranged from n=3–6, depending on biopsies size. Data derive from independent analyses of all four spleens with similar results (biological replicates). **B**, **C** Splenic sinuses draining the open circulation of the human splenic red pulp are lined by sinusoidal lining cells ($CD206^+$). **B** Bacteria reach the open circulation of the splenic red pulp from the bloodstream. **C** Images from spleens collected at 30 min, 2h and 5h after challenge show bacteria in the red pulp associated to sinusoidal cells (cyan) or macrophages (magenta), but not in the draining sinuses (CD206: cyan; CD163: magenta; CD169: red; DAPI: blue and grey). Images (panels B and C) are representative of three independent spleens (HSP66, 67, 70) (biological replicates) and they summarize the general situations found within the time points of infection in each field of view. Source data are provided as a Source Data file. Spleens analysed in these panels are listed in Supplementary Table 5.

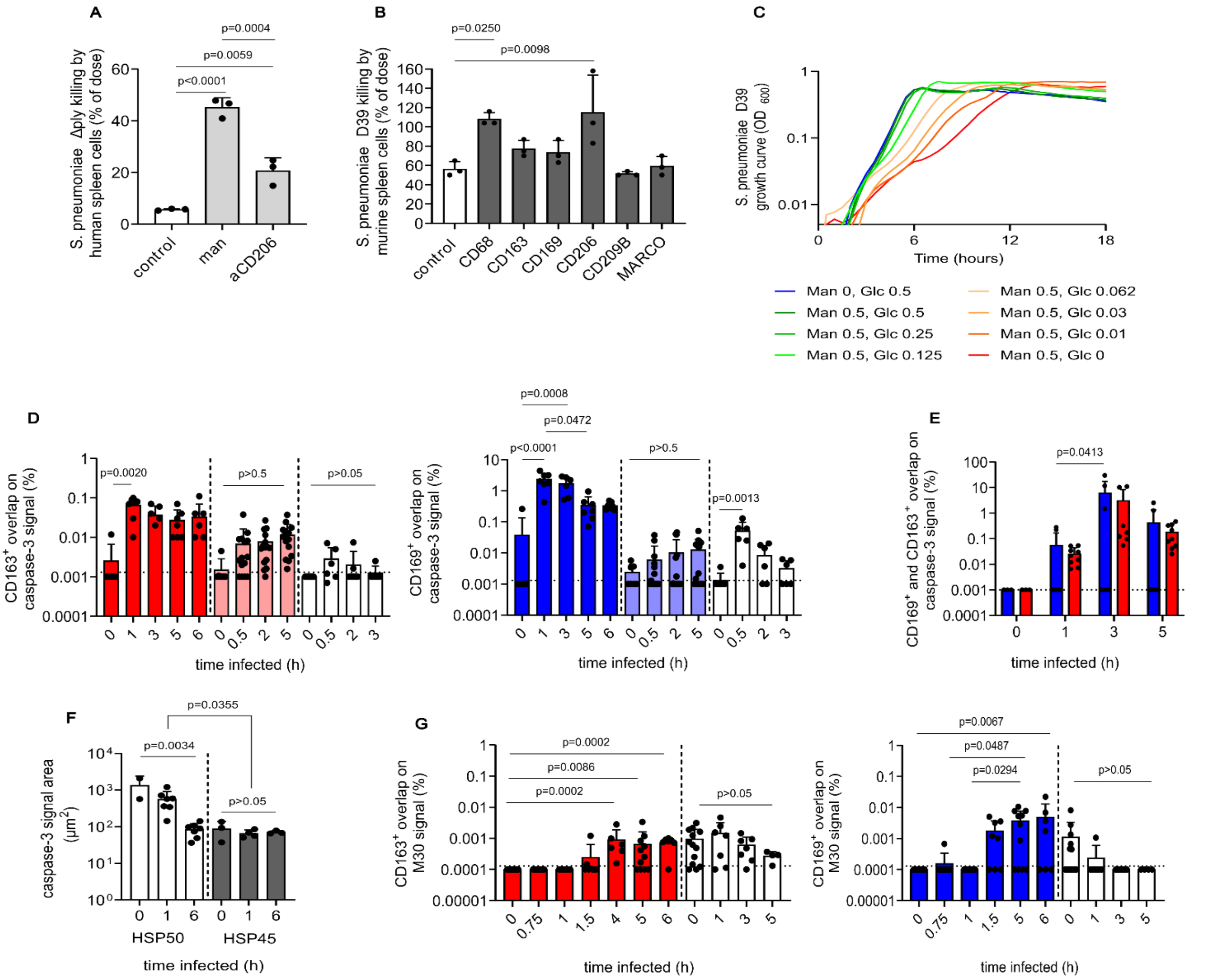

Supplementary Figure 3: **Bactericidal activity of human and murine splenic macrophages and macrophage-associated apoptotic markers.** Graphs show mean +/- SD. **A** Survival 30 min post-infection in primary human splenic cultures infected with an encapsulated pneumococcal pneumolysin (ply) deletion mutant under control conditions (white bars) or following addition of 5 mM mannose or anti-CD206 antibodies (grey bars). Data derive from n=3 independent wells (technical replicates). **B** Macrophage receptor blockade of bactericidal activity against the encapsulated pneumococcal serotype-2 strain D39 in murine primary splenic cultures. Dark-grey: macrophage inhibition; white bars: non-inhibited controls). Data derive from n=3 independent wells (technical replicates). **C** Growth curves (OD600) of pneumococcal serotype-2 strain D39 in the presence of a fixed mannose concentration and increasing glucose concentrations; each line represents a distinct condition. **D** Quantification of macrophage area positive for activated caspase-3 over time (CD163$^+$: red; CD169$^+$: blue; bead-perfused spleens: light-red/light-blue). Data derive from n=1 infected spleen, n=3 pooled bead-perfused spleens, and n=2 control spleens (biological groups). Technical replicates: infected 6–7 stamps, bead 7–15 stamps, control 6–8 stamps. **E** Quantification of activated caspase-3–positive macrophage area using QuPath cell-segmentation (CD163$^+$: red; CD169$^+$: blue). Data derive from n=1 infected spleen. Each dot represents a technical replicate: 3-9 stamps. **F** Quantification of tissue area positive for activated caspase-3 in a high-dose (10^8 CFU; white bars) versus a low-dose (10^7 CFU; grey bars) spleen. Each dot represents one stamp: 3-9 high-dose; 3-4 low-dose. **G** Quantification of M30-positive macrophage area in CD163$^+$ (red) and CD169$^+$ (blue) populations. Data derive from n=5 infected spleens and n=2 control spleens (biological groups). Technical replicates: infected 6–18 stamps, control 4–13 stamps. Each dot represents one stamp. Uninfected perfused spleens: white bars. Dotted lines indicate detection limits (0.0013% E; 0.00013% G). Statistical significance was determined by ordinary one-way ANOVA with Tukey's two-sided post-hoc test (A, B, F), non-parametric one-way ANOVA with Kruskal–Wallis post-hoc (Dunn's multiple comparisons two-sided) (D, E, G), and two-sided unpaired parametric t-test (B) (p values are shown in the figure). Source data are provided as a Source Data file. Spleens analysed are listed in Supplementary Table 5.

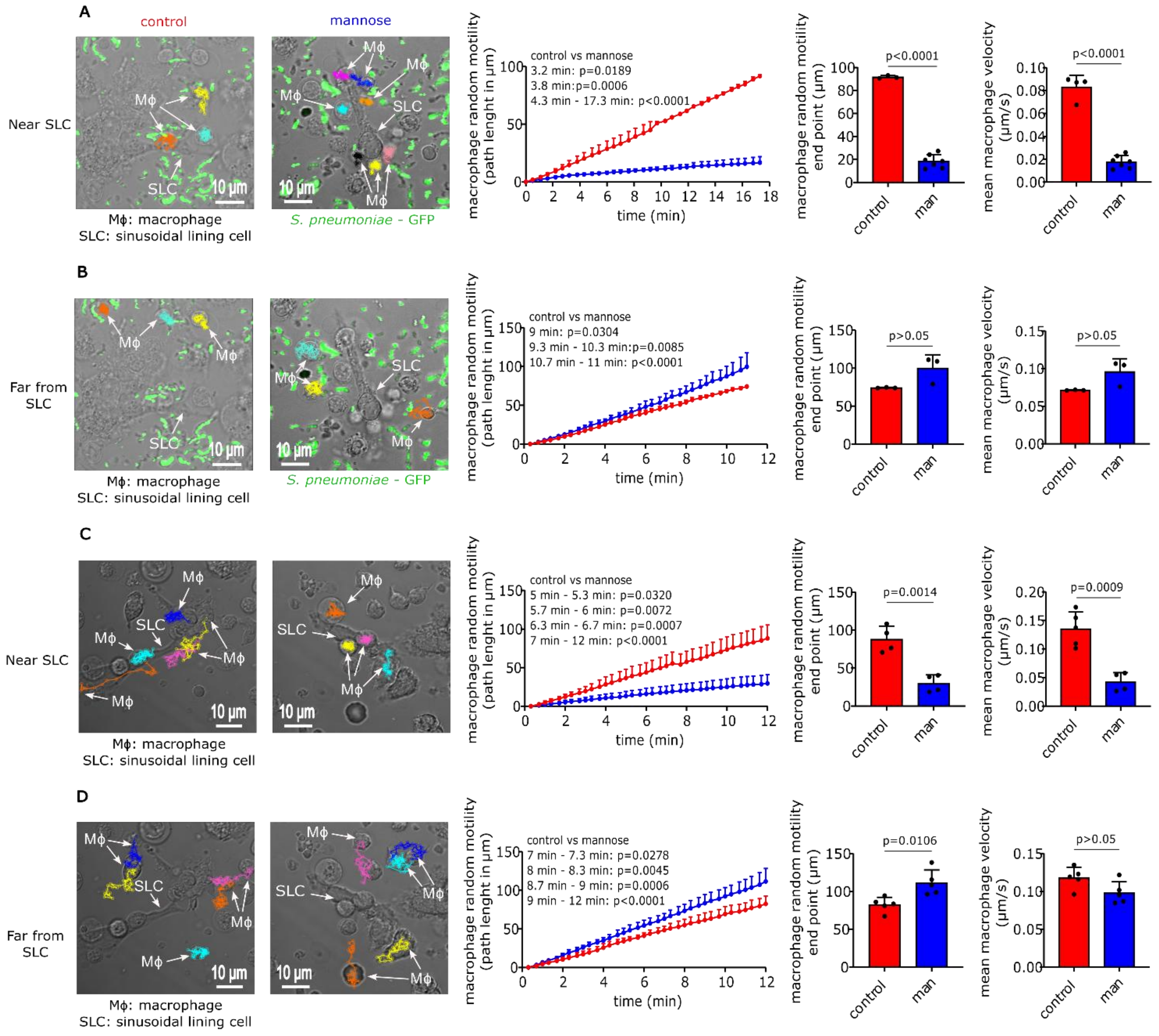

Supplementary Figure 4: **Tracking of macrophage dynamics in response to bacteria and mannose.** Graphs report mean +/- SD. **A** Time-lapse microscopy tracking of primary human macrophages located proximal to sinusoidal lining cells during infection with GFP-expressing pneumococci under control conditions (left) and 5 mM mannose supplementation (right). Analyses report macrophage random motility, endpoint random motility, and mean macrophage velocity in control (red) versus mannose-treated cultures (blue) (3 macrophages were tracked for the control conditions and 7 for the mannose supplementation). **B** Tracking of macrophages located distal from sinusoids during confocal time-lapse imaging of primary splenic cultures infected with GFP-expressing pneumococci under control conditions (left) and 5 mM mannose supplementation (right). Analyses report macrophage random motility, endpoint random motility, and mean macrophage velocity in control (red) versus mannose-treated cultures (blue) (3 macrophages were tracked for both the control conditions and mannose supplementation). **C** Quantitative analysis of time-lapse recordings showing macrophage random motility (control: red; mannose: blue) for cells proximal to sinusoidal lining cells in absence of bacteria (4 macrophages were tracked for both the control conditions and mannose supplementation). **D** Quantitative analysis of time-lapse recordings showing mean macrophage velocity (control: red; mannose: blue) for cells distal from sinusoids in absence of GFP-expressing pneumococci (5 macrophages were tracked for both the control conditions and mannose supplementation). In the microscopy panels, each line represents the trajectory of an individual macrophage during time-lapse tracking, illustrating cell movement over time. In the bar charts, each dot corresponds to the quantitative metrics derived from these tracked macrophage. Representative time-lapse videos are available at https://doi.org/10.6092/unibo/amsacta/8725 (Supplementary data 2). Abbreviations: SNL, sinusoidal lining cell; Mϕ, macrophage. Statistical significance was determined by two-way ANOVA with two-sided Šídák's

multiple comparisons test (xy graphs) and two-sided unpaired parametric t-test (bar-charts) (p values for all comparisons are shown in the figure). Source data are provided as a Source Data file.

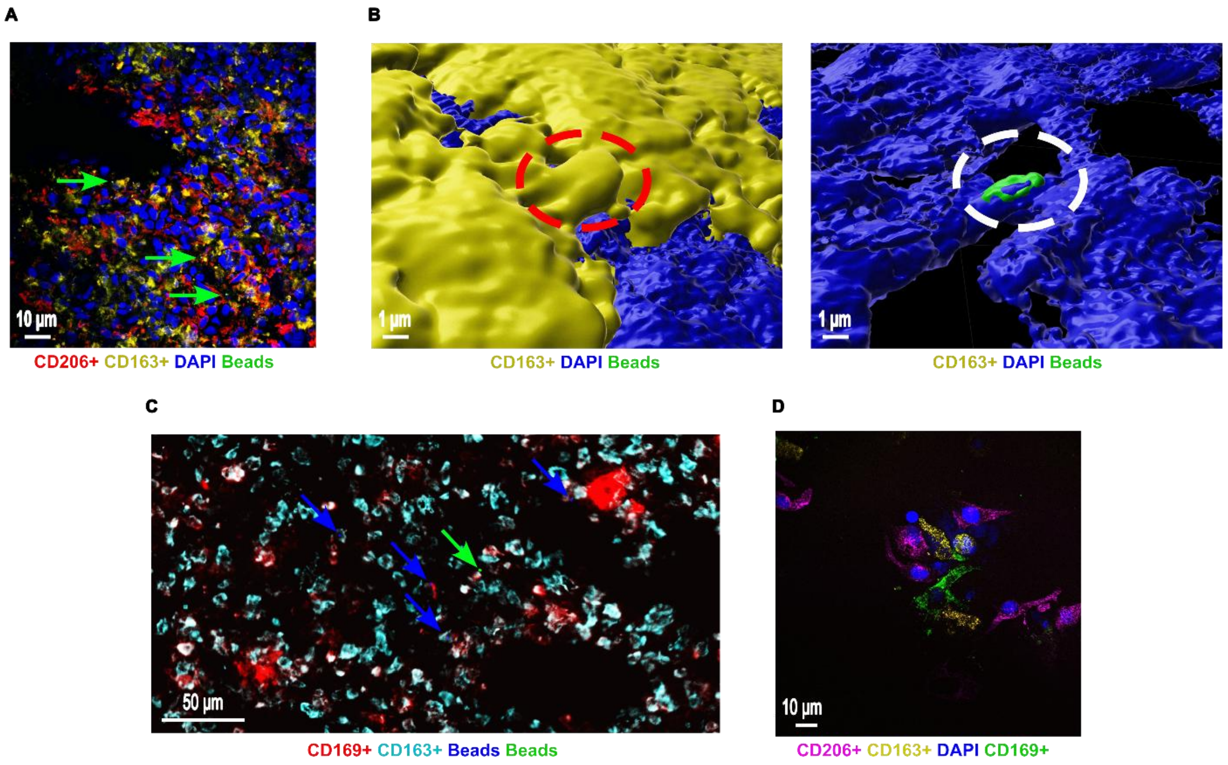

Supplementary Figure 5: **Micro-beads uptake by human spleen macrophages during *ex vivo* organ perfusion and primary cells macrophage composition**: **A** High-content confocal image of a spleen section showing fluorescent micro beads (green), CD163^{+} RPMs (yellow) and CD206^{+} sinusoidal lining cells (red). **B** 3D confocal reconstruction of bead (green) uptake by RPMs (yellow). The red and white dashed circles indicate the bead localization within the macrophage. **C** High-content scanning microscopy of a spleen section containing fluorescent micro-beads (blue), CD163^{+} RPMs (cyan) and CD169^{+} PCSAMs (red). **D** Confocal microscopy of the primary human splenic cell culture showing CD206^{+} sinusoidal lining cells (magenta), CD163^{+} RPMs (yellow) and CD169^{+} PCSAMs (green). Nuclei are shown in DAPI staining (blue). Source data are provided as a Source Data file. Spleens analysed in these panels are listed in Supplementary Table 5.

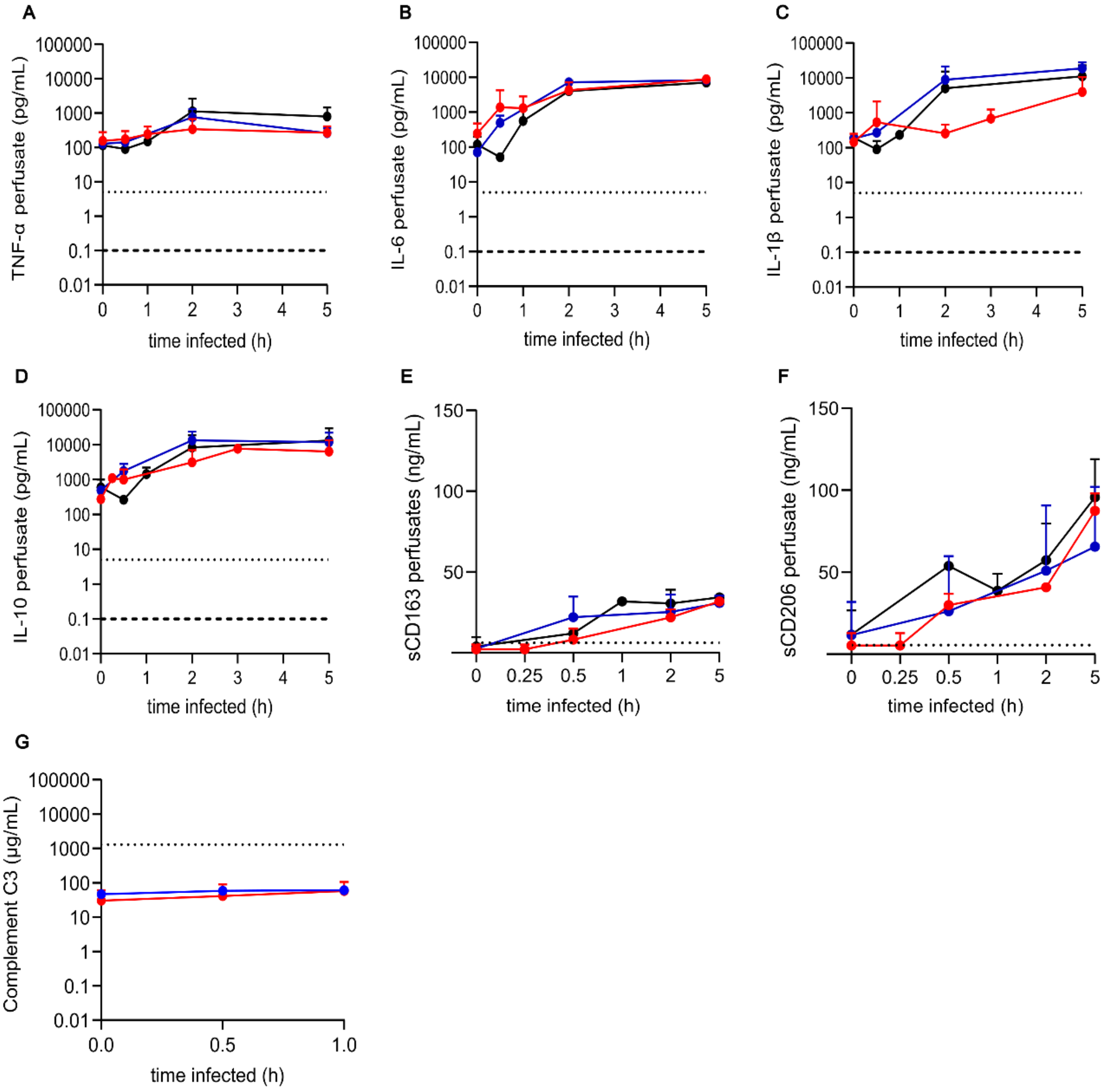

Supplementary Figure 6: **Cytokine, soluble CD163 and CD206 and complement C3 quantification in human spleen perfusate samples over time of perfusion.** Graphs report mean and standard deviation (SD). **A** Quantification of TNF-α over time (hours) of perfusion. **B** Quantification of IL-6 over time (hours) of perfusion. **C** Quantification of IL-1β over time (hours) of perfusion. **D** Quantification of IL-10 over time (hours) of perfusion. **E** ELISA quantification of soluble CD163. **F** ELISA quantification of soluble CD206. **G** ELISA quantification of complement C3. Dashed lines indicate detection limits (0.1 pg/mL for cytokines and soluble receptors; 5 ng/mL where applicable). Dotted lines show normal serum concentrations (5 pg/mL for cytokines and soluble markers; 1300 µg/mL for complement C3). Blue lines: pooled control spleens; red lines: pooled infected spleens; black lines: pooled mannose-treated spleens. A-D Data derive form n=18 infected human spleens, n=5 mannose treated spleens and n=6 control spleens. E-F Data derive form n=2 infected human spleens, n=3 mannose treated spleens and n=4 control spleens. G Data derive form n=3 infected human spleens and n=1 control spleen. Time is expressed in hours (h). Source data are provided as a Source Data file. Spleens analysed in these panels are listed in Supplementary Table 5.

References

1. Carreno, D., Wanford, J. J., Jasiunaite, Z., Hames, R. G., Chung, W. Y., Dennison, A. R. & Oggioni, M. R. Splenic macrophages as the source of bacteraemia during pneumococcal pneumonia. EBioMedicine 72 (2021).
2. Tettelin, H., Nelson, K. E., Paulsen, I. T., Eisen, J. A., Read, T. D., Peterson, S. & Fraser, C. M. Complete genome sequence of a virulent isolate of Streptococcus pneumoniae. Science 293, 498–506 (2001).
3. Lanie, J. A., Ng, W. L., Kazmierczak, K. M., Andrzejewski, T. M., Davidsen, T. M., Wayne, K. J. & Winkler, M. E. Genome sequence of Avery's virulent serotype 2 strain D39 of Streptococcus pneumoniae and comparison with that of unencapsulated laboratory strain R6. Journal of Bacteriology 189, 38–51 (2007).
4. McGee, L., McDougal, L., Zhou, J., Spratt, B. G., Tenover, F. C., George, R. & Klugman, K. P. Nomenclature of major antimicrobial-resistant clones of Streptococcus pneumoniae defined by the pneumococcal molecular epidemiology network. Journal of Clinical Microbiology 39, 2565–2571 (2001).
5. Pozzi, G., Masala, L., Iannelli, F., Manganelli, R., Havarstein, L. S., Piccoli, L. & Morrison, D. A. Competence for genetic transformation in encapsulated strains of Streptococcus pneumoniae: two allelic variants of the peptide pheromone. Journal of Bacteriology 178, 6087–6090 (1996).
6. Hiller, N. L., Janto, B., Hogg, J. S., Boissy, R., Yu, S., Powell, E. & Hu, F. Z. Comparative genomic analyses of seventeen Streptococcus pneumoniae strains: insights into the pneumococcal supragenome. Journal of Bacteriology 189, 8186–8195 (2007).
7. Wanford, J. J., Hames, R. G., Carreno, D., Jasiunaite, Z., Chung, W. Y., Arena, F. & Oggioni, M. R. Interaction of Klebsiella pneumoniae with tissue macrophages in a mouse infection model and ex-vivo pig organ perfusions: an exploratory investigation. The Lancet Microbe 2, e695–e703 (2021).
8. Hunstad, D. A., Justice, S. S., Hung, C. S., Lauer, S. R. & Hultgren, S. J. Suppression of bladder epithelial cytokine responses by uropathogenic Escherichia coli. Infection and Immunity 73, 3999–4006 (2005).
