# Supplementary material for: The mannose receptor on sinusoidal lining cells mediates two-step bacterial clearance in the human spleen
Source: Nat Commun. 2026 Apr 29;17:7595. doi: 10.1038/s41467-026-72430-8 (PMC13421466; doi:10.1038/s41467-026-72430-8)
Supplement: Supplementary file 2 — Description of Additional Supplementary Files [file 41467_2026_72430_MOESM2_ESM.pdf]

**Description of Additional Supplementary Files**

File Name: Supplementary Data 1
Description: README file for the full original dataset of the manuscript “The Mannose Receptor on Sinusoidal Lining Cells Mediates Two-Step Bacterial Clearance in the Human Spleen”. The data are available at the Repository AMS ACTA with the doi 10.6092/unibo/amsacta/8855 (https://amsacta.unibo.it/id/eprint/8855/).

File Name: Supplementary Data 2
Description: README file for the Timelapse microscopy dataset of the manuscript “The Mannose Receptor on Sinusoidal Lining Cells Mediates Two-Step Bacterial Clearance in the Human Spleen”. The data are available at the Repository AMS ACTA with the doi 10.6092/unibo/amsacta/8725 (https://amsacta.unibo.it/id/eprint/8725/).
