# Supplementary material for: The mannose receptor on sinusoidal lining cells mediates two-step bacterial clearance in the human spleen
Source: Nat Commun. 2026 Apr 29;17:7595. doi: 10.1038/s41467-026-72430-8 (PMC13421466; doi:10.1038/s41467-026-72430-8)
Supplement: Supplementary file 3 — Supplementary Data 1 [file 41467_2026_72430_MOESM3_ESM.docx]

# README file

Dataset Title: **Full original dataset - The Mannose Receptor on Sinusoidal Lining Cells Mediates Two-Step Bacterial Clearance in the Human Spleen**

Dataset Repository AMS ACTA DOI [10.6092/unibo/amsacta/8855](https://doi.org/10.6092/unibo%2Famsacta%2F8855) (<https://amsacta.unibo.it/id/eprint/8855/>)

Dataset Authors:

- **Francesco Flandi** (University of Bologna, Department of Pharmacy and Biotechnology)
- **Marco Rinaldo Oggioni** (University of Bologna, Department of Pharmacy and Biotechnology), ORCID 0000-0003-4117-793X

Data Set Contact Person

- **Marco Rinaldo Oggioni** (University of Bologna), email [marcorinaldo.oggioni@unibo.it](mailto:marcorinaldo.oggioni@unibo.it)

Data Set License:

This data set is distributed under a **Creative Commons Attribution 4.0 International (CC BY 4.0) license**, <https://creativecommons.org/licenses/by/4.0/>

Publication Year: 2026

Project Info: the research leading to these results received funding from

- INF-ACT (One Health Basic and Translational Research Actions addressing Unmet Needs on Emerging Infectious Diseases), funded by European Union, NextGenerationEU programme, PNRR Extended Partnership initiative on Emerging Infectious Diseases (PNRR, Missione 4 Componente 2 - Investimento 1.3), project number PE00000007, CUP ID [B13C22002440006](https://www.opencup.gov.it/portale/web/opencup/home/progetto/-/cup/B13C22002440006), <https://www.inf-act.it/>;
- CoDiCo (Transition from asymptomatic colonization to disease by human respiratory-tract bacteria as a target for vaccines and antimicrobial therapy: The CoDiCo (colonisation to disease concepts) project), funded by the Italian Ministry of University and Research under PRIN 2020 funding programme, project number 202089LLEH;
- PRIN 2022 PNRR project “The innate immune reaction to invasive infection in human organs”, funded through the PRIN 2022 PNRR programme (National Recovery and Resilience Plan, Missione 4 Componente 2 - Investimento 1.1), financed by the European Union within the NextGenerationEU programme. Grant number P2022M8KYE.

## Data set Contents

The dataset consists of:

- **8 compressed folders with 74 images in .tif, .oif.tif, .oif, .nd2 or .png formats, and 7 videos in .avi format:**
  - folder bacteria_red_pulp_localization_figureS2.zip
  - bacteria_sinusoids_3d_figure3.zip
  - fluorescent_beads_macrophages_uptake_figureS5.zip
  - human_spleen_marker_colocalization_figureS1.zip
  - human_spleen_scanning_microscopy_figure1.zip
  - macrophage_tracking_timelapse_figureS4.zip
  - primary_splenic_cell_culture_confocal_figure4.zip

splenic_cell_timelapse_microscopy_figure5.zip

- **1 source data file in .xlsx format containing the raw data used to generate the graphs**
  - alnabati_flandi_source_data.xlsx
- **1 README file in .docx**
  - Full original data and microscopy images Data-MicroscopyImages_HumanSpleenCells_BacterialClearance_README.docx

## Abstract

In the human spleen, encapsulated bacteria are first captured by CD206+ sinusoidal lining cells and subsequently transferred to CD163+ red pulp macrophages for killing, revealing a previously unrecognized two-step immune defence. We deposited the data supporting this study, including high-resolution imaging, 3D reconstructions, and time-lapse microscopy of human spleen sections and primary splenic cell cultures, showing the spatial organization and dynamic interactions of macrophages with sinusoidal lining cells for bacteria uptake and elimination. Specifically, the dataset includes high-content slide-scanning microscopy, confocal immunofluorescence, 3D reconstructions, and time-lapse macrophage tracking in the presence or absence of mannose during infection.

## Content of the files

- The folder bacteria_red_pulp_localization_figureS2.zip contains four sets of images, organized in subfolders, from a single human spleen (HSP67). These images show the open circulation of the human spleen red pulp (splenic_red_pulp_open_circulation folder) and samples collected at different time points after bacterial challenge: 30 min (bacteria_localization_30min folder), 2 h (bacteria_localization_2h folder), and 5 h (bacteria_localization_5h folder). Inside the .zip archive, each subfolder contains images corresponding to one of these conditions. The images show pneumococci (green) in the red pulp associated with CD206⁺ sinusoidal lining cells (cyan) and CD163⁺ red pulp macrophages (magenta), with nuclei stained with DAPI (blue/grey). Each set contains the images used in the paper in .png format (Supplementary Figure 2) and the corresponding original slide-scanning microscopy images in .tif format. Images are organized by experimental condition and time point for clarity.
- The folder bacteria_sinusoids_3d_figure3.zip contains three sets of confocal microscopy images and 3D reconstructions from human spleen sections (HSP51, HSP66) showing the interaction of pneumococci with splenic sinusoidal lining cells and red pulp macrophages. These datasets correspond to the panels shown in Figure 3 of the manuscript. Inside the .zip archive, the subfolders contain the following image sets:
  - 3d_bacteria_sinusoids_macrophages: 3D reconstruction showing the interaction between CD206⁺ sinusoidal lining cells (red), CD163⁺ red pulp macrophages (magenta), and bacteria (green). Nuclei are stained with DAPI (blue).
  - 3d_pneumococcus_binding_cd206: 3D confocal reconstruction showing the binding of pneumococci (green) to CD206⁺ sinusoidal lining cells (red). Nuclei are stained with DAPI (blue).
  - 3d_wga_cd206_bacteria: WGA staining (yellow) of spleen sections showing the localization of CD206 (red) and bacteria (green) on sinusoidal lining cells. Nuclei are labelled with DAPI (blue).

Each subfolder contains the processed images used in the paper in .png format (Figure 3 panels), the 3D reconstruction videos in .avi, and the original microscopy image files, including z-stacks in .tif or .nd2 format and Imaris reconstructions in .ims.

- The folder fluorescent_beads_macrophages_uptake_figureS5.zip contains four sets of microscopy images from a human spleen (HSP54) section and primary human splenic cell cultures showing the uptake of fluorescent microbeads by splenic macrophages and the composition of the primary cell cultures. These datasets correspond to the panels shown in Supplementary Figure 5 of the manuscript. Inside the .zip archive, the following subfolders contain the different image sets:
  - bead_uptake_confocal_spleen: high-content confocal image of a spleen section showing fluorescent microbeads (green), CD163⁺ red pulp macrophages (yellow), CD206⁺ sinusoidal lining cells (red), and nuclei in DAPI (blue).
  - bead_uptake_macropage_3d: 3D confocal reconstruction showing the uptake of fluorescent beads (green) by CD163⁺ red pulp macrophages (yellow). Cells nuclei are shoen in DAPI staining (blue).
  - beads_localization_scanning: high-content slide-scanning microscopy image of a spleen section containing fluorescent microbeads (blue), CD163⁺ red pulp macrophages (cyan), and CD169⁺ PCSAMs (red).
  - primary_cells_cd163_cd169_cd206: confocal microscopy of primary human splenic cell cultures showing CD206⁺ sinusoidal lining cells (magenta), CD163⁺ red pulp macrophages (yellow), and CD169⁺ PCSAMs (green). Nuclei are stained with DAPI (blue).

The folder contains the processed image used in the paper in .png format, the 3D video in .avi and the corresponding original confocal and slide scanning microscopy image files in .tif.

- The folder human_spleen_marker_colocalization_figureS1.zip contains seven sets of slide-scanning microscopy images from human spleen sections showing the colocalization and spatial distribution of macrophage and endothelial markers, as well as the localization of bacteria within splenic tissue. These datasets correspond to the panels shown in Supplementary Figure 1 of the manuscript. Inside the .zip archive, the following subfolders contain the different image sets:
  - cd31_cd206_bacteria_localization: whole-section scanning microscopy images showing CD31⁺ endothelial cells (red), CD206⁺ sinusoidal lining cells (cyan), and bacteria (green). Nuclei are stained with DAPI (blue).
  - cd68_cd163_colocalization: high-content scanning microscopy images showing the colocalization between the macrophage markers CD68 (magenta) and CD163 (cyan).
  - cd68_cd169_colocalization: scanning microscopy images showing the overlap between the CD68 (magenta) and CD169 (cyan) signals.
  - cd169_high_density_spleen: image showing inter-individual variability in the distribution of CD169⁺ PCSAMs in the human spleen sample HSP51, characterized by a high density of CD169⁺ cells. Cyan: CD206⁺ sinusoidal lining cells; Red: CD169⁺ PCSAMs; Nuclei stained with DAPI (blue).
  - cd169_low_density_spleen: images showing inter-individual variability in the distribution of CD169⁺ PCSAMs in the human spleen sample HSP66, characterized by a low density of CD169⁺ cells. Cyan: CD163⁺ RPMs; Red: CD169⁺ PCSAMs; Nuclei stained with DAPI (blue).
  - cd169_medium_density_spleen: images showing inter-individual variability in the distribution of CD169⁺ PCSAMs in the human spleen sample HSP50, characterized by a medium density of CD169⁺ cells. Cyan: CD206⁺ sinusoidal lining cells; Red: CD169⁺ PCSAMs; Nuclei stained with DAPI (blue).
  - cd206_cd31_distribution: low magnification images showing the distinct distribution of CD206 (cyan) and CD31 (red) markers in human spleen tissue. Nuclei are stained with DAPI (blue).

The folder contains the processed image used in the paper in .png format and the corresponding original slide-scanning microscopy image in .tif.

- The folder human_spleen_scanning_microscopy_figure1.zip contains representative high-content fluorescent slide-scanning microscopy images of a human spleen section (HSP63) showing the spatial distribution of splenic macrophage populations. These datasets correspond to the panels shown in Figure 1 of the manuscript. Inside the .zip archive, the images show CD206⁺ sinusoidal lining cells (cyan), CD163⁺ red pulp macrophages (RPMs, magenta), and CD169⁺ PCSAMs (red), with nuclei counterstained using DAPI (grey). The folder contains the two processed images used in the paper in .png format (panels A and B of Figure 1) and the corresponding original slide-scanning microscopy image in .tif format.
- The folder macrophage_tracking_timelapse_figureS4.zip contains datasets used to analyse macrophage tracking dynamics in primary human splenic cell cultures under different experimental conditions. These datasets correspond to the panels shown in Supplementary Figure 4 of the manuscript. Inside the .zip archive, four subfolders organize the datasets according to infection status and treatment condition:
  - infected_macrophage_tracking_control: macrophage tracking in infected cultures under control conditions.
  - infected_macrophage_tracking_mannose: macrophage tracking in infected cultures in the presence of 5 mM mannose
  - uninfected_macrophage_tracking_control: macrophage tracking in non-infected cultures under control conditions
  - uninfected_macrophage_tracking_mannose: macrophage tracking in non-infected cultures in the presence of mannose

Each subfolder contains the processed images used in the paper in .png format showing proximal and distal macrophage tracking panels. The corresponding time-lapse microscopy videos used for the analysis have been deposited separately in the repository and are accessible through the dataset <https://doi.org/10.6092/unibo/amsacta/8725>. The same videos are shared between paired panels (proximal/distal macrophages) within the same experimental condition.

- The folder primary_splenic_cell_culture_confocal_figure4.zip contains confocal microscopy images and 3D reconstructions from primary human splenic cell cultures used to characterize splenic cell populations and their interaction with pneumococci. These datasets correspond to the panels shown in Figure 4 of the manuscript. Inside the .zip archive, the following subfolders contain the different image sets:
  - bacteria_surface_biding_sinusoids: 3D confocal reconstruction showing the interaction between bacteria (green) and splenic sinusoids (red) in primary splenic cell cultures, highlighting the localization of bacteria on the surface of splenic sinusoids. Cell nuclei are shown in DAPI (blue).
  - culture_characterization_cd163_cd206: immunofluorescence confocal microscopy characterization of primary splenic cell cultures. CD163⁺ macrophages are shown in yellow, CD206⁺ sinusoidal lining cells in red, CD163⁻CD206⁻CD14⁺ cells in green, and cell nuclei in DAPI (blue).
  - intracellular_bacteria_cd163: 3D confocal reconstruction of the interaction between bacteria (green) and CD163⁺ red pulp macrophages (yellow) in primary splenic cell cultures. Cell nuclei are shown in DAPI staining (blue).
  - lyve1_expression_cd206_cells: confocal microscopy showing LYVE-1 (yellow) expression in CD206⁺ cells (red) in primary human splenic cell cultures. Cell nuclei are stained with DAPI (blue).
  - pneumococcus_localization_sinusoidal_cells: confocal microscopy showing the localization of pneumococci (green) in relation to CD206⁺ sinusoidal lining cells (red). Cell nuclei are shown in DAPI staining (blue).

Each subfolder contains the processed image used in the paper in .png format, the corresponding original slide-scanning microscopy image in .tif, 3d videos in .avi and Imaris 3D reconstructions in .ims format.

- The folder splenic_cell_timelapse_microscopy_figure5.zip contains confocal time-lapse microscopy datasets from primary human splenic cell cultures, corresponding to panels of Figure 5 in the manuscript. These datasets capture dynamic behavior of splenic cells in the absence of GFP-expressing pneumococci. Inside the .zip archive, the subfolders contain the following image sets:
  - control_cell_culture_timelapse: confocal time-lapse microscopy of a primary splenic cell culture without GFP-expressing pneumococci.
  - infection_cell_culture_timelapse: confocal time-lapse microscopy of a primary splenic cell culture infected with GFP-expressing pneumococci.

Each subfolder contains the processed images used in the paper in .png format and the original miscroscope file in .nd2 format. Data access: have already been deposited in the repository. Time-lapse videos can be accessed via the DOI: <https://doi.org/10.6092/unibo/amsacta/8725>.

- The file alnabati_flandi_source_data.xlsx contains the raw data used in the manuscript for the generation of the graphs:
  - Tab 1C: raw data used to generate the panel C of figure 1. It contains the percent of the spleen area occupied by CD169^+^ macrophages, CD163^+^ macrophages and CD206^+^ sinusoidal lining cells pooled from three independent human spleens.
  - Tab 1D: : raw data used to generate the panel D of figure 1. The numbers refer to the area in μm^2^ occupied by the CD206 marker per mm^2^ of tissue across 11 human spleens.
  - Tab 1E: : raw data used to generate the panel E of figure 1. The numbers refer to the area in μm^2^ occupied by the CD163 marker per mm^2^ of tissue across 19 human spleens.
  - Tab 1F: : raw data used to generate the panel F of figure 1. The numbers refer to the area in μm^2^ occupied by the CD169 marker per mm^2^ of tissue across 18 human spleens.
  - Tab 2A (left): raw data used to generate the left graph of panel A of figure 2. The numbers refer to the colony forming unit (CFU) per milliliter (mL) of perfusion liquid over time (hour).
  - Tab 2A (right): raw data used to generate the right graph of panel A of figure 2. The numbers refer to the colony forming unit (CFU) per gram (g) of perfusion liquid over time (hour).
  - Tab 2B (left): raw data used to generate the left graph of panel B of figure 2. The numbers refer to the colony forming unit (CFU) per milliliter (mL) of perfusion liquid over time (hour).
  - Tab 2B (right): raw data used to generate the right graph of panel B of figure 2. The numbers refer to the colony forming unit (CFU) per gram (g) of perfusion liquid over time (hour).
  - Tab 2C (left): raw data used to generate the left graph of panel C of figure 2. The numbers refer to the colony forming unit (CFU) per milliliter (mL) of perfusion liquid over time (hour).
  - Tab 2C (right): raw data used to generate the right graph of panel C of figure 2. The numbers refer to the colony forming unit (CFU) per gram (g) of perfusion liquid over time (hour).
  - Tab 2D (left): raw data used to generate the left graph of panel D of figure 2. The numbers refer to the colony forming unit (CFU) per milliliter (mL) of perfusion liquid over time (hour).
  - Tab 2D (right): raw data used to generate the right graph of panel D of figure 2. The numbers refer to the colony forming unit (CFU) per gram (g) of perfusion liquid over time (hour).
  - Tab 2E (left): raw data used to generate the left graph of panel E of figure 2. The numbers refer to the colony forming unit (CFU) per milliliter (mL) of perfusion liquid over time (hour).
  - Tab 2E (right): raw data used to generate the right graph of panel E of figure 2. The numbers refer to the colony forming unit (CFU) per gram (g) of perfusion liquid over time (hour).
  - Tab 2F (left): raw data used to generate the left graph of panel F of figure 2. The numbers refer to the colony forming unit (CFU) per milliliter (mL) of perfusion liquid over time (hour).
  - Tab 2F (right): raw data used to generate the right graph of panel F of figure 2. The numbers refer to the colony forming unit (CFU) per gram (g) of perfusion liquid over time (hour).
  - Tab 3A (left): raw data used to generate the left graph of panel A of figure 3. The numbers refer to the percent of overlap between the bacteria and the CD206 channels in the spleen tissue.
  - Tab 3A (right): raw data used to generate the right graph of panel A of figure 3. The numbers refer to the percent of overlap between the CD206 and the bacteria channels in the spleen tissue.
  - Tab 3D (left): raw data used to generate the left graph of panel D of figure 3. The numbers refer to the percent of overlap between the bacteria and the CD163 channels in the spleen tissue.
  - Tab 3D (right): raw data used to generate the right graph of panel D of figure 3. The numbers refer to the percent of overlap between the CD163 and the bacteria channels in the spleen tissue.
  - Tab 3E (left): raw data used to generate the left graph of panel E of figure 3. The numbers refer to the percent of overlap between the bacteria and the CD169 channels in the spleen tissue.
  - Tab 3E (right): raw data used to generate the right graph of panel E of figure 3. The numbers refer to the percent of overlap between the CD163 and the bacteria channels in the spleen tissue.
  - Tab 4A: raw data used to generate the graph of panel A of figure 4. The numbers refer to the composition in percent of CD163^+^, CD206^+^ and CD14^+^ cells in the primary splenic cell culture.
  - Tab 4B (left): raw data used to generate the left graph of panel B of figure 4. The numbers refer to the percent of overlap between the bacteria and the CD206 channels in the primary cell culture, under control conditions or mannose (man) and anti-CD206 antibody (αCD206) supplementation.
  - Tab 4B (right): raw data used to generate the right graph of panel B of figure 4. The numbers refer to the percent of overlap between the bacteria and the CD163 channels in the primary cell culture, under control conditions, mannose (man) and anti-CD206 antibody (αCD206) supplementation or a combination of both.
  - Tab 5A (left): raw data used to generate the left graph of panel A of figure 5. The numbers refer to the bacterial survival in percent of challenge dose under control conditions, mannose (man) and anti-CD206 antibody (αCD206) supplementation, or a combination of both. This graph refers to the TIGR4 pneumococcal strain after the infection of the primary cell culture
  - Tab 5A (right): raw data used to generate the right graph of panel A of figure 5. The numbers refer to the bacterial survival in percent of challenge dose under control conditions or mannose (man) supplementation. This graph refers to the Δcps derivative of the TIGR4 pneumococcal strain after the infection of the primary cell culture
  - Tab 5B: raw data used to generate the panel B of figure 5. The numbers refer to the bacterial survival in percent of challenge dose for different pneumococcal serotypes under control conditions or mannose (man) supplementation, after the infection of the primary cell culture.
  - Tab 5C (left): raw data used to generate the left graph of panel C of figure 5. The numbers refer to the bacterial survival in percent of challenge dose under control conditions, mannose (man) and anti-CD206 antibody (αCD206) supplementation, or a combination of both. This graph refers to the *Klebsiella pneumoniae* strain GMR151 after the infection of the primary cell culture.
  - Tab 5C (right): raw data used to generate the right graph of panel C of figure 5. The numbers refer to the bacterial survival in percent of challenge dose under control conditions or mannose (man) supplementation. This graph refers to the *Escherichia coli* strain UTI89 after the infection of the primary cell culture
  - Tab 5D (left): raw data used to generate the left graph of panel D of figure 5. The numbers refer to the bacterial survival in percent of challenge dose after the inhibition of macrophage receptors (dark-grey bars) or sinusoidal lining cells markers (light-grey bars) or under non-inhibited control condition (white bar). This graph refers to the D39 pneumococcal strain after the infection of the primary cell culture.
  - Tab 5D (right): raw data used to generate the right graph of panel D of figure 5. The numbers refer to the bacterial survival in percent of challenge dose after the inhibition of macrophage receptors (dark-grey bars) or sinusoidal lining cells markers (light-grey bars) or under non-inhibited control condition (white bar). This graph refers to a Δcps derivative of the D39 pneumococcal strain after the infection of the primary cell culture
  - Tab 5F: raw data used to generate the panel F of figure 5. The numbers refer to the macrophage random motility in μm under control conditions (white symbols) or mannose supplementation (grey symbols).
  - Tab 5G: raw data used to generate the panel G of figure 5. Values represent the percentage of cell-associated bacteria relative to the total number of bacteria over the course of the time-lapse imaging (minutes), under control conditions (white symbols) or in the presence of mannose supplementation (grey symbols).
  - Tab 5H: raw data used to generate the panel H of figure 5. The values represent the number of bacteria per macrophage located near to sinusoidal lining cells (white) or distal form them (grey).
  - Tab 6A (left): raw data used to generate the left graph of panel A of figure 6. The numbers refer to the percent of overlap between the bacteria (red) or fluorescence micro-beads (light-red) channels and the CD163 signal in the spleen tissue.
  - Tab 6A (right): raw data used to generate the right graph of panel A of figure 6. The numbers refer to the percent of overlap between the bacteria (blue) or fluorescence micro-beads (light-blue) channels and the CD169 signal in the spleen tissue.
  - Tab 6B (left): raw data used to generate the left graph of panel B of figure 6. The numbers refer to the percent of overlap between the bacteria and the CD163 signal in the spleen tissue across different human spleen.
  - Tab 6B (right): raw data used to generate the left graph of panel B of figure 6. The numbers refer to the percent of overlap between the bacteria and the CD169 signal in the spleen tissue across different human spleen.
  - Tab 6C: raw data used to generate the left graph of panel C of figure 6. The numbers refer to the ration between the LAMP1 area in um2 and the DAPI area of the analyzed tissue regions across different human spleen
  - Tab 6D (left): raw data used to generate the left graph of panel D of figure 6. The numbers refer to the percent of overlap between the LAMP1 and the CD163 channels in the spleen tissue.
  - Tab 6D (right): raw data used to generate the right graph of panel D of figure 6. The numbers refer to the percent of overlap between the LAMP1 and the CD169 channels in the spleen tissue.
  - Tab 6E (left): raw data used to generate the left graph of panel E of figure 6. The numbers refer to the percent of overlap between the cleaved caspase-3 and the CD163 channels in the spleen tissue.
  - Tab 6E (right): raw data used to generate the right graph of panel E of figure 6. The numbers refer to the percent of overlap between the cleaved caspase-3 and the CD169 channels in the spleen tissue.
  - Tab 6F (left): raw data used to generate the left graph of panel F of figure 6. The numbers refer to the percent of overlap between the M30 and the CD163 channels in the spleen tissue.
  - Tab 6F (right): raw data used to generate the right graph of panel F of figure 6. The numbers refer to the percent of overlap between the M30 and the CD169 channels in the spleen tissue.
  - Tab Suppl. 1F (left): raw data used to generate the left graph of panel F of supplementary figure 1. The numbers refer to the tissue area in μm^2^ occupied by the CD163 marker.
  - Tab Suppl. 1F (right): raw data used to generate the right graph of panel F of supplementary figure 1. The numbers refer to the tissue area in μm^2^ occupied by the CD206 marker.
  - Tab Suppl. 1G: raw data used to generate the right graph of panel G of supplementary figure 1. The numbers refer to the ratio between tissue area in μm^2^ occupied by the CD206 marker and the tissue area in μm^2^ occupied by the CD163 marker.
  - Tab Suppl. 2A (left): raw data used to generate the left graph of panel A of supplementary figure 2. The numbers refer to the percent of overlap between the type-4 and the type-19F serotypes fluorescence signals and the CD163 and CD169 channels in the spleen tissue across different human spleens.
  - Tab Suppl. 2A (right): raw data used to generate the right graph of panel A of supplementary figure 2. The numbers refer to the percent of overlap between the CD163 and CD169 channels and the type-4 and the type-19F serotypes fluorescence signals in the spleen tissue across different human spleens.
  - Tab Suppl. 3A: raw data used to generate the graph of panel A of supplementary figure 3. The numbers refer to the bacterial survival in percent of challenge dose under control conditions and mannose (man) or anti-CD206 antibody (αCD206) supplementation. This graph refers to the survival to the primary cell culture for a pneumococcal pneumolysin (ply) mutant.
  - Tab Suppl. 3B: raw data used to generate the graph of panel B of supplementary figure 3. The numbers refer to the bacterial survival in percent of challenge dose after the inhibition of macrophage receptors (dark-grey bars) or under non-inhibited control condition (white bar). This graph refers to the D39 pneumococcal strain after the infection of murine the primary cell culture.
  - Tab Suppl. 3C: raw data used to generate the graph of panel C of supplementary figure 3. The numbers refer to the to the OD_600nm_ over time (hours) for the pneumococcal strain D39.
  - Tab Suppl. 3D (left): raw data used to generate the left graph of panel D of supplementary figure 3. The numbers refer to the percent of overlap between the CD163 and the cleaved caspase-3 channels in the spleen tissue.
  - Tab Suppl. 3D (right): raw data used to generate the right graph of panel D of supplementary figure 3. The numbers refer to the percent of overlap between the CD169 and the cleaved caspase-3 channels in the spleen tissue.
  - Tab Suppl. 3E: raw data used to generate the left graph of panel E of supplementary figure 3. The numbers refer to the percent of overlap between the CD163 (red) and CD169 (blue) signals and the cleaved caspase-3 channels in the spleen tissue calculated in QuPath.
  - Tab Suppl. 3F: raw data used to generate the right graph of panel F of supplementary figure 3. The numbers refers to the spleen tissue area in μm^2^ occupied by the cleaved caspase-3 signals.
  - Tab Suppl. 3G (left): raw data used to generate the left graph of panel G of supplementary figure 3. The numbers refer to the percent of overlap between the CD163 and the M30 channels in the spleen tissue.
  - Tab Suppl. 3G (right): raw data used to generate the right graph of panel G of supplementary figure 3. The numbers refer to the percent of overlap between the CD169 and the M30 channels in the spleen tissue.
  - Tab Suppl. 4A (left): raw data used to generate the left graph of panel A of supplementary figure 4. The numbers refer to the macrophage random motility in μm under control conditions (red symbols) or mannose supplementation (blue symbols) for infected macrophages located near to sinusoidal lining cells.
  - Tab Suppl. 4A (central): raw data used to generate the central graph of panel A of supplementary figure 4. The numbers refer to the macrophage distance in μm covered under control conditions (red symbols) or mannose supplementation (blue symbols) for infected macrophages located near to sinusoidal lining cells.
  - Tab Suppl. 4A (right): raw data used to generate the right graph of panel A of supplementary figure 4. The numbers refer to the mean macrophage velocity (μm/s) under control conditions (red symbols) or mannose supplementation (blue symbols) for infected macrophages located near to sinusoidal lining cells.
  - Tab Suppl. 4B (left): raw data used to generate the left graph of panel B of supplementary figure 4. The numbers refer to the macrophage random motility in μm under control conditions (red symbols) or mannose supplementation (blue symbols) for infected macrophages located distal from sinusoidal lining cells.
  - Tab Suppl. 4B (central): raw data used to generate the central graph of panel B of supplementary figure 4. The numbers refer to the macrophage distance in μm covered under control conditions (red symbols) or mannose supplementation (blue symbols) for infected macrophages located distal from sinusoidal lining cells.
  - Tab Suppl. 4B (right): raw data used to generate the right graph of panel B of supplementary figure 4. The numbers refer to the mean macrophage velocity (μm/s) under control conditions (red symbols) or mannose supplementation (blue symbols) for infected macrophages located distal from sinusoidal lining cells.
  - Tab Suppl. 4C (left): raw data used to generate the left graph of panel C of supplementary figure 4. The numbers refer to the macrophage random motility in μm under control conditions (red symbols) or mannose supplementation (blue symbols) for non-infected macrophages located near to sinusoidal lining cells.
  - Tab Suppl. 4C (central): raw data used to generate the central graph of panel C of supplementary figure 4. The numbers refer to the macrophage distance in μm covered under control conditions (red symbols) or mannose supplementation (blue symbols) for non-infected macrophages located near to sinusoidal lining cells.
  - Tab Suppl. 4C (right): raw data used to generate the right graph of panel C of supplementary figure 4. The numbers refer to the mean macrophage velocity (μm/s) under control conditions (red symbols) or mannose supplementation (blue symbols) for non-infected macrophages located near to sinusoidal lining cells.
  - Tab Suppl. 4D (left): raw data used to generate the left graph of panel D of supplementary figure 4. The numbers refer to the macrophage random motility in μm under control conditions (red symbols) or mannose supplementation (blue symbols) for non-infected macrophages located distal from sinusoidal lining cells.
  - Tab Suppl. 4D (central): raw data used to generate the central graph of panel D of supplementary figure 4. The numbers refer to the macrophage distance in μm covered under control conditions (red symbols) or mannose supplementation (blue symbols) for non-infected macrophages located distal from sinusoidal lining cells.
  - Tab Suppl. 4D (right): raw data used to generate the right graph of panel D of supplementary figure 4. The numbers refer to the mean macrophage velocity (μm/s) under control conditions (red symbols) or mannose supplementation (blue symbols) for non-infected macrophages located distal from sinusoidal lining cells.
  - Tab Suppl. 6A: raw data used to generate the right graph of panel A of supplementary figure 6. Numbers reports the ELISA values in pg/mL of TNF-α in the perfusion liquid across spleens.
  - Tab Suppl. 6B: raw data used to generate the right graph of panel B of supplementary figure 6. Numbers reports the ELISA values in pg/mL of IL-6 in the perfusion liquid across spleens.
  - Tab Suppl. 6C: raw data used to generate the right graph of panel C of supplementary figure 6. Numbers reports the ELISA values in pg/mL of IL-1β in the perfusion liquid across spleens.
  - Tab Suppl. 6D: raw data used to generate the right graph of panel D of supplementary figure 6. Numbers reports the ELISA values in pg/mL of IL-10 in the perfusion liquid across spleens.
  - Tab Suppl. 6E: raw data used to generate the right graph of panel E of supplementary figure 6. Numbers reports the ELISA values in ng/mL of sCD163 in the perfusion liquid across spleens.
  - Tab Suppl. 6F: raw data used to generate the right graph of panel F of supplementary figure 6. Numbers reports the ELISA values in ng/mL of sCD206 in the perfusion liquid across spleens.
  - Tab Suppl. 6G: raw data used to generate the right graph of panel G of supplementary figure 6. Numbers reports the ELISA values in μg/mL of complement-C3 in the perfusion liquid across spleens.

## Data sources

Human spleens were obtained through Clinical Trial ”Tissue Models for Invasive Disease” (TIMID), IRAS 219992, REC 18/EM/0057, ClinicalTrials gov NCT04620824 of the University of Leicester while human primary cells were obtained through clinical trial 668_2023_Sper_AOUBo 19.10.2023 of the University of Bologna.

Methodology

**Histology and Immunostaining**

Fresh OCT-frozen human spleen tissue biopsies, obtained during *ex vivo* perfusions, were sectioned and processed for high-content imaging and immunofluorescence to visualize specialized macrophage subsets, sinusoidal lining cells, and bacterial localization.

- **Markers:** CD206 (sinusoidal lining cells), CD163 (red pulp macrophages, RPMs), CD169 (perifollicular capillary sheath macrophages, PCSAMs), CD31 (endothelial cells), CD68 (macrophages), Lyve-1 (sinusoids and endothelial cells), CD14 (monocytes/other myeloid cells), DAPI (nuclei), WGA (glycoproteins), bacterial antisera, and fluorescent microbeads.
- **Techniques:** Immunohistochemistry, high-content slide scanning, and confocal immunofluorescence were performed to analyze cell types, bacteria, and bead uptake.

Fluorescently labelled tissue sections were imaged using the Vectra Polaris Automated Quantitative Imaging System (Akoya Biosciences, MA, USA) at 40x magnification (NA = 0.75) in fluorescence mode, accessed via the Core Biotechnology Services Advanced Imaging Facility at the University of Leicester. Exposure times were optimized for each antibody channel. Image processing began with spectral unmixing in Phenochart (v1.1), followed by export of 6–8 representative image regions (“stamps”) using InForm software (v2.5.1, Akoya Biosciences). All images were saved in .tiff format and analyzed quantitatively in Fiji (v1.53).

Confocal imaging of spleen tissue sections and primary splenic cell cultures was performed using an Olympus FV1000 confocal laser scanning microscope with 40x (UPlanFLN 40x/NA = 1.3) and 60x (UPlan-SAPO 60x/NA = 1.35) objectives. Image processing and analysis were conducted using Fiji (v1.53).

**Confocal Microscopy and 3D Reconstruction**

Confocal images of spleen tissue and primary cells were acquired using an Olympus FV1000 confocal laser scanning microscope with 40x (UPlanFLN 40x/NA = 1.3) and 60x (UPlan-SAPO 60x/NA = 1.35) objectives. Image processing was performed using Fiji (v1.53). For 3D visualization, multi-plane Z-stack images were deconvolved using Huygens Essential deconvolution software (v18.04.1p0 64-bit; SVI, Hilversum, Netherlands) and reconstructed using Imaris 3D rendering software.

**Time-Lapse Microscopy**

Time-lapse confocal imaging of primary splenic cells was performed using a Nikon A1R+ confocal laser scanning microscope with resonant scanning and a 60× objective at the University of Bologna microscopy facility. Cells were seeded at 1x10^5^ per well on black, glass-bottom 96-well plates (Corning) and infected with a GFP-expressing S. pneumoniae strain at an MOI of 10. Imaging was conducted at 37 °C for approximately 20 minutes, acquiring frames every 20 seconds. Image processing and cell tracking were performed in Fiji3 using the MTrackJ plugin (<https://doi.org/10.6092/unibo/amsacta/8725>).

**Data Organization**

- Images are grouped by experiment, tissue type, or time point in the .zip archives.
- Each folder contains processed images for publication (.png) and original microscopy files for reproducibility (.tif, .nd2, .oif, .ims).
- Shared time-lapse videos (e.g., macrophage tracking) are referenced without duplication (<https://doi.org/10.6092/unibo/amsacta/8725>).
