# Supplementary material for: The mannose receptor on sinusoidal lining cells mediates two-step bacterial clearance in the human spleen
Source: Nat Commun. 2026 Apr 29;17:7595. doi: 10.1038/s41467-026-72430-8 (PMC13421466; doi:10.1038/s41467-026-72430-8)
Supplement: Supplementary file 4 — Supplementary Data 2 [file 41467_2026_72430_MOESM4_ESM.docx]

# README file

Dataset Title: [Timelapse microscopy dataset - The Mannose Receptor on Sinusoidal Lining Cells Mediates Two-Step Bacterial Clearance in the Human Spleen]

Dataset Repository AMS ACTA DOI <10.6092/unibo/amsacta/8725> (<https://amsacta.unibo.it/id/eprint/8725/>)

Dataset Author/s:

- [Francesco Flandi (University of Bologna)]
- [Matteo Ravaioli (University of Bologna), [0000-0001-5862-6151](https://orcid.org/0000-0001-5862-6151)]
- [Marco Rinaldo Oggioni (University of Bologna), 0000-0003-4117-793X]

Data Set Contributor/s:

- [Marco Rinaldo Oggioni (University of Bologna)]

Data Set Contact Person/s: [Add one or more contact person]

- [Marco Rinaldo Oggioni (University of Bologna), email [marcorinaldo.oggioni@unibo.it](mailto:marcorinaldo.oggioni@unibo.it)]

Data Set License:

This data set is distributed under a [this data set is distributed under a Creative Commons Attribution 4.0 International (CC BY 4.0), <https://creativecommons.org/licenses/by/4.0/>

Publication Year: [2026]

Project Info: the research leading to these results received funding from

- INF-ACT (One Health Basic and Translational Research Actions addressing Unmet Needs on Emerging Infectious Diseases), funded by European Union, NextGenerationEU programme, PNRR Extended Partnership initiative on Emerging Infectious Diseases (PNRR, Missione 4 Componente 2 - Investimento 1.3), project number PE00000007, CUP ID [B13C22002440006](https://www.opencup.gov.it/portale/web/opencup/home/progetto/-/cup/B13C22002440006), <https://www.inf-act.it/>;
- CoDiCo (Transition from asymptomatic colonization to disease by human respiratory-tract bacteria as a target for vaccines and antimicrobial therapy: The CoDiCo (colonisation to disease concepts) project), funded by the Italian Ministry of University and Research under PRIN 2020 funding programme, project number 202089LLEH;
- PRIN 2022 PNRR project “The innate immune reaction to invasive infection in human organs”, funded through the PRIN 2022 PNRR programme (National Recovery and Resilience Plan, Missione 4 Componente 2 - Investimento 1.1), financed by the European Union within the NextGenerationEU programme. Grant number P2022M8KYE.

## Data set Contents

The dataset consists of:

- 4 time lapse video files in .mp4 format
  - cells+bacteria+mannose.mp4
  - cells+bacteria.mp4
  - cells+mannose.mp4
  - cells.mp4
- 1 README file
  - Timelapse video data - Oggioni 2026_01.docx

## Abstract

In the red pulp of the human spleen, differently to equivalent anatomical sites in other animals, capsulated bacteria are captured by the CD206 mannose receptor on sinusoidal lining cells and only in a second moment delivered to CD163+ red pulp macrophages. The data deposited represent four 20 min time lapse microscopy videos of primary human spleen cell cultures containing CD163+ macrophages and CD206+ sinusoidal lining cells. The four videos show the two types of human primary cells 1) without any addition (uninfected and uninhibited control) 2) with Streptococcus pneumoniae (in green)(infected) 3) with S. pneumoniae and mannose (infected and CD206 inhibition) 4) with mannose only (not infected, but CD206 inhibition). Data are supplementary to a submitted manuscript.

## Content of the files:

- file [cells.mp4] contains [two types of human primary cells without any addition (uninfected and uninhibited control)]
- file [cells+mannose.mp4] contains [two types of human primary cells supplemented with mannose only (not infected, but CD206 inhibition)]
- file [cells+bacteria.mp] contains [two types of human primary cells infected with Streptococcus pneumoniae (in green)(infected)]
- file [cells+bacteria+mannose.mp4] contains [two types of human primary cells infected with S. pneumoniae and supplemented with mannose (infected and CD206 inhibition)]

## Data sources

[Human primary cells were obtained through clinical trial 668_2023_Sper_AOUBo 19.10.2023]

Methodology

Time-lapse confocal imaging of primary splenic cells was carried out using a Nikon A1R+ confocal laser scanning microscope with resonant scanning and a 60× objective at the microscopy facility of the University of Bologna. Cells were plated at a density of 1x10^5 per well in black, glass-bottom 96-well plates (Corning) and infected with a GFP-expressing strain of S. pneumoniae at a multiplicity of infection (MOI) of 10. Imaging took place at 37 °C over a period of approximately 20 minutes, capturing frames every 20 seconds. Image processing and cell tracking were conducted in Fiji3 using the MTrackJ plugin.
