# Supplementary material for: The mannose receptor on sinusoidal lining cells mediates two-step bacterial clearance in the human spleen
Source: Nat Commun. 2026 Apr 29;17:7595. doi: 10.1038/s41467-026-72430-8 (PMC13421466; doi:10.1038/s41467-026-72430-8)
Supplement: Supplementary file 5 — Reporting Summary [file 41467_2026_72430_MOESM5_ESM.pdf]

nature portfolio

Corresponding author(s): MR Oggioni NCOMMS-25-71126-A

Last updated by author(s): 2026_03_12

Reporting Summary

Nature Portfolio wishes to improve the reproducibility of the work that we publish. This form provides structure for consistency and transparency in reporting. For further information on Nature Portfolio policies, see our Editorial Policies and the Editorial Policy Checklist.

Statistics

For all statistical analyses, confirm that the following items are present in the figure legend, table legend, main text, or Methods section.

n/a	Confirmed	
☐	☒	The exact sample size (n) for each experimental group/condition, given as a discrete number and unit of measurement
☐	☒	A statement on whether measurements were taken from distinct samples or whether the same sample was measured repeatedly
☐	☒	The statistical test(s) used AND whether they are one- or two-sided<br>*Only common tests should be described solely by name; describe more complex techniques in the Methods section.*
☒	☐	A description of all covariates tested
☐	☒	A description of any assumptions or corrections, such as tests of normality and adjustment for multiple comparisons
☐	☒	A full description of the statistical parameters including central tendency (e.g. means) or other basic estimates (e.g. regression coefficient) AND variation (e.g. standard deviation) or associated estimates of uncertainty (e.g. confidence intervals)
☐	☒	For null hypothesis testing, the test statistic (e.g. F, t, r) with confidence intervals, effect sizes, degrees of freedom and P value noted<br>*Give P values as exact values whenever suitable.*
☒	☐	For Bayesian analysis, information on the choice of priors and Markov chain Monte Carlo settings
☒	☐	For hierarchical and complex designs, identification of the appropriate level for tests and full reporting of outcomes
☒	☐	Estimates of effect sizes (e.g. Cohen's d, Pearson's r), indicating how they were calculated

Our web collection on statistics for biologists contains articles on many of the points above.

Software and code

Policy information about availability of computer code

Data collection	data were collected using freely avaiable softwares including Phenochart, Image J, QuPath
Data analysis	data were analysed using commerical and freely avaiable softwares including GraphPad Prism, Phenochart, Image J, QuPath

For manuscripts utilizing custom algorithms or software that are central to the research but not yet described in published literature, software must be made available to editors and reviewers. We strongly encourage code deposition in a community repository (e.g. GitHub). See the Nature Portfolio guidelines for submitting code & software for further information.

Data

Policy information about availability of data

All manuscripts must include a data availability statement. This statement should provide the following information, where applicable:

- Accession codes, unique identifiers, or web links for publicly available datasets
- A description of any restrictions on data availability
- For clinical datasets or third party data, please ensure that the statement adheres to our policy

All original data and the original image files detailed in Supplementary Table 5 (List of spleen perfusion samples used to generate figures) are available through the institutional open access repository AMS Acta of the University of Bologna (https://amsacta.unibo.it/). The time-lapse files are available at https://doi.org/10.6092/unibo/amsacta/8725 and the raw image and numerical source data are available at https://doi.org/10.6092/unibo/amsacta/8855.

Research involving human participants, their data, or biological material

Policy information about studies with human participants or human data. See also policy information about sex, gender (identity/presentation), and sexual orientation and race, ethnicity and racism.

Reporting on sex and gender	The samples from human trials were transferred anonymously to the research team. No data on sex of the organ donors is known and reported.
Reporting on race, ethnicity, or other socially relevant groupings	The samples from human trials were transferred anonymously to the research team. No data on race of the organ donors is known and reported.
Population characteristics	The samples from human trials were transferred anonymously to the research team. No data on the population characteristics of the organ donors is known and reported.
Recruitment	Patients were recruited in the TIMID (organs for perfusion and cell culture) and MOSIE trial (cell cultures) and did sign informed consent. No change to the planned medical/surgical procedures for the participants did arise through the trials.
Ethics oversight	Organ samples were obtained from two separate clinical trials, the TIMID trial (REC 18/EM/0057; ClinicalTrials.gov NCT04620824) sponsored by the University of Leicester (Leicester, UK) and the MOSIE trial (CE: 668/2023/Sper/AOUBo) sponsored by the University of Bologna (Bologna Italy).

Note that full information on the approval of the study protocol must also be provided in the manuscript.

Field-specific reporting

Please select the one below that is the best fit for your research. If you are not sure, read the appropriate sections before making your selection.

☒ Life sciences ☐ Behavioural & social sciences ☐ Ecological, evolutionary & environmental sciences

For a reference copy of the document with all sections, see nature.com/documents/nr-reporting-summary-flat.pdf

Life sciences study design

All studies must disclose on these points even when the disclosure is negative.

Sample size	TIMID trial: 75 patients recruited (spleens for ex vivo perfusion and cell culture)<br>MOSIE trial: 3 patients recruited (spleen biopsy cell culture)
Data exclusions	samples were anonymised
Replication	N/A
Randomization	N/A
Blinding	N/A

Reporting for specific materials, systems and methods

We require information from authors about some types of materials, experimental systems and methods used in many studies. Here, indicate whether each material, system or method listed is relevant to your study. If you are not sure if a list item applies to your research, read the appropriate section before selecting a response.

Materials & experimental systems

n/a	Involved in the study	
☐	☒	Antibodies
☒	☐	Eukaryotic cell lines
☒	☐	Palaeontology and archaeology
☒	☐	Animals and other organisms
☐	☒	Clinical data
☒	☐	Dual use research of concern
☒	☐	Plants

Methods

n/a	Involved in the study	
☒	☐	ChIP-seq
☒	☐	Flow cytometry
☒	☐	MRI-based neuroimaging

Antibodies

Antibodies used

Primary antibodies

Antibody Specificity Target Host Conjugated Clone Working conc. Catalogue Supplier
type serum 2 Streptococcus pneumoniae Type 2 capsule Rabbit - - 1:500 16745 Statents Serum Institut
type serum 4 S. pneumoniae Type 4 capsule
Rabbit - - 1:500 16747 Statents Serum Institut
type serum 5 S. pneumoniae Type 5 capsule Rabbit - - 1:500 16748 Statents Serum Institut
group serum 6 S. pneumoniae Type 6A, 6B, 6C capsules Rabbit - - 1:500 16900 Statents Serum Institut
group serum 19 S. pneumoniae Type 19F, 19A, 19B, 19C capsules Rabbit - - 1:500 16911 Statents Serum Institut
Omni Serum S. pneumoniae All 91 serotype capsules Rabbit - - 1:500 2438 Statents Serum Institut
Anti-K2 serum Klebsiella pneumoniae K2 capsule Rabbit - - 1:500 - Statents Serum Institut
Anti-CD163 Human CD163 Mouse - EDHu-1 1:100 NB110-40686 Novus Biologicals
CD163 Antibody, anti-human, REAfinity Human, Monkey CD163 Human Recombinat Alkaline phosphatase REA812 1:100
130-112-129 Miltenyi Biotec
Human Siglec-1/CD169 Antibody Human CD169 Sheep - - 1:100 AF5197 R&D Systems
anti-human CD68 Human CD68 Mouse - PG-M1 1:100 M087601-2 Dako
CD14 antibody Human CD14 Goat - - 1:100 AHP1059 Bio-Rad
MARCO Monoclonal Antibody (PLK1)
Human MARCO Mouse - PLK1 1:100 MA5-51863 ThermoFisher
TLR2 Polyclonal Antibody
Human, Mouse TLR2 Goat - - 1:100 PA1-21611 ThermoFisher
TLR4 Polyclonal Antibody
Human, Mouse TLR4 Goat - - 1:100 PA5-142481 ThermoFisher
Human SR-AI/MSR1 Antibody Human SR-A1 Mouse - - 1:100 MAB27081 Biotechne
anti-human CD206 (MMR) Antibody Human CD206 Mouse - 15-2 1:100 321102 BioLegend
Human MMR/CD206 Antibody Human CD206 Mouse AlexaFluor 750 685641 1:100 FAB25342S-100UG R&D Systems
Human CD31/PECAM-1 Antibody Human CD31 Sheep - - 1:100 AF806 R&D Systems
Human LYVE-1 Antibody, Novus Biologicals Human LYVE-1 Mouse AlexaFluor 488 1072614 1:100 30130429 FisherScientific
THBD Mouse anti-Human, Clone: THBD/1782, Abnova™ Human CD141/THBD Mouse - THBD/1782 1:100 16063256 FisherScientific
Cleaved Caspase 3 p17 Monoclonal Antibody Human, mouse, rat Cleaved caspase-3 Mouse -
2F7B8 1:100 68773-1-IG ThermoFisher
Caspase-3 Antibody Human, mouse, rat, pig, chicken, hamster Pro and active caspase-3 Mouse AlexaFluor 488
31A1067 1:100 NB100-56708AF488 Novus Biologicals
Anti-LAMP1 antibody Human LAMP-1 Rat - 1D4B 1:100 ab25245 Abcam

Secondary antibodies

Donkey anti-rabbit IgG (H+L), Alexa Fluor 488 Rabbit Rabbit IgG Donkey AlexaFluor 488 - - A-21206 Invitrogen
Donkey anti-Mouse IgG (H+L), Secondary Antibody, Alexa Fluor 488 Mouse Mouse IgG Donkey AlexaFluor 466 - - A-21202 Invitrogen
Donkey anti-sheep IgG (H+L), Alexa Fluor 568 Sheep Sheep IgG Donkey AlexaFluor 568 - - A-21099 Invitrogen
Goat anti-Mouse IgG (H+L), Alexa Fluor 568 Mouse Mouse IgG Goat AlexaFluor 568 - - A-11004 Invitrogen
Donkey anti-Rat IgG (H+L), Alexa Fluor 568 Rat Rat IgG Donkey AlexaFluor 568 - - A-78946 Invitrogen
Donkey anti-sheep IgG (H+L), Alexa Fluor 647 Sheep Sheep IgG Donkey AlexaFluor 647 - - A-21448 Invitrogen
Donkey anti-Mouse IgG (H+L), Alexa Fluor 647 Mouse Mouse IgG Donkey AlexaFluor 647 - - A-31571 Invitrogen
Goat anti-Rat IgG (H+L), Alexa Fluor 647 Rat Rat IgG Goat AlexaFluor 647 - - A-21247 Invitrogen
Goat anti-Rabbit IgG (H+L), Secondary Antibody, Alexa Fluor 750 Rabbit Rabbit IgG Goat AlexaFluor 750 - - A-21039 Invitrogen
Goat anti-Mouse IgG (H+L), Antibody, Alexa Fluor 750 Mouse Mouse IgG Goat AlexaFluor 750 - - A-21037 Invitrogen

Validation

Antibodies were used for the species listed on the manufactrer's website. No additional validation was performed.

Clinical data

Policy information about clinical studies
All manuscripts should comply with the ICMJE guidelines for publication of clinical research and a completed CONSORT checklist must be included with all submissions.

Clinical trial registration

TIMID trial (REC 18/EM/0057; ClinicalTrials.gov NCT04620824, Leicester UK) and MOSIE trial (CE: 668/2023/Sper/AOUBo; Bologna Italy)

Study protocol

the full protocol will be made avaiable on the online repository AMS Acta of the University of Bologna

Data collection

Samples were anonymised

Outcomes

experimental data

Plants

Seed stocks	N/A
Novel plant genotypes	N/A
Authentication	N/A
