# Supplementary material for: The mannose receptor on sinusoidal lining cells mediates two-step bacterial clearance in the human spleen
Source: Nat Commun. 2026 Apr 29;17:7595. doi: 10.1038/s41467-026-72430-8 (PMC13421466; doi:10.1038/s41467-026-72430-8)
Supplement: Supplementary file 6 — Transparent Peer Review file [file 41467_2026_72430_MOESM6_ESM.pdf]

nature portfolio

Peer Review File

The Mannose Receptor on Sinusoidal Lining Cells Mediates Two-Step Bacterial Clearance in the Human Spleen

Corresponding Author: Professor Marco Rinaldo Oggioni

This file contains all reviewer reports in order by version, followed by all author rebuttals in order by version.

Version 0:

Reviewer comments:

Reviewer #1

(Remarks to the Author)
Your work offers important insights into human spleen immunobiology and is distinguished by its innovative use of ex vivo tissue from the TIMID trial. The writing is clear and we find the data of great interest, underpinned by both expertise and careful experimental design. The methods, particularly the advanced imaging and perfusion system, underpin the study's significance. However, we think there are two areas where the data could be strengthened, as well a series of minor comments.
Major Comments
1. Incomplete understanding of and lack of direct evidence for the cell handoff: The claim of pathogen handoff from sinusoidal cells to splenic macrophages is supported primarily by static imaging and co-localisation data. While these observations strongly suggest a spatial association, the authors do not provide direct evidence of dynamic transfer and how this is achieved is unclear. Establishing this mechanism is important, as it is central to the manuscript's core hypothesis and might have implications for understanding immune responses in the spleen. We understand that dynamic imaging in perfused spleens would be very difficult, although not impossible. However, handoff might be observable using cells isolated from spleens as described in the manuscript, then co-culture in tissue culture with live-cell imaging. Have such experiments been attempted? Do the team have frozen splenocytes that might allow this? Such data would move the findings beyond correlation and provide a more definitive demonstration of cellular interaction.
2. Alternative receptors and mechanistic specificity: The manuscript's conclusion regarding CD206 dependence is based largely on competitive inhibition experiments using D-mannose and, to a lesser extent, antibody blockade. While these results suggest a role for CD206, it is important to consider the possible involvement of other receptors, given the likely complexity of pathogen recognition in the spleen. The use of siRNA-mediated knockdown of CD206 in primary cultures, for example, would provide more specific mechanistic insight and help rule out compensatory pathways. However, the reliance on CD206-mediated clearance may be influenced by the opsonin-deficient nature of the system, potentially limiting canonical pathways such as MBL/CR1/CR3. Exploring additional receptor pathways would further clarify whether the observed effects are unique to CD206 or are part of a broader clearance mechanism. Investigating these pathways, or a further comment on this in the perfusion context would help to clarify whether the findings reflect physiological conditions or are model-dependent..
More minor comments
3. The mechanism described in this work 'solves' the longstanding conundrum that CD206 is not expressed on human macrophages, in contrast to mice. It would help the generalisability of the conclusions presented here if some observations on murine splenic clearance were included. This need not be comprehensive.
4. We would be interested in some more comment on the roles of the two populations of macrophages described here. Is the role of CD163+ macrophages mainly bacterial clearance? Is the major role of the CD169+ macs presenting antigen to the white pulp? Do the authors think that CD206+ cells transfer bacteria to both types directly or is there macrophage to macrophage transfer? (also see comments below)
In summary, the manuscript is well written, engaging, and demonstrates methodological innovation, particularly through the use of human spleen tissue from the TIMID trial. This approach provides valuable translational relevance and represents a significant strength of the study. Addressing the outlined critiques, most notably by providing direct mechanistic evidence and considering alternative receptor pathways, would substantially enhance the impact and clarity of the findings.

Comments on details / copy editing
Abstract: the spleen is not the main organ involved in invasive bacterial infection, this should be restricted to septicaemias.

There are numerous invasive more localised infections. We don't understand what the word translational means here. 'These data change completely' is hyperbolic. Please tone down to something like 'provide new insights'. They don't show our previous understanding was wrong, just that it was poor. I agree there are implications for vaccine efficacy and anti-infective strategies, but disagree these are 'profound'.
Introduction:
'sinusoidal lining littoral cells, which make up the structure of the human spleen red pulp' implies there are no other cell types in the red pulp, which is untrue.
is a key molecule involved in the binding to bacteria………'the' is redundant here, it is not specifying anything.
'endocytosis of the receptor is associated to the uptake and the subsequent killing of the bacteria inside the macrophage compartments' should be 'endocytosis of the receptor is associated with uptake and subsequent killing of bacteria inside macrophage compartments'.
The species specific differences in CD206 expression in various animals should be highlighted when introducing the various animal models.
porcine spleen perfusion, HAVE revealed
However, mannose receptor expression on sinusoidal endothelial cells BUT not macrophages raiseS questionS about pathogen clearance mechanisms.
'the unique translational platform'. It's not unique – others have used such systems.
Cellular marker distribution in the human spleen
Fig 1: CD206 area looks appreciably higher than CD163, yet similar on graph. Please explain.
'different infection stages showed no variation'. There is variability, just relatively low. Note: 'variation is the occurrence of differences, whether natural or manufactured, while variability is the statistical measure of this spread or dispersion within a dataset'.
'first passage through the splenic open circulation'. Why not second, third or subsequent passage?
Contribution of the mannose receptor on sinusoids to bacterial clearance in the human spleen
despite the spleen being the primary site for pneumococcal clearance FROM THE BLOOD.
instead, BACTERIA instead accumulated progressively in both perfusate and tissue
'These results identify the carbohydrate-binding activity of CD206 on sinusoidal lining cells as an essential initial step in pathogen clearance'. No these results don't – they identify mannose binding lectin receptors as being important. There may be other receptors other than CD206 that are important (see above). Later anti CD206 antibodies reduced binding in vitro (Fig 4) and this would be the place in the paper to stress the primacy of CD206.
F2 – would it have been possible to perform sequential perfusions with mannose?
F3 – clearly complex. Is there a better way of adjusting for specificity of coincidental staining, that takes into account total areas occupied?
F3B2 shows some bacteria not in contact with CD206. Do the authors have any comment?
What is the explanation for the increase in CD206 with bacteria at 2 and 5 hours in 3A?
3D+E changes need more comment.
F4B bacteria still bind to CD163 cells, not dramatically different to CD206
+ cells – needs a bit more comment. Why does mannose have such a small effect? Dose-response?
Mechanism of pathogen clearance by both CD163+ and of CD169+ macrophages
Only slightly fewer bacteria are associated with CD169+ than CD163+ cells, despite the former's much lower abundance. How did the bacteria get there.? Looking at F1A/B, most of the CD206+ and CD13+ cells are a long way from the few CD169+ cells. How do the authors postulate bacteria are communicated? Or is there a hold up in CD169+ cells, thereby inflating bacterial counts?
'reflecting an ability to accelerate engagement when genuine pathogens are detected'. Do the authors really mean this, which implies presence of bacteria increases uptake rates? I suspect they actually mean that bacteria are more efficiently recognised than beads.
'While these data do not allow quantification of the relative bactericidal contributions of each macrophage subset, they indicate that both CD163+ and CD169+ macrophages consistently retained similar bacterial loads over time, implying a comparable role in pneumococcal capture and clearance'. The authors correctly acknowledge that these snapshots do not allow measurement of bacterial killing ie measurement of flux. They also do not allow measurement of bacterial retention. The phrase 'comparable role' is ill-defined. I suspect that the role of the more numerous CD163+ cells is bulk clearance of bacteria, while the CD169+ cells contribute in minor way to clearance, but their proximity adjacent to the lymphocytes in the white pulp implies an in important role in antigen presentation for later recruitment of adaptive immunity.
Re LAMP1 quantitation. Are the authors postulating an increase in translated protein or detection of protein as lysosomes are activated?
This corresponds to roughly 7x107 to 3x108 apoptotic cells per spleen – surely this corresponds to 1.10^8? Where does this confidence interval come from?
'This correspondence suggests a direct link between the quantity of phagocytosed bacteria and the induction of macrophage apoptosis'. This conclusion would be better supported by a time course of apoptosis versus bacterial numbers.
efficiency even, as individual macrophages are lost – this comma impedes understanding.

Supplemental
Methods not Methodology
'M.R. Oggioni as scientific responsible' – responsible scientist?
Statents Serum Institut – Statens not Statents.
Fig S1 - Cellular markers expression in the human spleen – either Cellular marker expression in the human spleen or Expression of cellular markers in the human spleen (latter better).

Reviewer #2

(Remarks to the Author)
I co-reviewed this manuscript with one of the reviewers who provided the listed reports. This is part of the Nature Communications initiative to facilitate training in peer review and to provide appropriate recognition for Early Career Researchers who co-review manuscripts.

Reviewer #3

(Remarks to the Author)
The paper is very interesting and the idea of CD206+ cells acting as a capture cell for the bacteria is novel, I think the authors should be more concise in their phrasing especially in their figure legends and there is a lack of consistency in data reporting. In my opinion the first two paragraphs are for the supplementary and although the mannose effect is very interesting there is a lack of control (mannose treated non-infected spleens) on the host response to mannose. Additionally, there are no mechanistic insight into how the interaction between CD206+ cells and macrophage is facilitated and although I believe that macrophages do phagocytosis the bacteria, figure 5 is not novel. Although the model and the finding is exciting, I believe it lacks some experiments (see bottom of my comments).

MAJOR
Cellular marker distribution in the human spleen. Is the compartmentalized immune architecture differing between macrophages in the blood and macrophages in the spleen? This analysis reveals a CD206 expression among macrophages, with the CD206+ cells in an ideal position to uptake and eliminate microbes. Can the CD206+ cells be sorted and cultured in vitro for phagocytosis assays to directly proof this hypothesis?
Ex vivo organ perfusion. Was the >90% bacterial load removed exclusively by CD206+ cells? Without this piece of information, the results of this section are quite redundant. Can AMR pneumococcal strains be removed as well? Or pneumococcal strains with hyper capsulation, such as serotype 1?
Contribution of the mannose receptor on sinusoids to bacterial clearance in the human spleen. The sentence “In the human spleen, however, macrophages lack CD206 entirely” goes totally in contrast to the results described in the section “Cellular marker distribution in the human spleen” in which some macrophages are classified as “CD206+”. Quite surprising was the fact that, considering mannose receptor a ligand for pneumolysin (Subramanian et al, Nat Microbiol, 2020), the authors have not investigated the pneumolysin-mannose receptor binding as triggering factor for macrophage clearance activity; on the other hand, authors seem to focus primarly on capsulation. In vitro, this can be easily tested by pretreating macrophages with either sub-lytic concentration of pneumolysin or polysaccharide capsule, and assess the effect on phagocytic capacity. In addition, the results shown by the authors in Fig 4 should be performed with pneumolysin-deficient mutant to assess the role of pneumolysin-mannose receptor binding in macrophage response.
Mechanism of pathogen clearance by both CD163+ and of CD169+ macrophages. Firstly, this section should be directly linked/merged with the section “Ex vivo organ perfusion”. This section seems to highlight the strong bacterial clearance activity performed by the less abundant CD163+ and CD169+ cells in the spleen, even though in the section “Cellular marker distribution in the human spleen” authors have speculated that the CD206+ cells should be the ones with higher capability for bacterial clearance. Clarity on this point should be given.
To assess the bacterial clearance capacity of CD163+ and CD169+ cells, compared to CD206+ cells, cells should be sorted, cultured and used for phagocytosis assays in vitro to measure bacterial killing ratio. Microscopy experiments can only provide a partial quantification of what is happening in an whole human organ.
In my opinion, concerning bacterial clearance, more relevant than caspase-3, authors should assess the lysosomal activity inside the splenic macrophages (lysosome intracellular activity) which is a more direct readout of the active phagocytosis activity inside lysosomes.

Additionally:
- The authors used mannose to inhibit the mannose receptor, mannose is a well known to cause an anti-inflammatory effect. E.g Torretta S, Scagliola A, Ricci L, Mainini F, Di Marco S, Cuccovillo I, et al. D-Mannose Suppresses Macrophage IL-1beta Production. Nat Commun (2020) 11(1):6343. doi: 10.1038/s41467-020-20164-6. Additonally cytokine data indicate a difference between mannose treated spleens and infected spleens (reduced IL-6, Il-1B)
- The authors should design experiments to assess if there is an effect of mannose on the resident immune population
- The paper lacks a discussion paragraph on the limitation of the study
- In some graphs limited info on n numbers, what each symbol represent, if the data is presented as SD or SEM

MINOR
No information is given on n numbers in each graph, statistical test used, nor if the bars represent SD (assumed based on text), or SM, the authors should provide this information
Information on statistical test is needed, has Dunn multiple comparison always been performed? Is it an unpaired t-test? One-tailed or two-tailed? Was the data tested for normal distribution?
Has imaging analysis been performed blinded?
Abstract: Lacks scientific phrasing, e.g labour
Results:
Line: 108: Can the authors clarify if there are different populations of sinusoid lining littoral cells , since most literature describe these cells as CD31+, but the authors comment that they do not express this
Line 108. The authors don’t show the co-localization of the markers to identify their cell population (e.g CD68 + CD163 and CD68 and CD169).
Supplementary figure 1 D1D2. There are some data points missing for HSP3, HSP50, have experiments not been

performed for these? Additionally mannose pre-treatment seems to have an effect on the density of CD163+ cells
LINE 118: This statement seems to not be supported by S1D1-S1D2, as there seems to be an increase in both D163+ and CD206+ upon infection, alternatively no info is given if these data has been statistically tested
Line140: What is the rational for using such a high CFU dose?
Figure 2: Please adjust the Y-scale, so the viewer can observe the data points in A1, A2, B1, B2, D1, D2, E1, E2. Its very difficult to analysis the differences between the strains and its elimination
Line 143: What about bacterial signal onto CD206+ cells?
Line 160: Although the confocal images demonstrate the authors statement, this type of statement should be supported by literature or a Flow cytometric analysis on the macrophage population. Additionally, the images seems overexposed, especially in the red channel.
Line 174: The effect of mannose on the bacteria should be assessed with a growth curve, to confirm that the bacteria has a normal phenotype
Figure 3A1, A2. The authors have a much higher n number for mannose treated groups, this could significantly drag the data. Also the authors should state what each symbol represent since each figure is only quantified based on 2 spleen.
Figure 3D1,D2 why are there data points missing in D2 0.5 grey bar compared to D1?
Figure 3E1, the data is dragged by one outlier in no treatment group, has the data been analysed for outliers?, without this outlier, the bars look identical. The statement of CD169+ macrophages should be modified.
Line: 197:Have the authors addressed if there are dendritic cells in their culture, especially since these can express cd206.
Figure A2. The image is overexposed, better quality pictures should be provided
Figure 4F, The data should be presented as CFU/ml or bacterial signal, not as % of dose
Figure 5A. where is the quantification for time point 0? Does the staining not give any background?
Figure 5B1 and B3, this is a very weak association and no information is given if this association has been tested statistically
General comment:
If the CD206+ cells present the bacteria to macrophages, then are they also degraded during the phagocytosis of the bacterium?
Line272-278: These images should be included in the supplementary, and why data not shown?
LINE 284: The data does not support this statement: caspase-3 is also increasing in control spleens, to assess if there is an increase in caspase-3 upon infection the data should be compared to control spleen caspase-3 levels and a two-way ANOVA should be performed.
Line 291-295: Where does the 0.65% number come from, the graphs indicate a 10% of CD163+ cells having caspase-3 signalling, how does that give 0.65% in the text? The authors should clarify
Additional experiments:
1. Caspase-3 on CD206+ cells
2. Mannose effect on the immune system itself without an infection
3. Primary cell culture purity (DCs)
4. Further proof or mechanistic explanation between CD206+ and macrophage interaction for bacterial clearance should be performed.
5. To fully illustrate that the capsule is the main determinant of CD206+ capturing the bacteria, experiments with a pneumococcal strain that is hypercapsualted (serotype 2 for instance) should be compared to lower capsule containing strains.
6. Address if a Ply mutant strain would bind the mannose-receptor of CD206+ cells

Reviewer #4

(Remarks to the Author)
Interesting data identifying a novel mechanism by which encapsulated S. pneumoniae and probably other bacterial pathogens are cleared by the spleen. This is clearly an area of high importance and identifying a novel clearance mechanism is therefore of very high interest. Obtaining these data requires the ex vivo spleen model and is therefore extremely difficult.

Major issues/questions:

The two biggest issues that need addressing via text is (a) how does the mannose receptor recognise such a broad range of capsule structures not all of which contain mannose? And (b) how does the proposed handing on mechanism from MR206 expressing sinusoidal cells to other splenic macrophage populations actually work as my concept of splenic macrophage populations is that they are fixed in the tissue rather than mobile? I don’t think we need more data on this at this stage but some discussion of these points would be important even if it is essential ‘this is unclear….’

The data on equal speed of clearance of different serotypes is interesting but (a) it does look like there could be differences at a the later timepoints that are not significant due to power issues; and (b) it is probably too far a leap to say these data for five serotypes means all serotypes are cleared equally well.

Lines 223 to 269, and 291 to 341 seem to be discussion but are not subtitled as such; in fact there is no formal discussion section and the results and not labelled as results and discussion. Also lines 315 to 341 repeat earlier text. A separate discussion section would be helpful and allowed by Nat Comms. Any discussion needs to address how these results fit with the existing fairly extensive data on splenic clearance obtained using mouse models etc and whether there are major differences between species.

Line 294 correspondence between apoptotic cell numbers and bacterial inoculum; what were the results when the lower inoculum was used? Without these data I think the authors should be wary of drawing too much of a conclusion from the similar numbers.

Figure 5: it looks like in panels E to F that uninfected spleens have raised apoptotic markers at early timepoints including in some data higher than infected spleens at the same time point; this is not mentioned in the text and needs to be. The persistence of higher levels of apoptotic cells in the infected spleens then probably becomes the main point of these data, which is slightly different to how it is written at present

Minor issues

Figure 1 legend is a bit light on detail; for example, what panels D to F are actually showing is not that clear. I think some measure of immunofluorescence per mm2
spleen?

Line 141: the meaning of 'of five 140 equally counted serotypes' is not that clear; equal inoculum sizes?

Figure 2 panels AB E D I would favour reducing the y axis so the curves can be seen rather than keep the y axis spread the same across all panels

Lines 157-64 largely repeat introduction text and can be reduced

Lines 174-76 the authors could just grow the bugs in the perfusate +/- mannose to prove the point that mannose does not increase S. pneumoniae replication?

Figure 4; did the authors identify any CD169 cells?

Figure 5 panel C; black bars are the grey columns? And the five different sets of grey bars represent five different spleens? If correct, this needs stating in the legend and the spleen donor number added to the x axis. If each grey section is data from a separate spleen in panel C, are the data in panels D to F poole from multiple spleens or just one?? And , if I have completely misinterpreted these panels then please make it much clearer what they represent. Title for this figure; it does really contain bactericidal data? Replace with something about apoptosis in the title to the legend?

Reviewer #5

(Remarks to the Author)
I co-reviewed this manuscript with one of the reviewers who provided the listed reports. This is part of the Nature Communications initiative to facilitate training in peer review and to provide appropriate recognition for Early Career Researchers who co-review manuscripts.

Version 1:

Reviewer comments:

Reviewer #1

(Remarks to the Author)
I thank the authors for answering my questions in a comprehensive fashion. I am particularly gratified that the experiments I suggested yielded data of great interest and have added to the manuscript. I think the paper should now be acceptable for publication in Nature Communications.

Reviewer #2

(Remarks to the Author)
I co-reviewed this manuscript with one of the reviewers who provided the listed reports. This is part of the Nature Communications initiative to facilitate training in peer review and to provide appropriate recognition for Early Career Researchers who co-review manuscripts.

Reviewer #3

(Remarks to the Author)
I appreciate the effort of the authors to address most of the comments. However, there are a few things they disagree on, such as:
1.Authors state that the pictures needs to be overexposed in order to be viewed on paper, but overexposure is a major source of artifacts in microscopy.
2. Flow cytometry cannot be done according to the authors, which I do not see any valid reason why.
3. Reporting of bacterial CFU, which is very important.

Reviewer #4

(Remarks to the Author)
I have no major queries about the resubmitted version
a few minor issues
Line 74: the full use of bacterial names after first use needs revising to the abbreviated form
line 302 missing a 'the' - '...the presence CR-301 ligands has been linked to receptor-mediated antigen transfer...'
Figure 2 panel C1 is labelled No mannose' but I think is with mannose?

Reviewer #5

(Remarks to the Author)
I co-reviewed this manuscript with one of the reviewers who provided the listed reports. This is part of the Nature Communications initiative to facilitate training in peer review and to provide appropriate recognition for Early Career Researchers who co-review manuscripts.

Open Access This Peer Review File is licensed under a Creative Commons Attribution 4.0 International License, which permits use, sharing, adaptation, distribution and reproduction in any medium or format, as long as you give appropriate credit to the original author(s) and the source, provide a link to the Creative Commons license, and indicate if changes were made.
In cases where reviewers are anonymous, credit should be given to 'Anonymous Referee' and the source.
The images or other third party material in this Peer Review File are included in the article's Creative Commons license, unless indicated otherwise in a credit line to the material. If material is not included in the article's Creative Commons license and your intended use is not permitted by statutory regulation or exceeds the permitted use, you will need to obtain permission directly from the copyright holder.
To view a copy of this license, visit https://creativecommons.org/licenses/by/4.0/

Reviewer #1 (Remarks to the Author):

Your work offers important insights into human spleen immunobiology and is distinguished by its innovative use of ex vivo tissue from the TIMID trial. The writing is clear and we find the data of great interest, underpinned by both expertise and careful experimental design. The methods, particularly the advanced imaging and perfusion system, underpin the study's significance. However, we think there are two areas where the data could be strengthened, as well a series of minor comments.
REPLY: We thank the reviewer for this positive feedback and have tried to address their valuable observations.

Major Comments
1. Incomplete understanding of and lack of direct evidence for the cell handoff: The claim of pathogen handoff from sinusoidal cells to splenic macrophages is supported primarily by static imaging and co-localisation data. While these observations strongly suggest a spatial association, the authors do not provide direct evidence of dynamic transfer and how this is achieved is unclear. Establishing this mechanism is important, as it is central to the manuscript's core hypothesis and might have implications for understanding immune responses in the spleen. We understand that dynamic imaging in perfused spleens would be very difficult, although not impossible. However, handoff might be observable using cells isolated from spleens as described in the manuscript, then co-culture in tissue culture with live-cell imaging. Have such experiments been attempted? Do the team have frozen splenocytes that might allow this? Such data would move the findings beyond correlation and provide a more definitive demonstration of cellular interaction.
REPLY: We thank the referee for urging us to carry out these experiments, as results were astonishing. Using fresh primary cultures we performed time-lapse videos with GFP expressing pneumococci with/without bacteria and mannose. In agreement with our bacteria handling hypothesis, data indicate that macrophages near to sinusoids carry 8 times more bacteria compared to macrophages located farther from sinusoids, and that macrophage association to bacteria is drastically decreased in presence of mannose (Fig. 5E). Unexpected motility analysis revealed that macrophage movement is not influenced by either the presence of bacteria or contact with sinusoidal cells (Supplementary Figure 4). Mannose also does not influence motility when macrophages are far from sinusoids, but macrophages become immotile in the presence of mannose when in contact with sinusoids, implying that blocking the CD206 receptor has a direct effect on sinusoid/macrophage interactions (Fig 5E and Supplementary Figure 4).

2. Alternative receptors and mechanistic specificity: The manuscript's conclusion regarding CD206 dependence is based largely on competitive inhibition experiments using D-mannose and, to a lesser extent, antibody blockade. While these results suggest a role for CD206, it is important to consider the possible involvement of other receptors, given the likely complexity of pathogen recognition in the spleen. The use of siRNA-mediated knockdown of CD206 in primary cultures, for example, would provide more specific mechanistic insight and help rule out compensatory pathways. However, the reliance on CD206-mediated clearance may be influenced by the opsonin-deficient nature of the system, potentially limiting canonical pathways such as MBL/CR1/CR3. Exploring additional receptor pathways would further clarify whether the observed effects are unique to CD206 or are part of a broader clearance mechanism. Investigating these pathways or a further comment on this in the perfusion context

would help to clarify whether the findings reflect physiological conditions or are model-dependent.

REPLY: We have tested a substantial set of receptors and enzymes potentially involved in bacterial uptake and killing including CD14, CD68, CD163, CD169, MARCO, TLR2, TLR4, SR-A1 and the caspase inhibitor Z-DVED-FMK on macrophages and CD206, LYVE-1, CD31 and CD141 on sinusoidal lining cells to characterise their involvement in the observed phenotypes. These data indicate that phagocytosis in primary cultures is affected exclusively by inhibition of CD206 on sinusoidal lining cells and MARCO on macrophages (Fig. 5D). As the observed bactericidal activity in our in vitro primary cultures, in contrast to the organ perfusion, did neither involve complement nor antibodies, we did not test impact of complement or Fc receptors. Surprisingly, none of the receptor inhibitions did reduce the highly efficient killing of non-capsulated bacteria (in absence of complement and antibody) for which we still have no explanation except for multiple receptors potentially being involved in the uptake of non-encapsulated bacteria.

More minor comments

3. The mechanism described in this work ‘solves’ the longstanding conundrum that CD206 is not expressed on human macrophages, in contrast to mice. It would help the generalisability of the conclusions presented here if some observations on murine splenic clearance were included. This need not be comprehensive.

REPLY: Primary murine spleen cell cultures have been tested for phagocytosis inhibition and here both the scavenging receptor CD68 and the mannose receptor CD206, both expressed in mice by red pulp macrophages, were found involved in bactericidal activity (Supplementary Figure 3 B).

4. We would be interested in some more comment on the roles of the two populations of macrophages described here. Is the role of CD163+ macrophages mainly bacterial clearance? Is the major role of the CD169+ macs presenting antigen to the white pulp? Do the authors think that CD206+ cells transfer bacteria to both types directly or is there macrophage to macrophage transfer? (also see comments below)

REPLY: The referee here identifies an essential issue not addressed in this work, which focusses exclusively on the task of pathogen removal. Using Qupath for cell segmentation, it removed also the only observed difference reported between CD163+ and CD169+ cells in this paper documenting that both macrophage cell types show comparable level of caspase-3 activation. We have addressed the issue on the effective role of the two populations shortly in the discussion. We are currently running 10x spatial sequencing hoping to gain form this analysis indications on markers or molecular pathways characterising the difference between the two cell types.

In summary, the manuscript is well written, engaging, and demonstrates methodological innovation, particularly through the use of human spleen tissue from the TIMID trial. This approach provides valuable translational relevance and represents a significant strength of the study. Addressing the outlined critiques, most notably by providing direct mechanistic evidence and considering alternative receptor pathways, would substantially enhance the impact and clarity of the findings.

REPLY: We agree with the referee and have addressed this issue by identifying the receptor on human splenic macrophages and importantly showing evidence for changes in macrophage

motility next to sinusoidal lining cells in presence of CD206 inhibition (Fig. 5). While we have still no explanation for this variation in macrophage motility when blocking CD206 we feel that these two datasets substantially extend our observations and fully support the hypotheses raised in this paper.

Comments on details / copy editing
Abstract: the spleen is not the main organ involved in invasive bacterial infection; this should be restricted to septicaemias. There are numerous invasive more localised infections. We don't understand what the word translational means here. 'These data change completely' is hyperbolic. Please tone down to something like 'provide new insights. They don't show our previous understanding was wrong, just that it was poor. I agree there are implications for vaccine efficacy and anti-infective strategies but disagree these are 'profound'.
REPLY: we thank the referee for this evaluation. We have revised the wording.

Introduction:
'sinusoidal lining littoral cells, which make up the structure of the human spleen red pulp' implies there are no other cell types in the red pulp, which is untrue.
REPLY: We thank the referee for this comment and have modified the wording to clarify that sinusoidal lining littoral cells are an important component of the human spleen red pulp, without implying that they are the only cell type present.

is a key molecule involved in the binding to bacteria.........'the' is redundant here, it is not specifying anything.
REPLY: We have modified this

'endocytosis of the receptor is associated to the uptake and the subsequent killing of the bacteria inside the macrophage compartments' should be 'endocytosis of the receptor is associated with uptake and subsequent killing of bacteria inside macrophage compartments'.
REPLY: We have modified this

The species specific differences in CD206 expression in various animals should be highlighted when introducing the various animal models.
REPLY: We have added phagocytosis inhibition in primary murine macrophages showing inhibition by both CD68 and CD206 antibodies (Supplementary Figure 3 B) and added the information in the results emphasising that that both markers are expressed on murine red pulp macrophages.

porcine spleen perfusion, HAVE revealed
REPLY: We have modified this

However, mannose receptor expression on sinusoidal endothelial cells BUT not macrophages raiseS questionS about pathogen clearance mechanisms.
REPLY: We have modified this

'the unique translational platform'. It's not unique – others have used such systems. Cellular marker distribution in the human spleen
REPLY: We have modified this

Fig 1: CD206 area looks appreciably higher than CD163, yet similar on graph. Please explain. ‘different infection stages showed no variation’. There is variability, just relatively low. Note: ‘variation is the occurrence of differences, whether natural or manufactured, while variability is the statistical measure of this spread or dispersion within a dataset’.
REPLY: Probably the main issue in Figure 1 is the log scale, which was chosen to display all three markers on the same scale. A representation showing more clearly the differences between CD206 and CD163 is shown in Supplementary Figure 1 where we use a linear scale in panels A and B.

‘first passage through the splenic open circulation’. Why not second, third or subsequent passage?
REPLY: we have modified this

Contribution of the mannose receptor on sinusoids to bacterial clearance in the human spleen despite the spleen being the primary site for pneumococcal clearance FROM THE BLOOD.
REPLY: we have modified this

instead, BACTERIA instead accumulated progressively in both perfusate and tissue
REPLY: we have modified this

‘These results identify the carbohydrate-binding activity of CD206 on sinusoidal lining cells as an essential initial step in pathogen clearance’. No these results don’t – they identify mannose binding lectin receptors as being important. There may be other receptors other than CD206 that are important (see above). Later anti CD206 antibodies reduced binding in vitro (Fig 4) and this would be the place in the paper to stress the primacy of CD206.
REPLY: We thank the referee and we have now also tested LYVE-1, CD141 and CD31. Indeed, from these sinusoidal markers only CD206 inhibits bacterial killing. We have also tested macrophage markers and identified MARCO as the only receptor whose inhibition blocks bactericidal action of splenic macrophages (Fig. 5D1). Perhaps the most intriguing evidence is the nearly complete inhibition of macrophage motility next to sinusoids when the CD206 is blocked by mannose. This is independent of the presence of bacteria and also does not occur in macrophages nearby but further from sinusoidal cells in the same time lapse microscopy field. These data indicate that there is intimate mannose-dependent cross-talk between both cells which we will aim to address in further work (Fig 5E and Supplementary Figure 4).

F2 – would it have been possible to perform sequential perfusions with mannose?
REPLY: during organ ex vivo perfusion we do not change medium, but in primary cell cultures we first expose cells to antibody +/- mannose, then wash and only then challenged with bacteria. This indicates that at least in the short term, mannose binding is stable and hinders subsequent biding by bacteria even in absence of mannose. We have clarified in both the methods sections and main text.

F3 – clearly complex. Is there a better way of adjusting for specificity of coincidental staining, that takes into account total areas occupied?
REPLY: Using Qupath virtual cell boundaries are drawn and cells, instead of areas are scored. We have added a panel in Supplementary Figure 3 D3 showing that this analysis normalises the differences between CD163 and CD169 macrophages relative to caspase activation.

Importantly the reanalysis of data in this panel does not alter the overall message of an increase of caspase-3 activation at 1h.

F3B2 shows some bacteria not in contact with CD206. Do the authors have any comment? What is the explanation for the increase in CD206 with bacteria at 2 and 5 hours in 3A? 3D+E changes need more comment.
REPLY: F3B2) The bacteria that do not appear in contact with CD206+ cells (red) are instead found interacting with CD163+ red pulp macrophages (magenta). This potentially highlights the close interplay between CD206+ cells and RPMs during bacterial engagement and subsequent killing. Fig. 3A) The increased colocalization of bacteria with CD206+ cells at 2- and 5-hours post-infection may simply reflect the higher bacterial load in both the perfusion liquid and the tissue during mannose perfusion. Our imaging approach cannot distinguish between the mere spatial overlap of bacterial and CD206 signals and true bacterial binding to the CD206 receptor. However, the greater number of bacteria observed at later time points during mannose perfusion (Fig. 2C1–C2; F1–F2), indicate that bacteria are not cleared efficiently in the presence of mannose, suggests that these bacteria are not actually bound by CD206 and subsequently transferred to macrophages. This interpretation is further supported by the decrease in bacteria-macrophages colocalizations shown in Fig. 3D1–3E2.

F4B bacteria still bind to CD163 cells, not dramatically different to CD206+ cells – needs a bit more comment. Why does mannose have such a small effect? Dose-response?
REPLY: For sinusoidal cells, CD206 appears to be the only receptor through which the binding of bacteria can occur, as inhibition of other sinusoidal markers (LYVE-1, CD141 and CD31) had no effect on bacterial clearance. We demonstrated that the receptor responsible for bacterial uptake in RPMs is the scavenger receptor MARCO (Fig. 5D1). The unchanged number of bacteria–macrophage associations during mannose treatment may be explained by the continued functionality of MARCO, despite inhibition of sinusoidal CD206. The simultaneous presence of functional CD206 (in RPM-adjacent sinusoidal cells) and MARCO likely helps to maintain a fast and efficient bacterial killing by RPMs. Additionally, while macrophages and sinusoidal cells are anatomically constrained to remain in close proximity within the intact organ, this spatial relationship is difficult to preserve in a homogenized primary cell culture. Thus, the lack of an effect on macrophages in culture may reflect the presence of macrophages that are no longer closely associated with CD206+ sinusoidal cells. To the contrary the timelapse analyses (Fig. 5E and Supplementary Figure 4) clearly show less bacteria on macrophages in presence of mannose. We do not envisage that these differences are representing a contrast, they appear only to show different situations with extreme high 3D cell densities and a MOI of 0.02 in the organ and low cell 2D densities with an MOI of 10 in the chamber slide – a dramatic change in infection conditions.

Mechanism of pathogen clearance by both CD163+ and of CD169+ macrophages Only slightly fewer bacteria are associated with CD169+ than CD163+ cells, despite the former's much lower abundance. How did the bacteria get there.? Looking at F1A/B, most of the CD206+ and CD163+ cells are a long way from the few CD169+ cells. How do the authors postulate bacteria are communicated? Or is there a hold up in CD169+ cells, thereby inflating bacterial counts?

REPLY: CD169+ cells are 30-50 times less abundant than CD163+ cells in the red pulp. This explains why the contribution of CD169+ to pathogen clearance is at background levels and not detectable in whole spleens or cell cultures.
Having stated this, CD169+ cells, as they surround perifollicular arterioles, may either sample bacteria directly from the bloodstream or, like meningeal periarteriolar macrophages, receive bacteria after transcytosis through CD31+/PECAM+ endothelial cells (see papers of Federico Iovino; e.g. doi: 10.1093/infdis/jiy193). The higher association of bacteria to CD169+ cells compared to CD163+ cells is dependent on the extent of staining likely due to marker distribution on the respective cells. When using Qupath to predict cell area, instead of using raw staining values, the difference in the association of CD163+ and CD169+ to activated-caspase-3 disappeared (Supplementary Figure 3 D3) indicating incomplete cell staining by the CD163 antibody as likely cause of the observed difference. Importantly, the increase at 1h of caspase-3 activation is still clearly visible in both cell types.

'reflecting an ability to accelerate engagement when genuine pathogens are detected'. Do the authors really mean this, which implies presence of bacteria increases uptake rates? I suspect they actually mean that bacteria are more efficiently recognised than beads. 'While these data do not allow quantification of the relative bactericidal contributions of each macrophage subset, they indicate that both CD163+ and CD169+ macrophages consistently retained similar bacterial loads over time, implying a comparable role in pneumococcal capture and clearance'. The authors correctly acknowledge that these snapshots do not allow measurement of bacterial killing ie measurement of flux. They also do not allow measurement of bacterial retention. The phrase 'comparable role' is ill-defined. I suspect that the role of the more numerous CD163+ cells is bulk clearance of bacteria, while the CD169+ cells contribute in minor way to clearance, but their proximity adjacent to the lymphocytes in the white pulp implies an in important role in antigen presentation for later recruitment of adaptive immunity.
REPLY: We thank the referee for the insight and fully support their view. We have checked the main text for clarity. In line with the referee's suggestions, our plans for the future are to also explore the intracellular replication, but while 1/500 cells in the spleen may be involved in killing of pneumo, only 1/50.000 may be permissive to intracellular replication with numbers for CD169+ cells even 10 time lower. We anticipate that elucidating the molecular mechanisms underlying such rare events will be challenging and may require some luck. We agree that in this context the potential antigen presentation/transfer will have to be explored.

Re LAMP1 quantitation. Are the authors postulating an increase in translated protein or detection of protein as lysosomes are activated?
REPLY: We postulate detection of increased protein probably because of enhanced lysosomal activity .

This corresponds to roughly 7x107 to 3x108 apoptotic cells per spleen – surely this corresponds to 1.10^8? Where does this confidence interval come from? 'This correspondence suggests a direct link between the quantity of phagocytosed bacteria and the induction of macrophage apoptosis'. This conclusion would be better supported by a time course of apoptosis versus bacterial numbers.
REPLY: We have revised the text accordingly. In addition, we have, as suggested, quantified caspase-3 activation in staining of biopsies from the lower dose infection and did detect 10-fold less caspase-activation which is near/at the background level (Supplementary Figure 3

D4). While this is not a demonstration of any causative link, it is consistent with the narrative of the paper.

efficiency even, as individual macrophages are lost – this comma impedes understanding. REPLY: The sentence was removed.

Supplemental Methods not Methodology, 'M.R. Oggioni as scientific responsible' – responsible scientist? Statents Serum Institut – Statens not Statents. Fig S1 - Cellular markers expression in the human spleen – either Cellular marker expression in the human spleen or Expression of cellular markers in the human spleen (latter better).
REPLY: we have modified this.

Reviewer #3 (Remarks to the Author):

The paper is very interesting and the idea of CD206+ cells acting as a capture cell for the bacteria is novel, I think the authors should be more concise in their phrasing especially in their figure legends and there is a lack of consistency in data reporting. In my opinion the first two paragraphs are for the supplementary and although the mannose effect is very interesting there is a lack of control (mannose treated non-infected spleens) on the host response to mannose. Additionally, there are no mechanistic insight into how the interaction between CD206+ cells and macrophage is facilitated and although I believe that macrophages do phagocytosis the bacteria, figure 5 is not novel. Although the model and the finding is exciting, I believe it lacks some experiments (see bottom of my comments).
REPLY: We thank the reviewer for their thoughtful comments. We have addressed several points in the revised manuscript as follows:

1) We have now defined the MARCO receptor on macrophages as being involved in the clearance process, but most importantly performed time-lapse microscopy, which shows that mannose has no effect at all on macrophage mobility when macrophages are not in contact with sinusoids (Fig. 5D 5E Supplementary Figure 4). The time lapse data very intriguingly indicate that macrophages stop completely moving when in contact with sinusoids in presence of mannose (Figure 5E, Supplementary Figure 4). This implies most likely a direct interaction between sinusoids and macrophages via the CD206 receptor. We feel that these data strengthen the data and hypothesis of the paper.
2) We have shown mannose inhibition of bactericidal activity in primary human splenic cell cultures occurs in a wide range on pneumococcal capsule types and appears to be independent from presence of pneumolysin (Supplementary Figure 3 A).
3) We have revised the manuscript and figure legends for conciseness and clarity

MAJOR
Cellular marker distribution in the human spleen. Is the compartmentalized immune architecture differing between macrophages in the blood and macrophages in the spleen? This

analysis reveals a CD206 expression among macrophages, with the CD206+ cells in an ideal position to uptake and eliminate microbes. Can the CD206+ cells be sorted and cultured in vitro for phagocytosis assays to directly proof this hypothesis?
REPLY: Circulating monocytes, alveolar macrophages, meningeal macrophages and hepatic Kupffer cell macrophages express CD206, but not human splenic macrophages – neither the CD169+ nor the CD163+ macrophages. As Millard et al., (Cell Reports 37, 110058, November 23, 2021) report that all macrophages sorted did turn out not to be macrophages and we showed that in mice the anti-CD169 antibody inhibited bacterial uptake by CD169+ macrophages in vivo (Ercoli 2018), we decided not to sort cells for downstream analyses. We might resort in the future to selective depletion before cell culture. During revision of the paper we have identified the scavenger receptor class-A MARCO (macrophage receptor with collagenous structure) as the most likely receptor for capsulated pneumococci (Fig. 5D1). In addition, time-lapse microscopy documents mannose-dependent direct interaction of macrophages with sinusoidal endothelial cells providing further documentation for this two-step process (Figure 5E, Supplementary Figure 4).

Ex vivo organ perfusion. Was the >90% bacterial load removed exclusively by CD206+ cells? Without this piece of information, the results of this section are quite redundant. Can AMR pneumococcal strains be removed as well? Or pneumococcal strains with hyper capsulation, such as serotype 1?
REPLY: The paper documents, using both organ perfusion and cell culture, a two-step process; first bacteria are captured by sinusoidal cells and only after this step, bacteria are then phagocytised by macrophages. We have now defined also that the scavenger receptor MARCO in macrophages is involved in bacterial clearance. In addition, the timelapse microscopy indicates a direct effect of mannose on mobility of macrophages exclusively when adjacent or in contact to sinusoids adding to the narrative of a sequential event during bacterial clearance.

Can AMR pneumococcal strains be removed as well? Or pneumococcal strains with hyper capsulation
REPLY: In addition to testing type 2, 4, 5, 6A, 19F in perfusion assays, we now tested type 1, 2, 4, 5, 7F, 19F and 23F in primary cultures (Fig. 5B). The strains include clinical isolates with resistance to tetracycline, erythromycin, chloramphenicol, clindamycin, and penicillin.

Contribution of the mannose receptor on sinusoids to bacterial clearance in the human spleen. The sentence “In the human spleen, however, macrophages lack CD206 entirely” goes totally in contrast to the results described in the section “Cellular marker distribution in the human spleen” in which some macrophages are classified as “CD206+”. Quite surprising was the fact that, considering mannose receptor a ligand for pneumolysin (Subramanian et al, Nat Microbiol, 2020), the authors have not investigated the pneumolysin-mannose receptor binding as triggering factor for macrophage clearance activity; on the other hand, authors seem to focus primarly on capsulation. In vitro, this can be easily tested by pretreating macrophages with either sub-lytic concentration of pneumolysin or polysaccharide capsule, and assess the effect on phagocytic capacity. In addition, the results shown by the authors in Fig 4 should be performed with pneumolysin-deficient mutant to assess the role of pneumolysin-mannose receptor binding in macrophage response.
REPLY: We are aware of the paper on pneumolysin and cite it in our introduction. We have now tested a ply mutant. Data reported in the results and in Supplementary Figure 3 A show that absence of pneumolysin does not alter the CD206 linked mannose inhibition in our in vitro

system indicating that, at least in our model, the interaction between CD206 and capsule appears to be dominant.

Mechanism of pathogen clearance by both CD163+ and of CD169+ macrophages. Firstly, this section should be directly linked/merged with the section “Ex vivo organ perfusion”. This section seems to highlight the strong bacterial clearance activity performed by the less abundant CD163+ and CD169+ cells in the spleen, even though in the section “Cellular marker distribution in the human spleen” authors have speculated that the CD206+ cells should be the ones with higher capability for bacterial clearance. Clarity on this point should be given.
REPLY: There must be a misunderstanding. We state that in a two step-process first the CD206+ sinusoids bind pneumococci on the surface (no phagocytosis, no killing). In a second step bacteria are then taken over, phagocytised and killed by both CD163+ and CD169+ macrophages. We have revised the text. We also added data on the scavenger receptor class-A MARCO (macrophage receptor with collagenous structure) which appears to be involved in pathogen uptake by the CD163+ red pulp macrophages.

To assess the bacterial clearance capacity of CD163+ and CD169+ cells, compared to CD206+ cells, cells should be sorted, cultured and used for phagocytosis assays in vitro to measure bacterial killing ratio. Microscopy experiments can only provide a partial quantification of what is happening in a whole human organ.
REPLY: We have tried sorting of homogenised spleen cells and multiple cells appear to express both CD163 and CD206. This is an artefact representing cell rupture during sample preparation and homogenisation of the solid organ. Literature documents the issue that FACS sorting of spleen cells did not yield any macrophages when analysed by single cell sequencing but only fragmented cells which the sorter erroneously recognised and sorted as macrophages (Millard et al., Cell Reports 37, 110058, November 23, 2021). In this paper we have therefore chosen not to pursue this approach. Our data still allow quantitative and mechanistic conclusions.

In my opinion, concerning bacterial clearance, more relevant than caspase-3, authors should assess the lysosomal activity inside the splenic macrophages (lysosome intracellular activity) which is a more direct readout of the active phagocytosis activity inside lysosomes.
REPLY: Fig. 6C and 6D1-6D2 show analyses of the lysosomal associated membrane protein 1 (Lamp-1) as indication of lysosomal activity.

Additionally:
- The authors used mannose to inhibit the mannose receptor, mannose is a well known to cause an anti-inflammatory effect. E.g Torretta S, Scagliola A, Ricci L, Mainini F, Di Marco S, Cuccovillo I, et al. D-Mannose Suppresses Macrophage IL-1beta Production. Nat Commun (2020) 11(1):6343. doi: 10.1038/s41467-020-20164-6. Additonally cytokine data indicate a difference between mannose treated spleens and infected spleens (reduced IL-6, Il-1B)
- The authors should design experiments to assess if there is an effect of mannose on the resident immune population
REPLY: Torretta et al explore reactivity of murine LPS-activated BMDM macrophages to mannose which may likely act on the macrophages via the CD206 mannose receptor. In the human spleen, differently to the murine spleen, neither CD163 red pulp macrophages, nor the CD169+ arteriolar sheath macrophages express CD206. In humans, mannose is widely used

for the prevention of UTI (*JAMA Intern Med*. 2024;184(6):619-628) having an excellent safety profile (and a bit more doubtful efficacy) but its activity is not anti-inflammatory but supposed to reduce the interaction of fimbriae binding to mannose on epithelial cells. Oral mannose in humans is very rapidly excreted through the kidney and concentrates in bladder. In Supplementary Figure 6 we show cytokine concentration values during organ perfusion which show no difference between non-infected, infected and mannose-treated human spleens during ex vivo machine perfusion.

- The paper lacks a discussion paragraph on the limitation of the study
REPLY: A paragraph on the limitations of the paper has been added before the concluding paragraph.

- In some graphs limited info on n numbers, what each symbol represent, if the data is presented as SD or SEM
REPLY: we have included the missing information.

MINOR
No information is given on n numbers in each graph, statistical test used, nor if the bars represent SD (assumed based on text), or SM, the authors should provide this information Information on statistical test is needed, has Dunn multiple comparison always been performed? Is it an unpaired t-test? One-tailed or two-tailed? Was the data tested for normal distribution?
REPLY: we have included the missing information.

Has imaging analysis been performed blinded?
REPLY: Yes. Many images and thus data points have been analysed by at least two persons. As values and thus results are "dramatically" influenced by the setting of the cut-off values this was seen as essential. Main aim of the blinded evaluation was to check if those data found to show significant differences were the same. We have underlined this in the methods section.

Abstract: Lacks scientific phrasing, e.g labour
REPLY: changed to task

Results:
Line: 108: Can the authors clarify if there are different populations of sinusoid lining littoral cells, since most literature describe these cells as CD31+, but the authors comment that they do not express this
REPLY: We are well aware that splenic sinusoidal cells are described as weakly positive for CD31. But our microscopy data do not detect any tangible reactivity in splenic sinusoids. The sparse reactivity is more likely due to staining of capillaries (Supplementary Figure 1 C-D). In any case, we now tested phagocytosis inhibition using anti-CD31 antibodies and do not observe any inhibition. Data are shown in Fig. 5D.

Line 108. The authors don't show the co-localization of the markers to identify their cell population (e.g CD68 + CD163 and CD68 and CD169).
REPLY: In Supplementary Figure 1 A-B, we now show, that in our human spleen biopsy both CD163 and CD169 macrophages are positive to CD68

Supplementary figure 1 D1D2. There are some data points missing for HSP3, HSP50, have experiments not been performed for these? Additionally, mannose pre-treatment seems to have an effect on the density of CD163+ cells

REPLY: The data missing for HSP3 at time 0.5 are due to the inconsistent staining of a very small biopsy; these data were therefore low quality and data points were excluded from the analysis. Time zero data of HSP50 could not be acquired for the specific CD206 staining as this biopsy was finished before staining for sinusoidal cells. The mannose-treated spleens (HSP66 and HSP67) appear to have slightly lower CD163+ cell density than HSP50, but are overall comparable to HSP51. They also show no clear differences relative to the two control spleens (HSP3 and HSP63), with HSP3 exhibiting the lowest CD163+ density. These variations most likely reflect normal inter-individual differences in CD163+ cell density rather than any specific effect of mannose on RPMs abundance. Importantly, we are not proposing that all organs have identical densities of CD206+ or CD163+ cells; rather, our point is that within the same spleen, serial biopsies taken at successive time points post-infection show stable distributions of these markers.

LINE 118: This statement seems to not be supported by S1D1-S1D2, as there seems to be an increase in both D163+ and CD206+ upon infection, alternatively no info is given if these data has been statistically tested

REPLY: We are not proposing that all organs have identical densities of CD206+ or CD163+ cells; rather, our point is that within the same spleen, serial biopsies taken at successive time points post-infection show stable distributions of these markers. This stability excludes the possibility that the mechanisms we observe within each human spleen are due to time-dependent changes in cellular distribution.

Line140: What is the rational for using such a high CFU dose?

REPLY: As 1x10^7 are removed in less than 15 minutes from both perfusion liquid and organ, the 1x10^8 dose was the appropriate to test the dynamics of the system. It is important to underline that 1x10^8 in 500 ml is 2x10^5 CFU/ml which is a low/normal dose when infecting intravenously a mouse.

Figure 2: Please adjust the Y-scale, so the viewer can observe the data points in A1, A2, B1, B2, D1, D2, E1, E2. Its very difficult to analysis the differences between the strains and its elimination

REPLY: All graphs in figure 2 have the same log scale which is the unique option to plot data of greatly different magnitude on comparable graphs. We will share all raw data on the AMS Acta open data access depository of the University of Bologna, so the single data can be accessed and viewed.

Line 143: What about bacterial signal onto CD206+ cells?

REPLY: This is an important question, but we have no information on this aspect. We secured funding for multiple samples for 10x Visium HD gene expression profiling. This hopefully will deliver some information on the reaction of both sinusoids and macrophages to pathogen binding.

Line 160: Although the confocal images demonstrate the authors statement, this type of statement should be supported by literature or a Flow cytometric analysis on the macrophage

population. Additionally, the images seem overexposed, especially in the red channel. REPLY: Images shown for display are overexposed; however, quantitative analysis relied on manual thresholding of marker signals, for these reasons the analysis was independently performed by two operators to reduce operator-dependent bias. This has been carefully addressed. We have chosen not to display FACS data as it has been demonstrated that intact macrophages are absent in single-cell suspensions from spleen as they are disrupted during preparation (Millard et al., Cell Rep. 2021 Nov 23;37(8):110058. doi: 10.1016/j.celrep.2021.110058).

Line 174: The effect of mannose on the bacteria should be assessed with a growth curve, to confirm that the bacteria has a normal phenotype

REPLY: Results are now reported in Supplementary Figure 3 C and show that while growth on mannose is slower compared to growth on glucose (as expected), the addition of mannose to glucose-containing cultures does not change the bacterial growth profile.

Figure 3A1, A2. The authors have a much higher n number for mannose treated groups, this could significantly drag the data. Also the authors should state what each symbol represent since each figure is only quantified based on 2 spleen.

REPLY: Each symbol represents an individual spleen region ("stamp"), measuring 2772 × 2080 µm, analysed for each biopsy at each time point. The number of stamps analysed per group varies according to the size of the biopsy: larger tissue sections allow for more stamps to be examined, providing a more representative overview of the organ rather than focusing only on "preferred" areas. For these graphs, data from two different spleens were pooled for both the no-mannose group (HSP50, HSP51; white bars) and the mannose-treated group (HSP66, HSP67; grey bars). We will add these details.

Figure 3D1,D2 why are there data points missing in D2 0.5 grey bar compared to D1? REPLY: Apologies we didn't notice that. There were 4 missing numbers in graph 3D2. Now the numbers of the data points are identical between the two graphs.

Figure 3E1, the data is dragged by one outlier in no treatment group, has the data been analysed for outliers?, without this outlier, the bars look identical. The statement of CD169+ macrophages should be modified.

REPLY: we removed the outliers and repeated the statistic. The difference between the two groups at 0.5 hours post-infection disappeared, but it remained unaltered for the 2h and 5h time-points.

Line: 197: Have the authors addressed if there are dendritic cells in their culture, especially since these can express cd206.

REPLY: Human dendritic cells in the spleen are CD11c+ and CD209+ but not CD206+. Based on our staining, dendritic cells in the spleen are localised exclusively to the follicle and not to the red pulp. We therefore do not envisage dendritic cells intervening in the phenotypes described in the organs. We did not test for CD209 and CD11cexpression in the cell cultures.

Figure A2. The image is overexposed, better quality pictures should be provided

REPLY: For the purpose of visualisation colour is strong in images to be posted on figures. All raw data on the AMS Acta open data access depository of the University of Bologna which will allow individual visualisation of microscopy data.

Figure 4F, The data should be presented as CFU/ml or bacterial signal, not as % of dose

REPLY: We disagree, as the scope here is to show the level of killing of the initial dose of bacteria added to the macrophages/sinusoids. It is particularly important as it allows to visualise that Klebsiella already duplicates in the 30 min of incubation underlying the dynamic nature of the documented events.

Figure 5A. where is the quantification for time point 0? Does the staining not give any background?

REPLY: There are no bacteria at time 0. We have removed time point 0.

Figure 5B1 and B3, this is a very weak association and no information is given if this association has been tested statistically

REPLY: We apologise; the statistical analysis is now reported in methods and figure legend.

General comment:

If the CD206+ cells present the bacteria to macrophages, then are they also degraded during the phagocytosis of the bacterium?

REPLY: Time-lapse data now show that macrophages move on sinusoidal cells, likely scanning for bound bacteria without any damage to the sinusoids. It will be a task for the future to dissect the intimated details of these interactions.

Line272-278: These images should be included in the supplementary, and why data not shown?

REPLY: Images have been updated, time lapse analyses are shown in Fig 5E and Fig S4. The time lapse movies will be uploaded on the open access server AMS Acta.

LINE 284: The data does not support this statement: caspase-3 is also increasing in control spleens, to assess if there is an increase in caspase-3 upon infection the data should be compared to control spleen caspase-3 levels and a two-way ANOVA should be performed.

REPLY: yes, activated caspase-3 does increase in the control spleen as well, but the increase is 10x higher in the infected spleen. This likely indicates in the control organs a background induction of the apoptotic process over the very low time-0 values in these organs. We did not performed statistic between he groups because unluckily the presents different time-points.

Line 291-295: Where does the 0.65% number come from, the graphs indicate a 10% of CD163+ cells having caspase-3 signalling, how does that give 0.65% in the text? The authors should clarify

REPLY: apologies, this data comes from Supplementary Figure 3 D1 referring to the % of CD163 area occupied by caspase-3 signal, and we admit this is not clear from the main text. To estimate the proportion of apoptotic macrophages, we quantified the average tissue area positive for CD163 and cleaved caspase-3. Considering that a human spleen contains approximately $2x10^{10}$ macrophages (Sender et al., 2023) at 1-hour post-infection, approximately 0.065% of the $CD163^{+}$ area colocalized with cleaved caspase-3 (Supplementary Figure 3 D1) and given that caspase-3^{+} areas were roughly ten times smaller than the total $CD163^{+}$ regions, we estimate that ~0.65% of $CD163^{+}$ macrophages were positive for this apoptosis marker. This corresponds to roughly $7x10^{7}$ to $3x10^{8}$ caspase-3 positive macrophages.

Considering that the challenge dose used during ex vivo perfusion was approximately $1x10^8$ bacteria (MOI 0.0025–0.006), these data potentially support the hypothesis that each macrophage killing a pneumococcus may undergo apoptosis as part of the elimination process. We will change the figure reference in the main text.

Additional experiments:
1. Caspase-3 on CD206+ cells
REPLY: In sinusoidal lining cells, we do observe only bacteria on the surface without any evidence for internalisation, any change in the cell structure or motility during the timelapse. This matches to a lack of caspase-3 activation observed in these cells (not shown).

2. Mannose effect on the immune system itself without an infection
REPLY: The timelapse shows that macrophage mobility does not change with or without mannose if macrophages are not in contact/adjacent to sinusoids (Supplementary Figure 4). Safety data on the use of mannose for treatment or prevention of recurrent UTI indicate no effect on human immune system in non-septic conditions.

3. Primary cell culture purity (DCs)
REPLY: We have decided not to purify macrophages or sinusoids as reaction with antibodies used during cell sorting purification may influence behaviour (binding by anti-CD169 abolished intracellular replication; Ercoli 2018). Primary non-stimulated macrophages are not stable for over 24-48h in culture, hence we performed the least interventions possible and directly tested bactericidal activity.

4. Further proof or mechanistic explanation between CD206+ and macrophage interaction for bacterial clearance should be performed.
REPLY: Timelapse microscopy provides proof of interaction between CD206 and macrophages, as CD206-blocking stops mobility on macrophages adjacent to sinusoids (Figure 5E, Supplementary Figure 4).

5. To fully illustrate that the capsule is the main determinant of CD206+ capturing the bacteria, experiments with a pneumococcal strain that is hypercapsualted (serotype 2 for instance) should be compared to lower capsule containing strains.
REPLY: We have now tested serotypes 1, 2, 4, 5, 7F, 19F, and 23F and in all cases mannose blocks bacterial killing in primary cultures (Fig. 5B)

6. Address if a Ply mutant strain would bind the mannose-receptor of CD206+ cells
REPLY: We have tested a ply mutant and both mannose and anti-CD206 still inhibit phagocytosis. Data are reported in Supplementary Figure 3 A.

Reviewer #4 (Remarks to the Author):

Interesting data identifying a novel mechanism by which encapsulated S. pneumoniae and probably other bacterial pathogens are cleared by the spleen. This is clearly an area of high

importance and identifying a novel clearance mechanism is therefore of very high interest. Obtaining these data requires the ex vivo spleen model and is therefore extremely difficult.

Major issues/questions:

The two biggest issues that need addressing via text is (a) how does the mannose receptor recognise such a broad range of capsule structures not all of which contain mannose?
REPLY: The mannose receptor has multiple domains the cysteine-rich (CR) domain recognises sugars terminated in SO_{4-}3-galactose (Gal) or SO_{4-}3/4-*N*-acetylgalactosamine (GalNAc), the C-type lectin-like domain recognised mannose (Man), fucose (Fuc) and *N*-acetylglucosamine (GlcNAc), and the fibronectin type II (FNII) domain collagen (doi 10.1002/eji.200535685). Thus multiple sugar structures in addition to mannose are recognised by CD206.

And (b) how does the proposed handing on mechanism from MR206 expressing sinusoidal cells to other splenic macrophage populations actually work as my concept of splenic macrophage populations is that they are fixed in the tissue rather than mobile? I don't think we need more data on this at this stage but some discussion of these points would be important even if it is essential 'this is unclear….'
REPLY: Before running the timelapse of macrophages and sinusoids in coculture, we had a similar concept of immobile macrophages. The data of the 20 min timelapse confocal analyses show that splenic macrophages move in culture (Figure 5E, Supplementary Figure 4). Data show that presence of bacteria does not influence macrophage mobility. Also binding of macrophages to bacteria does not change their mobility. What instead completely limits macrophage mobility is the encounter with a sinusoid with a blocked CD206 receptor. Importantly mannose does not influence macrophage mobility if macrophages are not in contact to sinusoids. The details of these interactions will be addressed in follow up work.

The data on equal speed of clearance of different serotypes is interesting but (a) it does look like there could be differences at a the later timepoints that are not significant due to power issues; and (b) it is probably too far a leap to say these data for five serotypes means all serotypes are cleared equally well.
REPLY: In addition to the mix of five serotypes used in the organ perfusion we have now tested in primary culture types 2 and 4, types 1, 5, 7F, 19F, and 23F (Fig. 5B). We do not see a difference, which does not indicate that macrophage clearance is equal, only that differences in both our assays do not allow to document invasive disease potential.

Lines 223 to 269, and 291 to 341 seem to be discussion but are not subtitled as such; in fact, there is no formal discussion section and the results and not labelled as results and discussion. Also, lines 315 to 341 repeat earlier text. A separate discussion section would be helpful and allowed by Nat Comms. Any discussion needs to address how these results fit with the existing fairly extensive data on splenic clearance obtained using mouse models etc and whether there are major differences between species.
REPLY: We understand the issue raised by the reviewer, but would like to retain the current manuscript outline, which is in line with the options for manuscript formatting by Nature Communications.

Line 294 correspondence between apoptotic cell numbers and bacterial inoculum; what were

the results when the lower inoculum was used? Without these data I think the authors should be wary of drawing too much of a conclusion from the similar numbers.
REPLY: We have now tested apoptosis induction in biopsies with spleens receiving the 10-fold lower inoculum and data show 10-fold lower caspase-3 activation which matches the background values (Supplementary Figure 3 D4). This is not a formal demonstration, as caspase-3 levels at this inoculum fall below the reliable detection range, but the data are fully consistent with the hypothesized correlation between phagocytosis and apoptosis. We have tested caspase-3/apoptosis inhibition and in vitro this has no effect on the bactericidal activity of the macrophages, indicating that it occurs after bacterial killing.

Figure 5: it looks like in panels E to F that uninfected spleens have raised apoptotic markers at early timepoints including in some data higher than infected spleens at the same time point; this is not mentioned in the text and needs to be. The persistence of higher levels of apoptotic cells in the infected spleens then probably becomes the main point of these data, which is slightly different to how it is written at present
REPLY: In Fig. 5F1–F2, the control spleens show higher early M30 levels in both CD163$^+$ and CD169$^+$ macrophages compared with the infected spleens. However, it is important to note that in the control spleens, M30 levels never exceeded the values observed at the time-zero biopsy. This indicates that no activation or induction of apoptosis occurred relative to baseline throughout the entire perfusion. In contrast, the later increase in M30 levels observed in the infected spleens clearly follows the early rise in caspase-3 signal, consistent with the caspase-3–dependent generation of the M30 epitope.

Minor issues
Figure 1 legend is a bit light on detail; for example, what panels D to F are actually showing is not that clear. I think some measure of immunofluorescence per mm2 spleen?
REPLY: we thank for the comment and have addressed the issue.

Line 141: the meaning of ‘of five 140 equally counted serotypes’ is not that clear; equal inoculum sizes?
REPLY: we changed to “dosed”

Figure 2 panels AB E D I would favour reducing the y axis so the curves can be seen rather than keep the y axis spread the same across all panels
REPLY: We prefer to keep the same axes as it helps comparing data across graphs.

Lines 157-64 largely repeat introduction text and can be reduced
REPLY: we changed the text

Lines 174-76 the authors could just grow the bugs in the perfusate +/- mannose to prove the point that mannose does not increase S. pneumoniae replication?
REPLY: Growth curve in mannose, glucose, and mannose with decreasing glucose concentrations have been added in Supplementary Figure 3 C which confirms no impact of mannose on pneumococcal growth.

Figure 4; did the authors identify any CD169 cells?

REPLY: In cell culture, very few CD169+ cells are detectable. An image was added in Supplementary Figure 5 D.

Figure 5 panel C; black bars are the grey columns? And the five different sets of grey bars represent five different spleens? If correct, this needs stating in the legend and the spleen donor number added to the x axis. If each grey section is data from a separate spleen in panel C, are the data in panels D to F pooled from multiple spleens or just one?? And , if I have completely misinterpreted these panels then please make it much clearer what they represent. Title for this figure; it does really contain bactericidal data? Replace with something about apoptosis in the title to the legend?
REPLY: We have checked and revised figures. C) black bars are actually the dark grey bars, and each set represent a different spleen, we can add the respective name on the x axis. D to F) the data pooled multiple spleen for each group, see Supplementary Table 5 for the list of the spleens used to generated each single data set. This figure is now named Figure 6.

REVIEWERS' COMMENTS

Reviewer #1 (Remarks to the Author):

I thank the authors for answering my questions in a comprehensive fashion. I am particularly gratified that the experiments I suggested yielded data of great interest and have added to the manuscript. I think the paper should now be acceptable for publication in Nature Communications.

REPLY: we thank reviewer 1 for the helpful comments

Reviewer #2 (Remarks to the Author):

I co-reviewed this manuscript with one of the reviewers who provided the listed reports. This is part of the Nature Communications initiative to facilitate training in peer review and to provide appropriate recognition for Early Career Researchers who co-review manuscripts.

REPLY: thank you for your review

Reviewer #3 (Remarks to the Author):

I appreciate the effort of the authors to address most of the comments. However, there are a few things they disagree on, such as:

1.Authors state that the pictures needs to be overexposed in order to be viewed on paper, but overexposure is a major source of artifacts in microscopy.

REPLY: We fully agree that overexposure can introduce artifacts and must be avoided during quantitative image analysis. To clarify, only the representative images prepared for figure display were adjusted to optimize visualization of the different fluorescence channels for print reproduction. All quantitative analyses were performed on the original, non-overexposed raw image files. When analysing the data, thresholds were carefully defined on the raw data using standardized methods and the images analysed by at least two different operators to allow reliable data analysis.

2. Flow cytometry cannot be done according to the authors, which I do not see any valid reason why.

REPLY: We agree that flow cytometry is in principle a powerful method for immune cell characterization. We performed multiple attempts to obtain reliable single-cell suspensions from spleen homogenates and carefully evaluated flow cytometry analysis in two independent facilities.

Despite stringent single cell gating and staining for the macrophage marker CD163 and the sinusoidal marker CD206, we consistently observed 10–30% double-positive events. However, based on our primary culture experiments and tissue microscopy analyses, as well as previous reports (e.g. Steiniger et al., 2015, doi:10.1111/imm.12469), these cell populations are not biologically expected to co-express both markers in situ.

Furthermore, as reported by Millard et al. (Cell Reports, 2021, DOI: 10.1016/j.celrep.2021.110058), macrophage populations isolated from spleen homogenates and subjected to downstream single cell-sequencing analyses were shown to contain

substantial misidentified cell populations, highlighting technical challenges associated with generating reliable single-cell suspensions from spleen tissue. In an earlier example, many of the cells present in mouse lymph node cell suspensions that stain for the subcapsular sinus macrophage marker CD169 are not macrophages but lymphocytes that have acquired subcapsular sinus macrophage-derived membrane blebs likely caused by fragmentation during tissue preparation (doi.org/10.1371/journal.pone.0038258). In light of these considerations and given the discrepancy between our flow cytometry results and in situ observations, we decided not to rely on flow cytometry data for this study, as this approach did not allow us to draw unequivocal conclusions.

3. Reporting of bacterial CFU, which is very important.
REPLY: we have revised

Reviewer #4 (Remarks to the Author):
I have no major queries about the resubmitted version
a few minor issues
Line 74: the full use of bacterial names after first use needs revising to the abbreviated form
REPLY: thanks - we have revised
line 302 missing a 'the' - '...the presence CR-301 ligands has been linked to receptor-mediated antigen transfer...'
REPLY: thanks - we have revised
Figure 2 panel C1 is labelled No mannose' but I think is with mannose?
REPLY: thanks - we have revised

Reviewer #5 (Remarks to the Author):
I co-reviewed this manuscript with one of the reviewers who provided the listed reports. This is part of the Nature Communications initiative to facilitate training in peer review and to provide appropriate recognition for Early Career Researchers who co-review manuscripts.
REPLY: thank you for your revision
